# Whole Transcriptome of the Venom Gland from *Urodacus yaschenkoi* Scorpion

**DOI:** 10.1371/journal.pone.0127883

**Published:** 2015-05-28

**Authors:** Karen Luna-Ramírez, Verónica Quintero-Hernández, Víctor Rivelino Juárez-González, Lourival D. Possani

**Affiliations:** 1 Australian Venom Research Unit, Department of Pharmacology and Therapeutics, University of Melbourne, Victoria, Australia; 2 Departamento de Medicina Molecular y Bioprocesos, Instituto de Biotecnología, Universidad Nacional Autónoma de México, Cuernavaca, Morelos, México; The City University of New York-Graduate Center, UNITED STATES

## Abstract

Australian scorpion venoms have been poorly studied, probably because they do not pose an evident threat to humans. In addition, the continent has other medically important venomous animals capable of causing serious health problems. *Urodacus yaschenkoi* belongs to the most widely distributed family of Australian scorpions (Urodacidae) and it is found all over the continent, making it a useful model system for studying venom composition and evolution. This communication reports the whole set of mRNA transcripts produced by the venom gland. *U*. *yaschenkoi* venom is as complex as its overseas counterparts. These transcripts certainly code for several components similar to known scorpion venom components, such as: alpha-KTxs, beta-KTxs, calcins, protease inhibitors, antimicrobial peptides, sodium-channel toxins, toxin-like peptides, allergens, La1-like, hyaluronidases, ribosomal proteins, proteasome components and proteins related to cellular processes. A comparison with the venom gland transcriptome of *Centruroides noxius* (Buthidae) showed that these two scorpions have similar components related to biological processes, although important differences occur among the venom toxins. In contrast, a comparison with sequences reported for *Urodacus manicatus* revealed that these two Urodacidae species possess the same subfamily of scorpion toxins. A comparison with sequences of an *U*. *yaschenkoi* cDNA library previously reported by our group showed that both techniques are reliable for the description of the venom components, but the whole transcriptome generated with Next Generation Sequencing platform provides sequences of all transcripts expressed. Several of which were identified in the proteome, but many more transcripts were identified including uncommon transcripts. The information reported here constitutes a reference for non-Buthidae scorpion venoms, providing a comprehensive view of genes that are involved in venom production. Further, this work identifies new putative bioactive compounds that could be used to seed research into new pharmacological compounds and increase our understanding of the function of different ion channels.

## Introduction

Scorpions are arthropods belonging to the group of arachnids that have been living on this planet for over 400 million years [1]. Currently, around 1500 living scorpion species have been described [2]. Millions of years of evolution have resulted in a high degree of specific and efficient scorpion venom components. These venoms are true arsenals, containing important biomolecules selected for the immobilization of prey and serving in defense against predators.

Scorpions are classified in 18 families [3], the Buthidae family being the most comprehensively studied thus far. The family contains 30 different genera of scorpions dangerous to humans. Scorpion venom possesses different classes of toxins that mainly modify the function of ion channels and receptors in excitable membranes [4–7]. In addition, scorpion venom possesses a great variety of components: salts, nucleotides, biogenic amines, enzymes such as: phospholipase, hyaluronidase, L-aminoacid oxidase [8], metalloproteinase [9], serine-protease, mucoproteins; toxic peptides, proteins and antimicrobial peptides active against bacteria, fungi, yeast and viruses. Examples of the latter are: mucroporin-M1, which inhibits the amplification of the hepatitis-B virus and peptide Kn2-7, which possesses anti-HIV-1 activity [10, 11].

To date, 24 transcriptomes and proteomes have been reported on Buthidae scorpions [12–37] whereas only 15 studies have been performed with non-Buthidae scorpions, which are not dangerous to humans [19, 21, 38–49]. Non-Buthidae venoms contain a low percentage of sodium channel specific toxins. Interestingly, and contrary to the Buthidae scorpions, non-Buthidae venoms have a high number of antimicrobial peptides [50], potassium channel toxins, calcins and peptides with anti-malarial activity. Recently, a peptide named scorpine, isolated from *Pandinus imperatus*, was used successfully as an anti-malarial agent in biological models [51, 52].

An estimated total of 300,000 different peptides are present in the venom of extant scorpion species. Approximately 1% of these scorpion components have been characterized, [53] the majority of these being toxins. This is understandable because of the medical interest. Important efforts have been focused at identifying components responsible for human envenomation, and to a lesser extent for structure-function studies required to recognize toxin targets. This focus on toxins has left many potentially bioactive venom compounds unexplored.

As early as 1967, Rochat *et al* reported the isolation and characterization of several toxic peptides from the venom of a Buthidae scorpion [54–56]. Nowadays, scorpion toxins are still being characterized biochemically and pharmacologically in order to determine the number of proteins in the venom and their bioactivity. In the 1980s, electrospray ionization mass spectrometry (ESI-MS/MS) increased the speed of the task of venom characterization. High-throughput protein identification techniques by mass spectrometry allowed the proteomic analysis of venoms and facilitated the identification of hundreds of unknown different molecular weights components.

Several studies have reported complete mass fingerprinting of venom using proteomic analysis of venom components [57]. It is conceivable though, that not all components were identified using this technique given that some components are present in venom at very low concentrations [41]. This reveals the power of a venom gland transcriptomic analysis: all protein content and toxin-like peptides are potentially identified. [58]

Studying venom gland components at the transcriptomic level was made possible by the advent of the polymerase chain reaction. Studies have been performed using cDNA libraries of scorpion venom glands, which allowed for the identification of many venom components. However, the cDNA libraries that have been constructed with milked venom glands [15, 17, 32, 39, 40, 43, 45, 46, 48] and the ones constructed with “replete” venom glands [18, 37, 41] have reported only a few complete sequences (tens of genes). These genes code mainly for toxic peptides, antimicrobial peptides and in rare cases, for genes involved in cell regulation and metabolism.

Whole venom gland transcriptomes can now be produced with high-throughput sequencing technologies such as Next Generation Sequencing (NGS) (also called RNA-seq). Several platforms using NGS (454 pyrosequencing, Illumina (SOLEXA) sequencing, SOLiD sequencing, ion semiconductor sequencing, DNA nanoball sequencing) have proven to be powerful tools for research in genome sequencing, miRNA expression profiling and especially *de novo* transcriptome sequencing of non-model organisms [59–65]. NGS is a low-cost sequencing alternative capable of producing thousands or millions of sequences at once. The resulting dataset reveals information about genes that code for toxins, peptides with pharmaceutical interest and other components among which are enzymes and housekeeping genes present in the venom gland.

In the work presented here, NGS Illumina sequencing was used to perform a *de novo* assembly of the transcriptome of the venom gland of the scorpion *Urodacus yaschenkoi*. The aim of this study was to characterize in depth the complete set of mRNA transcripts present in the venom gland of a non-Buthidae scorpion. A further aim was to correlate this data with the already reported venom proteome and compare it with the cDNA library shotgun approach previously constructed by our group [41].

The coverage of the transcriptome was found to be 8.4 Gb, revealing hundreds of genes involved in the process of venom making. Several subfamilies of scorpion toxins and hundreds of genes related to biological processes, molecular functions and cellular components were identified. Further, we report 210 venom transcripts with full-length coding sequences assumed to code for 111 unique venom compounds, among which there are sequences that code for venom toxins, peptides and venom-specific proteins.

Finally, a comparison with the transcriptome of *Centruroides noxius* [16] and with the reported genes of *Urodacus manicatus* [49] was made. The comparison with *C*. *noxius* transcriptome revealed that components involved in biological processes, molecular function and cellular components are conserved between these species. The toxins however, are very different in a Buthidae and in an Urodacidae scorpion (non-Buthidae). Conversely, the toxins reported for *U*. *manicatus* are of the same subfamilies of toxins found in *U*. *yaschenkoi* scorpion. This dataset will contribute to the public information platform to accelerate studies in venomics research.

## Material and Methods

### Sample collection and RNA extraction

The *Urodacus yaschenkoi* specimen was obtained from the Australian desert on New South Wales close to Nanya (GPS coordinates -33.22422, 141.306059) on May 2011. The captured organism was taxonomically identified according to Koch [66] and maintained in a plastic box with water *ad libitum* and was fed fortnightly with crickets.

The total RNA was extracted from a flash frozen (immediately frozen in dry ice) ‘replete’ venom gland with the Animal Tissue RNA Purification Kit from Norgen Biotek Corporation according to the manufacturer’s instructions. The quality of the RNA was verified using a 2100 Bioanalyzer (Agilent Technologies).

### cDNA Library preparation and sequencing

The cDNA library for the high-throughput sequencing was made with Illumina TruSeq RNA Sample Preparation Kit.

The cDNA library preparation consisted on the following steps: i) mRNA enrichment and fragmentation, ii) cDNA synthesis, iii) paired- end and adaptors ligation (adenylate 3’Ends), iv) PCR amplification (DNA enrichment) and v) High-throughput sequencing (RNA-Seq method, Illumina Next-Gen sequencing technology).

The total RNA was purified to obtain the poly-A containing mRNA molecules using oligo-dT-attached magnetic beads applying two rounds of purification. During the second elution of the poly-A mRNA, the RNA was also fragmented using elevated temperatures (94°C) and primed for cDNA synthesis. Then, first strand cDNA was synthesized by reverse transcription (using SuperScript II reverse transcriptase) of the cleaved RNA fragments primed with random hexamers. Immediately, the RNA template was removed and the double-stranded (ds) cDNA is synthesized (using DNA polymerase I, dNTPs and RNA displacement with RNAse H). Ampure XP beads were used to separate the ds cDNA from the second strand reaction mix. The ds cDNA was subjected to end repair by converting the overhangs resulting from fragmentation into blunt ends, using an End Repair (ERP) mix. A single ‘A’ nucleotide was added to the 3’ ends of the blunt fragments to prevent them from ligating to one another during the adapter ligation reaction. A corresponding single ‘T’ nucleotide on the 3’ end of the adapter provided a complementary overhang for ligating the adapter to the fragment. Finally, DNA enrichment was done using PCR to selectively enrich those DNA fragments that have adapter molecules on both ends and to amplify the amount of DNA in the library. The PCR was performed with a PCR primer cocktail that anneals to the ends of the adapters. The purified cDNA library was used for cluster generation on Illumina’s Cluster Station and then sequenced using High-throughput RNA-sequencing (Illumina Next-Generation sequencing platform) on Illumina HiSeq 2000 following vendor’s instruction.

### Assembly and analysis of transcriptome

The raw sequencing intensities were transformed by base calling into sequence data using Illlumina’s RTA software, followed by sequence quality filtering using GELRAD (Illumina). Paired-end reads were 100 nt in length. The extracted sequencing reads were saved as fastq files (SRA accession number SRP045734). Adaptor fragments were removed from the raw reads to yield the clean read required for the analysis. *De novo* transcriptome assembly of these short reads was performed using Trinity RNA seq software (http://trinityrnaseq.sourceforge.net/).

First, the RNA-seq was assembled into the unique sequences of transcripts, often generating full-length transcripts for a dominant isoform. Then the assembled sequences were clustered. Each cluster represents the full transcriptional complexity for a given gene (or sets of genes that share sequences in common). These were designated as contigs. Then a further assembly step followed rendering unique gene sequences that were designated as unigenes.

### Abundance estimation and quality control

To estimate transcripts abundance, raw reads were mapped back to the assembled contigs using the Tophat/Cufflinks suite (http://tophat.cbcb.umd.edu/ and http://cufflinks.cbcb.umd.edu/). TopHat is a fast splice junction mapper for RNA-Seq reads. It uses Bowtie (ultra high-throughput short read aligner) to align RNA-Seq red to mammalian-sized genomes then analyzes the mapping results to identify splice junctions between exons. Cufflinks assembles transcripts, estimates their abundances, and tests for differential expression and regulation in RNA-Seq samples. To generate potential novel transcripts, Cufflinks was run without known reference transcripts. The relative abundance of the transcripts is based on how many reads support each one. Expression level was estimated and presented in FPKM (fragments per kilobase of transcript per million fragments mapped).

The quality of raw sequence data was assessed with FastQC software (http://www.bioinformatics.babraham.ac.uk/projects/fastqc/) and with CLC bio software. Eleven parameters were measured to assure the library construction, sequencing and *de novo* assembly were well done. At the same time, basic statistics of reads and detection of sequencing errors were obtained.

### Bioinformatic analysis

The assembled contigs were first blasted against a database containing only toxins from the scorpions *Hadrurus gertschi*, *Opisthacanthus cayaporum* and *Tityus discrepans* created *in situ* and collected from the NCBI non-redundant (nr) database. These annotated proteins were aligned to the assembled contigs to identify the homologous genes in *Urodacus yaschenkoi* using TBLASTN (E-value < 0.1). The main purpose at this stage was to verify if the assembly was correct. Then, the whole set of unigenes were blasted with tBlastn and later with NCBI-nr BlastX (E-value <10^-5^) to search identity or similarity. The sequences presenting hits in these databases were analyzed with Blast2go software (www.blast2go.com); the Gene Onthology (Go terms) was obtained as well by this mean. In brief, the bioinformatic analysis used for the assembled transcriptome was as follows: first, the 243,870 assembled sequences (contigs) were searched against an *in situ* scorpion toxin database. Once confirmed that the assembly in fact rendered hits for scorpion toxins, then the whole set of unigenes (62,505) were searched against tBlastn and then with NCBI-nr BlastX (E-value <10^-5^). Later, all the unigenes were analyzed using Blast2go with the default parameters to find the Go terms. Finally, a sub-dataset was created with the unigenes that gave hits with sequences reported in GenBank related with venom and housekeeping genes. The annotation was made using Blast2go software.

In parallel, a second sub-dataset of sequences having hits only with toxins and venom components of any species were then used to extract the coding DNA sequence (CDS) and identify their mature sequence. Sequences of this second sub-dataset were deposited in GenBank (EST database: dbEST JZ818592—JZ818692) and analyzed as follows: nucleotide sequences were translated to obtain precursor peptides using ExPasy-Translate tool program (http://web.expasy.org/translate). The signal peptide was predicted with Signal P 4.0 program (http://www.cbs.dtu.dk/services/SignalP/) and the propeptide was determined by using Prop 1.0 software (http://www.cbs.dtu.dk/services/ProP/). The theoretical monoisotopic molecular mass of putative mature peptide was obtained using ProtParam (http://web.expasy.org/protparam).

Furthermore, the presence of post-translational modifications (amidation and disulfide bridges) was determined manually by comparison within other scorpion toxins or cytotoxic (antimicrobial) peptides. Multiple alignments were performed with CLUSTALX v2.0 and the percentage of identity was determined with DNA Strider 1.3.

### Comparison with *Centruroides noxius* transcriptome and with *Urodacus manicatus* sequences

The sequences having hits in the NCBI database were compared with the transcriptome of *C*. *noxius* [16] using Geneious software and the tool ‘map to reference’ with strict parameters (high sensitivity/medium speed and 5 iterations with 35 bp of overlap). The same criteria was followed to compared the 19 genes reported for *U*. *manicatus* (GenBank accession numbers: GALI01000001-GALI01000019) [49]. The similar sequences obtained were then manually analyzed to find the CDS and ORF and were used to build alignments with the already identified *U*. *yaschenkoi* sequences.

## Results and Discussion

### *Urodacus yashenkoi* venom gland transcriptome sequencing output

To comprehensively cover the *U*. *yaschenkoi* venom gland transcriptome, the total RNA of the venom gland was extracted, and the mRNA was isolated, enriched, fragmented and reverse-transcribed into cDNA. The cDNA was sequenced on Illumina HiSeq 2000 and the resulting sequencing data were subjected to bioinformatics analysis.

After sequencing, 83,812,864 raw reads were obtained. After removal of adaptors, ambiguous reads and low quality reads the sequenced data resulted in 83,808,178 mappable reads. The average length read was 101 nt. The transcriptome size was equal to 8,464,625,978 nucleotides (8.4 Gb).

The clean and high-quality reads were assembled *de novo* using Trinity software, and resulted in 67,026,681 mapped reads and 243,870 assembled contigs ranging from 109 to 15,222 bp length being the mean assembled length of 260 bp. The number of unigenes found was 62,505 with an N50 of 1,139 bp (i.e. 50% of the assembled bases were incorporated into contigs of 1,139 bp or longer). A summary of the Illumina sequencing results and assembly output is outlined in Table 1. The size distribution of the contigs from *Urodacus yashenkoi* venom gland is shown in Fig A in S1 file.

**Table 1:** Summary of assembly statistics after Illumina sequencing.

	Sequences (nt)	Length (bp)	Mean length	N50 (nt)
Raw reads	83,812,864		101 nt	
Mappable reads	83,808,178			
Mapped reads	67,026,681			
Assembled sequences (contigs)	243,870	109–15,222	260 bp	
Unigenes	62,505		727 bp	1,139

The Quality Control report (QC) showed that the length distribution of most sequences (more than 99%) were 100–101 bp, no ambiguous base-content in 98.93% of the sequences and the coverage (number of sequences that support the individual base position) was 100%. Twenty-four 5’-end of the sequence was found multiple times but their particular percentages were not more than 0.5%; in most cases, no identity was found. In general, these data means that the assembly was well performed.

### Bioinformatic analysis

A total of 3900 sequences (unigenes), that gave hits with sequences reported in GenBank related with venom and housekeeping genes, were obtained (see Materials and Methods). These sequences subsequently were analyzed manually to select only those sequences that codify toxins, antimicrobial peptides and venoms specific components. Further analysis encompassed the determination of the CDS and delimitation of sequence precursors, identification of signal peptides, propeptides, mature peptides and posttranslational modifications (Table 2). Additionally, the theoretical mass was calculated and conserved domains were found. By this mean, 210 sequences coding for 111 unique amino acid sequences including venom toxins and proteins involved in venom production were comprehensively identified with all the parameters above mentioned (see Table 2). The sequences identified belong to the following subfamilies of known scorpion toxins: α-KTx (alpha-type of K^+^-channel specific peptides), β-KTx (beta-type of K^+^-channel specific peptides), calcium-channel toxins (calcins), ascaris-type protease inhibitor peptides, venom proteins, several enzymes, antimicrobial peptides, sodium-channel toxins, toxin-like peptides, venom allergens and La1-like peptides. This shows that the identified sequences comprise a wide array of diversity in venom components.

**Table 2:** Unique sequences encoded by 210 transcripts of the *Urodacus yaschenkoi* transcriptome.

Seq. Name	Fasta Sequence (ORF) amino acids	Seq. Description (BLAST)
comp234_c0_seq1	MKLINLMPVFLMLLIVVDYCHS **FPFLLSLIPSAISAIKRL** *GKRSAKSQQYVDLQKQDLNPDLDFDLDDLEELLDKLSDSDY*	antimicrobial peptide
comp17_c0_seq1-4	MKNQFVLLLLAIVFLQLISQSDA **ILSAIWSGIKSLF** *GKRGLKNMDKFDELFDGDFSQADLDFLRELTR*	**antimicrobial peptide UyCT3 ndpb precursor**
comp17_c0_seq5	MKNQFVLLLLAIVFLQLISQSDA **ILSAIWSGIKGLL** *GKRGLKNADRLDELFDGDISDADLDFLRELTR*	antimicrobial peptide ct5-ndbp- precursor
comp31_c0_seq1-4	MKTQLAFLAITVILMQMFAQTEA **GFWGKLWEGVKNAI** *GKRGLRNLDDVDDLFDSGLSDADDLFDSGLSDADDLLDSIFADLDA*	**antimicrobial peptide UyCT1 ndbp precursor**
comp192_c0_seq1-2	MKNQFAILLLAVVFLQLISQSDA **FLSTIWNGIKGLL** *GKRGLSNLDQLDELFDGDVSDADLKFLRELMR*	antimicrobial peptide pantinin 3 precursor
comp1267_c0_seq1	MNAKVMLVCLLVTMLVMEPAEA **GIWSWIKKTAKKVWNSDVAKKLKGKALNAAKDFVAEKIGATPAEAGQIPFDEFMNVLYS**	antimicrobial peptide c22 precursor
comp3813_c0_seq1	MQFKTLLVIFLAYLIVTDEAEA **FWGFLAKAAAKLLPSLFSSNKNSSKRK** *REIEDFYDPYQKDLDSELERLLSQLQ*	antimicrobial peptide
comp588_c0_seq1	MAKHLLAEFLVIMLISSLADG **KTTVGQKIKNAAKKVYNKAKDLIGQSEYGCPMVSTFCEQFCKMKKMNGDCDLLKCVCT**	beta-ktx-like peptide
comp17858_c0_seq1	MLLYRFNMASLSLVICIMGAIWTVGRQ **SKYPGFFPMDENGEVYRCDRLGYNFFCNATCVFQGGTYGYCAISSCFCENFTLPVAVSDNLG**	beta-like toxin tx651
comp18425_c0_seq1	MG**KNPGYLLCLPTVQIAPMMQITSERDAILKRYNSIAVLAIQSILHNCQ**	beta-like toxin tx651
comp35_c0_seq1	MKTQLAFLAITVILMQMFAQTEA **GFWGKLWEGVKNAI** *GKRGLRNVDQIADLFDSGLSDADDLFDSGLSDADAKFMKMFM*	**antimicrobial peptide UyCT1 ndbp precursor**
comp35_c0_seq2	MKTQLAFLAITVILMQMFAQTEA **GFWGKLWEGVKNAI** *GKRGLRNLDDVDDLFDSGLSDADAKFMKMFM*	**antimicrobial peptide UyCT1 ndbp precursor**
comp3842_c0_seq2	MKPNLVLASLAFLILCSVLEKCTA **QSGGRGRCRGRGEVFTYCGTGCRLTCQNYRNPPQICTLQCFIGCVCRSGWVRDTRSGRCVRPSQCRR**	Ascaris-Type protease inhibitor peptide
comp4363_c0_seq1	MKGTLVVFAFASLCFC **SVFEKYGANGGFETFIIPPGECYRYPGEEVRKCGSACPITCNNYRRYPVPCTKQCVHGCFCIPGLVRDIRSRRCLKPTQCP**	Ascaris-Type protease inhibitor peptide
comp5534_c0_seq1	MAKIAVFGIMLSVLVLAQA **FPQNYQPFECNEDEVFVPCLSPCRRTCKNLSPYPCTRLLPVCVSGCGCKAGRILDNATGKCVLPRDCTR**	Ascaris-Type protease inhibitor peptide
comp75842_c0_seq1	**RPGEVFTECGTTCPLTCNNYWNPPRVCPFNCFRGCQCRNGLVRNTRTGACVRPSQCRR**	Ascaris-Type protease inhibitor peptide
comp1136_c0_seq1	MKYVASFLIVLFAFFVLEDGMVEA **GFGCPLNRYQCHSHCLSIGRRGGYCAGFLRTTCTCYKNK**	antimicrobial peptide defensing
comp3700_c0_seq1	MRHLAFLLVVLIAFSVLEDGMVEA **GFGCPLNSYRCHARCKSIKRRGGRCGGFLNFQCICFR**	antimicrobial peptide defensin
comp2227_c0_seq1	**MRRLPEYVLVLVFACFVGLIVTTDDRLHVPEAASKRCARKPAGFVSVKTSGDNGFKIKVSGDVQHYIPGEMYTVSLQGYRTQFSVQKFTGFMLVVEPSDPLQSFSTTERSNGMFQLLADGLSKLSETCLNAVVHTSNVPKSDIQVLWLAPPAGTGCVVFRATVIENRELWYMDDGGLTLELCEEGPPEVVGECCACDEAKYEVTFEGLWSRFSHPKYFPTNEWLTHFSDIIGASHTADFRIWELDNYASEGIRQVAEWGATKKLESELNAEIDKIRTIIKARGLWYPNVTGKTFAVFRVDSKKHLVSLISMLGPSPDWIVGVSGLELCLKNCSWITGKVINLYPIDAGTDSGVSYISPNSPTVPQEKIRQITSSYPKNDLSPFYDATGAPMKPIAKLTITRERVYTRTCNSAGGSATREPPSALTPEEPEDDLNRPECQVTQWSEFSPCSVTCGEGIRMRNRKYLMEKKAQMMNCVVQLVEKELCEPECSVGFSCETINWSAWSECSVTCGKGVRMRNRRYVNSMSRKTCTLSLVEKEMCTGDVPECEQKEVIDANCAVTQWSEWSPCTVTCGKGMKIRTRLYFDPSSLDTCNVELIQKMLCMADRTDCSIDPAEAKEICMQPKETGACRGYFPRWHFDLSRRECVQFIYGGCRGNRNNFERYSDCNQMCSMMVRGLPSTIATLAASPIQVPNVTEAPPVNCMVTPWSPWSACSRTCGNGRKERKRMIKVAPLNGGKPCPRRLTQRRRCKDLPQCSVDCMVTPWGAWSACSTTCGQSSTQQRTREIKRPAKHGGAPCGPRVERRFCSIPLCTY**	Venom protein f- Spondin-1-like
comp120806_c0_seq1	**VMFYEYALGKYPKIDSNKVDINGGLPLLGNLDEHLMQAERDIVKIVPNPNFNGLGVIDWEAWRPTWEYLWGSLSIYKNRTLELVRVMHPSSPDNFVQDIAKTIWEDSAKQW**	hyaluronidase-1 isoform 1 (partial)
comp7071_c0_seq2	**KITCFQTKLISKTDLSFQHHFWKMLAITASMGLDGAVIWGSSDYFSEKTKCEELEVYINDVIAPAVTTVSSNANRCSKEVCNGNGRCTWPSEPFTSWKYLADTQLQRRDPVNIVCRCQTGEGRYCN**	hyaluronidase-3 isoform x3 (partial)
comp7071_c0_seq1	**KIVMFYEYALGKYPKIDSNKVDINGGLPLLGNLDEHLMQAERDIVKIVPNPNFNGLGVIDWEAWRPTWEYLWGSLSIYKNRTLELVRVMHPSSPDNFVQDIAKTIWEDSAKQWMSKTLRLAKKLRSDGMWCYYLFPDCYNYGGKDHPSEFSCGEKIRRSNDELSWMWNKSSALCPSIYFSGLQINYNESQRTWFLQAKLAEAVRVSRPHTKIYPFINYLVHDSRTPVPVHHFWKMLAITASMGLDGAVIWGSSDYFSEKTKCEELEVYINDVIAPAVTTVSSNANRCSKEVCNGNGRCTWPSEPFTSWKYLADTQLQRRDPVNIVCRCQTGEGRYCN**	hyaluronoglucosaminidase 1 (partial)
comp1933_c0_seq1	MWFRLVLFCVLVTSIYS **LSCPCWRDRASYCGPPPTNCPVGLTDDACGCCKVCAKAEGEICGGPWGTSGRCAEGLTCVKPDNVEEFIRNQIDGVCKKEKQ**	Venom protein insulin-like growth factor-binding protein 7
comp1991_c0_seq1-5	MNNIRFAVMLVFLMVLAVGGLSA **KYAPTGGCPLSDALCARYCLKHNYGRSGKCDGSTCKCS** *TKLPNIIVL*	Alpha-KTx precursor
comp2092_c0_seq1	MNKTLCTIFLVVLVMFAISVLPAES **IGGCPIDSMCKSYCKNHKYGSEGKCDGTNCKCSL*G***	Alpha-KTx precursor
comp12_c0_seq1	MERILKPVFLAILIVLSFSSQCMG **FGESCQAGKHIVPVGQQQIDSSTCTLYKCSNYNRKYALETTSCATLKLKSGCRMVPGAATAPFPNCCPMMMCK*G***	la1-like protein 13 precursor
comp13_c0_seq1	MKHLSDAVFFFVCLSICALFSLTLC **DGEICQVGSMAIPVGKEQPDPKGCAKYECLSQSNRVLIKKVTCASQALKRGCKSVPGPAGKRFPECCPTTLCRGKQWGQ**	la1-like protein 15 precursor
comp3687_c0_seq1	FSLVWAFACVLTYVLVTEVNIDNYRPSCSGNFYTIHSLTFQV**VSEICTAGKIIIPLNEEKQDPETCALYKCTKYAGRIVLITVTCAPQEPRRGCRNVDSPVDAPFPDCCPIVLCKVYELGGK**	la1-like protein 15 precursor
comp3687_c0_seq2	MDKSAIVILVSLGVCLCFDLCSG **YGEICTAGKIIIPLNEEKQDPETCALYKCTKYAGRIVLITVTCAPQEPRRGCRNVDSPVDAPFPDCCPIVLCKVYELGGK**	la1-like protein 15 precursor
comp42_c0_seq1	MNTKFTVLIFLGVIVASY **GWITEKKIQKVLDEKLPNGFIKGAAKAVVHKLAKSEYGCMMDISWNKDCQRHCQSTEQKDGICHGMKCKCGKPRSY**	antimicrobial peptide opiscorpine3-like precursor
comp324_c0_seq1	MQTQCTVLQLLVLVALCSC **GGILKEKYFQKGVDYLTSHIPIPVVKDVVKSAAKQLVHKISKNQQLCLIVDTVQWCNKSCLAAENKEGYCHGTKCKCGIKVSY**	antimicrobial peptide opiscorpine3-like precursor
comp20745_c0_seq1	**MMNLSLSGCGFLCIYHVGVASCFREYAPHVLVDKIAGASGGSLAACALICSVSLGETTSDVLRIALQARSRTLGPLHPGFDLNKILYDGLVRLLPEDAHLRCNGRLHISVTRVKDFKNVLLSEFNSKDDLIQALLCSCFIPFYSGIVPPKFCGVAYVDGGLSDNLPVLDDNTITVSPFAGESDICPEDTSFNILQFNMSNTSIAVSAGNLYRFVSTLFPPHPEVLSQICQQGFDDALKFLQRNNIISCTRCLAVQSSFGIAESGIVDTKELENDHPDDDCIDCRYRRQRALLDSLPEAVVKAIEDCCEQMNKGVINWLFRHNPVKILPFFALPYVLPIDITIVVFAKIWETLPYIQREMKSSLSEMFAFVKNLVYTFDKGSQYSAKFSCQLAIREFDYANKERKLSTGSAVTNVQPSSSAEENTAAKRQFKKRMSYAGCANISQRLPMRRKSMVETSAPERVIKNMKFGFTVDLSETSMTSENRKKKVIDAFQSLKENNANVFDIANKVFELEKDYIEYIEPQKSDFVEALEVTNTNEAVMAFFYKEGKKVKVTEIFNLTEENAGIAMTDDEKEANTNLQWDSGWDMVSSSLSDYESAVEDDQDCFPVDDHLQPCTSSFGTADAYSMDIHGHELRRARKKSVISRLPFPSIDK**	patatin phospholipase
comp74529_c0_seq1	**GESIASLLSIMEVLTGHIAPYKTVSAKALEDCHVLRLRVEAFKVLLERDPESLLRTTQIIMVRLQRVTFTALHHYLGLSTQLIRTHAKKGIHTMSPKASPSRPSSRRISQ**	patatin phospholipase (partial)
comp122501_c0_seq1	**KIAGASGGSLAACALICSVSLGETTSDVLRIALQARSRTLGPLHPGFDLNKILYDGLVRLLPEDAHLRCNGRLHISV**	patatin phospholipase (partial)
comp1249_c0_seq1	**MGALTLLAFALLTCVAAELNPDNELYVNFEPLPDQTDAWPMARAVRMQFTRRSENGREFRSFQGCQVLESLNHIAREASRTPEQAIQKISKEEMRFFEGRCQRMGDAERTIWGTKWCGAGNTAKKYSDLGIFNNLDACCRDHDHCDNIPAGKTKYGLKNNGTFTMMNCKCEEAFKKCLDAITGKWSSAAIKAFKAVYFEIYGNGCYNVKCATGRSSRGGECPNGVATYTGETGLGAVIINS**	phospholipase a2
comp5045_c0_seq1	**MKTAGVIILLSSLMAAECGIFDVVDKIVPIITTFYKEKDGHRMVETIEINTYIDGKKMVDCYLYGHVYIIDKMMEMVPSDIVKEVGKKEMSKLVNKCSDLLVANIRKGIFNIVKTPFDFARKIFKLLLIFPGTKWCGAGDVADDYNDLGLFEETDKCCRTHDHCNDSIVGFETKYGLKNKDFYTKSSCNCDLPFHKCLYEKEAIHSDAVGHLFFNILQTQCFKEDYPIVKCLKKWGIPLIRETCQEYELDCGGRKKHQFFDAKMYKGKKESPLLKKLLSH**	phospholipase a2
comp9366_c0_seq1	**MKVLPVIVLATLSIAEGGIFDAVGDVLPITTTFYRETDGHRMVETIEVNTYLNGKKTVDCYMYGDSYIIDQMIKLIPTSLTKEVDKEEMSDLVNQCSELLLNQLSSGVFHSIKSPFDSIRKAFKSLLIFPGTKWCGAGDVANSEDDLGRAKDTDICCKIHDHCNDSIAGFETKYGLKNKDFYTKSNCECDRHFHSCLQNGGNLPSDLVGKVFFNVLQTQCFEEDYPQIRCLEKSGIPLIRESCQEYELDYNGTKKYQFFDAKAYVSRGNAWILDKLEL**	phospholipase a2
comp18666_c0_seq1	**MLLLTAFLLSLVQPLPSAVIQLPHENKLTGYYQREKRPHMLIIGQTGKVMHCHRYDDKNEADRVLAALKLEDIQRVTPQLMEKLINFCTEEESIKHPKEQVKKILIYPGTKWCGMGNSAANESELGREKEADSCCRDHDHCDDSIPAFSIKYNLTNYSPFTKSNCSCDRQFHLCLVKAGTEAAGIISGLYFDLLKMECFRRTNYCSSNEVCTETWQWKLSSSYF**	phospholipase a2
comp11436_c0_seq1	**MFFNLFGDLEDLEAILNKKTQKRVLGSGHCSGLIKLLPRYKDLYVAQDSWNTYNSMLRILKKYVMPVRSSTTTGSKMIAGNTMTFSSYPGTVFSGDDFYVISSRLIALETTIGNSNSSLWKYIVPNKIVLEWIRNIVANRLSRTGEGWTYLFSLYNSGTYNNQWMVVDYNRFFPGKPPKKGALWVLEQLPGHIERKDQTDHLLQETYWPSYNSPFYPDIFNLSGTLDMVKKYGDWFTYDKTPRALIFKRDHKKVHDLASMMKLMKYNDFKNDVFSRCNCTPPYSAENAIAARCDLNPPNGTYPFPSLGHRSHGAIDMKLTSYFMHMRYQFVAYGGPTYDQQPPFQWSKSDFSGEKHEGHPDLWKFKPIVHKWIGDPES**	phospholipase b
comp50294_c0_seq1	**WLELKEKDEVNGEKKNAVSKTPSIKFNKFKSSNKGNASNETRYSPITPERRIKSLNDSRAEEVKSPKRQVHSEGEMISDDDAKENTSTSAISRFRKISLRKLKVWR**	phospholipase c epsilon
comp73461_c0_seq1	**NGDIVTGDSDGVARIFTCHSDLQASPEEQLLLEEEISKTALTAEEIGDLKLNSIQGKELLYEPGSRDGQVIIVREGSVVTAHQWSAAEGKWLKVGDVVGASGSTQNTSGKTLYEGEEYDYVFTVEIEEGKSLKLPYNITEDPWLAAQQFIHKHNLSQLFLEQTANFIINNTRGMTFEQQSPTGSDPFTGGSRYIPGNAQ**	phospholipase a-2-activating protein
comp74269_c0_seq1	**DYVFTVEIEEGKSLKLPYNITEDPWLAAQQFIHKHNLSQLFLEQTANFIINNTRGMTFEQQSPTGSDPFTGGSRYIPGNAQTPTSPPTSADSFSSNKSYFPVGNGVVKEEKTETAAFGDGITNTFFPQTEYVTFDVANIEGITAKLKEFNKKVTPEQQLTDEEIT**	phospholipase a-2-activating protein
comp91550_c0_seq1	**FPGLDILRLSVRRSTVNKRVCETAGVQLTDHLLSFLSSDGQVANKMLSLRTFCNLFSHSAGKKLLTNQVERVLSTASNCHSSDN**	phospholipase a-2-activating protein
comp101154_c0_seq1	**KVLASYWKHTRPIMLKFLQSLQALNSTDISIETKLFVVPIYSPTQKRIPYARVNHNKYMVTDESAYIGTSNWSGDYFISTGGIGFILENTE**	phospholipase d3
comp108928_c0_seq1	**AGVQLTDHLLSFLSSDGQVANKMLSLRTFCNLFSHSAGKKLLTNQVERVLSTASNCHSSDNRNVQISLATLYLNYVITFCHSKLEVKSQCVPKILSALKQKTDSEAQFRLLVALGTFIWHDNEAVAFTKTLDLPALVEKMSEIKDPPKVGQCAEYLLSVLFDVN**	phospholipase a-2-activating protein
comp116679_c0_seq1	**YNMGSFRNVITLKDLKAFLPKANCKLPTNRLKEHFQDVDLRRAGEIGFDGFATLYHNIVHDEQLLKDLGQYSADGQKITLQEFQNFLIQEQKDLMAEDERTVSEFMRD**	phospholipase c gamma
comp118708_c0_seq1	**IKFHEVIKTIKEHAFVTSEYPVILSIENHCTLPQQRKMATAFIEVFGDMLLSQSVEREGTKMPSPQQLR**	phospholipase c gamma
comp140532_c0_seq1	**HQQGEVLKKLMELAQTAQMKQLEMKFERENKEMKGKQAKISVETAREVSGDKTLRNKAERERRLREKNSNNTKKFIEERKTAALKQ**	phospholipase c beta
comp147891_c0_seq1	**FMRDYLQDPSRDTQEPHFTVFEFLDFLFSKQNEVWDKRHDQVNQDLTRPLTHYWIASSHNTYLTGDQ**	phospholipase c gamma
comp849_c0_seq1	MNAKLIYLLLVVTTMMLMFDTTQA **GDIKCSSTKECFRPCEEIGGCSNAKCINGKCRCYGCI** *G*	alpha-KTx potassium-channel inhibitor kcug2 precursor
comp849_c0_seq3&8	MNAKLIYLLLVVTTMMLMFDTTQVGG **IRCSGTPECYEPCAKKTGCYSAKCINGRCKCYGCSR**	alpha-KTx potassium channel blocker precursor
comp849_c0_seq4&9	MNAKLIYLLLVVTTMTLMFDTAQA **VDIMCSGPKQCYGPCKKETGCPNAKCMNRRCKCYGCS*G***	alpha-KTx potassium channel blocker precursor
comp849_c0_seq10	LTFDTTQA **VNIRCSGPKQCFDPCKKETGCSRAKCMNGKCRCNGCR*G***	alpha-KTx potassium channel blocker precursor
comp1069_c0_seq1	MNAKLIYLLLVVTTMMLTFDTTQA **GDIKCSGTRQCWGPCKKQTTCTNSKCMNGKCKCYGCV*G***	alpha-KTx potassium channel blocker precursor
comp1069_c0_seq4	MNAKLIYLLLVVTTMTLMFDTAQA **VDIMCSGPKQCYGPCKKETGCPNAKCMNRRCKCYGCS*R***	alpha-KTx potassium channel blocker precursor
comp2965_c0_seq1	MNAKLICLLLVVTTMILMFDTTQVRA**VKCFHNKMCLIPCGMKTGCPEGICVKGRCKCSGC** *NGKEKKCFFQS*	alpha-KTx potassium channel blocker precursor
comp14979_c0_seq1	MGTIIHMAILISLLLLGEREA **KDDYPRNFEGNCYRCKYLEIGYCDAICKMHKAETGYCSRSNLFCYCTGIEDEYVSARNFLYQQNLKINNEELKDFDGNTL**	Sodium-Toxin scx1_titse precursor
comp17675_c0_seq1	MGTIIHMAILISLLLLGEREA **KDDYPRNFEGNCYRCKYLEIGYCDAICKMHKAETGYCSRSNLFCYCTGIEDEYVSARNFLYQQNLKVNNEELKDFDGNTL**	Sodium-Toxin scx4_titse precursor
comp644_c0_seq1	**MRVPLALLATVCFVLGKPQDDTGYGRPAVPPKPIGDYDEDVGGPAKEPDDYDNTGGGKVPVTADYDPLPNPGLKPAPNDPDDYADDKPKGSDAGIDGDKNCICVPYYQCKEGEIVTDGTGILDARKKPPPETELPLDSKFEPPFCGSFHVCCKAPLEPTPGTIYEPKCGVRNPGGIYSRILAPDKKGEANFGEWPWQAAILKVERKINIFQCGGVLIDQRHVLTVAHCICHYRELNQYPLKVRLGEYDTQKTDEFLAHDDFEVEKIICHRDFRNNSLWNDIGILRLDREVAFSPHIDTICLPTYQNVFEGQSCVVTGWGKDAYKGGSYSNIMKEVNIPVINNTKCESLLRKTRLGKYFKLHENFICAGGEEGLDSCKGDGGGPLVCYRKDGTYALAGLVSWGIDCGQPGVPGVYVRVQKFLPWITEQTGFPLEHYFPKKYE**	Putative conserved domains of serine proteinase stubble
comp1431_c0_seq1	**MKMYFIFFVFANILLQAHFLPAKEEGRIFRGREVNDGEFPWMVFIKLTDELNCSGFLISNYYVVTAAHCMIRSATDMRGVIGSVDREQDNMLEFETYFIHPEYNREKNFYADVALLKLKTPIQFTSLIQPICIGKKSSFIQKDNEVLQMGWGRDRNDSTIVSKKLKVTDVGKLMSQDYCNSFFEELNGTSIGKICVKNTEIEGVCEGDSGGPLVYNDPEDGYVVIGLLSFGFYVNCTVTNEYPEIYTNVAYYSDWITENVKSPCVIE**	Putative conserved domains of serine proteases 1 2-like
comp1589_c0_seq1	**MKMYFIFFVFANILLQAHFLPAKEEGRIFRGREVNDGEFPWMVFIKLTDELNCSGFLISNYYVVTAAHCMIRSATDMRGVIGSVDREQDNMLEFETYFIHPEYNRRKADVALLKLKTPIVFTNLIKPICIGKKSSFIQKDNEVLQMGWGRDRNDSTIVSKKLKVTDVGKLMSQDYCNSFFEELNGTSIGKICVKNTEIEGVCEGDSGGPLVYNDPEDGYVVIGLLSFGFYVNCTVTNEYPEIYTNVAYYSDWITENVKSPCVIE**	Putative conserved domains of serine proteases 1 2-like
comp4148_c0_seq1	**MSESELNVDSIISRLLEVRGCRPGKTVQLTEAEVRGLCLKSREIFLSQPILLELEAPLKICGDIHGQYTDLLRLFEYGGFPPEANYLFLGDYVDRGKQSLETICLLLAYKIKYPENFFLLRGNHECASINRIYGFYDECKRRYNIKLWKTFTDCFNCLPIAAIIDEKIFCCHGGLSPDLQSMEQIRRIMRPTDVPDTGLLCDLLWSDPDKDVQGWGENDRGVSFTFGADVVSKFLNRHDLDLICRAHQVVEDGYEFFAKRQLVTLFSAPNYCGEFDNAGGMMSVDETLMCSFQILKPSEKKAKYQYGGLNSGRPVTPPRGPVKKK**	Putative conserved domains of serine threonine-protein phosphatase pp1-beta catalytic subunit
comp10302_c0_seq1	**DSDDSSVCTALEYTSQDLAVDCSTSHLLDLTHSNNLNCCAKQLCVPQEADFPKDEEDVGDDPPKHNNQNEITALDMSLQRSKGNNCEDMMDSAEEITYDTFTNMDHTLKLYIEMQLFNANEELEASIEAVLVSHSTTIKNKGLLLLSTKRLHFMKLAENLNEDPKNNVTPLEAVELHLLHTVQIFTGNQGASFIWGSEKNPSHCKGCYTCLFRDADYCCTFITYFIDFMKHKVSLLPAINASSEFNLSQVKQDVLFAGAQNSVSCKISTEILAFIIVDKCQLESPEKEYGIAALTLTSTDICLTDIIFTKRSSKPSEFFKPVESYMLIAKQKVTNLVSVHPHFDTCKIGLHFLDEDSDKELLWMIVLKTKRMLYLLVNTLKEPWQDNFGIEMKTETPADCECNLY**	Putative conserved domains of serine threonine-protein kinase 11-interacting
comp17155_c0_seq1	**MDLIQHELVDIRKHCESHIPGCKLITCVQAMVRVDIVRTEHKQLTACIQFPKKYPNETLLIELKSKYFSEKLLDGLTKVCDEKCRKHLGKPQVLELLKFIQNFIAENPLCVCSEEIAAIKKRLSTEKDELRLKQKTSSLALRIYQDLYFLVVKIQVPDNYPLEQIKIEDKDSNFPELFKRHFTSQAVEIARQCVQPPLKRKPKDPPFEPKPSLWPVVGFLIDQVKRVPLEDCPLCKTRCLPSDPKDIITDDKDDTFAERIYCGHVFHNGCLNKYMKMPPFQGKTCPKCEQRIYHEKWKATPQLVEARWAHEEAKKRELGEVVEFLQ**	Putative conserved domains of serine threonine-protein kinase mrck beta-like
comp22520_c0_seq1	**GEFDNAGAMMSVDETLMCSFQILKPTDKKKYPYGANRPVTPSQITKKNSKK**	Putative conserved domains of serine threonine-protein phosphatase pp1-beta
comp30034_c0_seq1	**LFPNLQVINVSHNRLANAIGLKYLTKVSAVNLGYNNLSKIPTFSDESFKFLQELSLRNNNLEDLKGLESLRNLHDLDLSYNCLSEHSVLFPIHSLPYLQLLKLVGNPLAIQRLHRILTAKHLHPNVLVVGMKLDGRQLTKTEIGKVTQVRAINTYYNNRNGERIQTNSLLEEANIIRNVTVSSLSHCASFSSLDRHTVASESSLKNDTESITSTKSDFVKKGKSKSRKKKEIIIEDCGSTQQYFLTQNFPEKECKTKELKATLAARRENLGQQWLVSTYSTSLPSESILRNMTTMPESSREISVHNDTSGPSFNNTIESSPSTGITAILVGTKEVENYSEEVDNVEKKVKKKISVASDGIEVICSEHIHQDKIADNDFSMCKSCMQLDQEDNNEIDFQENRNAYNIKYGIAGEDENIESNVFLVEKKVSQDETAIIFVSIGEKYLNEEDTLTNDLLDSLDLSVLDTVEMLGKNTIKLDFKIFKSSQRERTYCTESEVAAEEMLNLLLPHADARSLRNIIKNALRCLKCDVQFTKNIVEERFISDIAHPLAKYILRDNNLSEIEFEGKDICPVCESLMIVEDNDCPVTTSLLKDANYSPAIISGYLFVPQNTSSSVSVDFTSNLLLTFKHPPKVKQDTVSSLSNNATVNAASHGEA**	Putative conserved domains of serine threonine kinase 11-interacting protein
comp35829_c0_seq1	**YQYSWNNFETLKFKQNFAFSRIGNYIASKRNKTRWIWISAIVASGICAFSYIRNCSRVYCNQEKTYQREIEKCRDILRRRKDEVGAPGIIIGVSINGRTVWQEGLGYADVENRVPCTENTVMRIASISKSLTMAAVAKLWEDGKLDLDKPVKTYVPYFPEKTFEGKKVDITCRHLVSHLSGIRHYDKKNLEKDSEKLDSKIKKEENNKEKDEISKRNENEQKSEFDLKEYLIKEKYESVKASLDLFKDDPLVHKPGTKFLYTTHGWTLLSAVVEALAEKPFATFMKQLFKDFGMTNTYLDTNDAIIYHRARFYLRDKKGHLQNTPYVDNSYKWAGGGFLSTVGDLLRFGNIMLYSLQHKAQDLEKNINQSNELQKAEDSRCVETNTSSPTKELCYNTSADNTEITLCALPGYLKDETVKAIWLPVENATPNWEGGIRYGMGWGIVSQKQKCGYCRE**	Putative conserved domains of serine beta-lactamase-like protein mitochondrial
comp59215_c0_seq1	**NTAEAEVKKIQDEVNTLKKKNCELESELTKFRQQQREFLTGKHEFEPFQDEKYQNKEYEKMMRHLKAEKEDLHRELTEVQEKLKLQSKELKDALCQRKLAMTEYAEVSDKLSELRAQKQKLSRQVRDKEEELENALQKIDTFRQDLRKADKLRRELEARIEDFKSDSLKERRMKERSEEYSRHLEEEMESLKQRHVGWGANPSHLESQEITRLKLEIESLEVQQKEMLTQQQSRFSAEMSNLLDQLQDAESIKESLDEEIISLKEKAEKARSESSIEHQEVINELKRTQEREKQLLQEDNRKLNLEIERMTELINKQQDDKRRLEEDLIQIREKKESIIQWEAQISEIIQWVSDEKDARG**	Putative conserved domains of serine threonine-protein kinase mrck beta
comp59760_c0_seq1	**ILISELKIIHIEFLNFNIPYSFNCVFDGLLIFNGKTTESDILLHACGQSFPKNVTSTGPFLHLIFYIDGIWNYGGFALRFKQISPREPCGEHQITCRNYNCVNRTLICDGADDCRDGTDEECGYKRQRLICGRPKIKPEFIDDRIVGGTKAVPGSWPWQASLRVPSAEPFGHVCGGSLINEQWILTAAHCFRDIQKESWTVHLGKYNKNKRDHTEQLRYIKRLFIHPQYLEMIKKE**	Putative conserved domains of serine protease
comp92380_c0_seq1	**LKVTHCMETVQGGIDLSMFKSLLMLELKKTPIHLLLGLNELCSQLETLVCSCSISSLHELVGNKSLEWTVLKQLNLSHNYLEDLQEDTK**	Putative conserved domains of serine threonine-protein kinase 11-interacting protein
comp112232_c0_seq1	**LELKKTPIHLLLGLNELCSQLETLVCSCSISSLHELVGNKSLEWTVLKQLNLSHNYLEDLQEDTKLFPNLQVINV**	Putative conserved domains of serine threonine-protein kinase 11-interacting protein
comp130276_c0_seq1	**MDRVKFPSLPDSEDTEWTYAMRRDMQEIIPGLFLGPYS**	Putative conserved domains of serine threonine tyrosine-interacting protein
comp6164_c0_seq1	MQFKRLLVALTLICIVSC **EEKRDSSGRSCSVTGICMKSCARFLHQPANHKKCLPDGVCCTLIY**	toxin-like toxin tx277
comp6514_c0_seq1	MQFKRLLVALTLICIVSC **EEKRDSSGRSCSATGICMKSCARFLHQPANHKKCLPDGVCCTLIY**	toxin-like toxin tx277
comp395_c0_seq1	MLKTVIFCIAVLASVCTG **EENSEEGRTFPLLFSADGRNSLGCWITYSFSYQPTADIDTKIAAQNTLCECMKKGLVPK**	toxin-like toxin tx707
comp493_c0_seq1	MKATVLLIAVFILFSVFG **DMGYCEFCDTPHCTRVCYDHCVRLNKHYKTCCMTNINDRIRMECLCEDKTGIKPYYPNNI**	toxin-like protein 10 precursor
comp26529_c0_seq1	ALFILFSVFC**QMGYCQSSNSRRCYRSCLDYCTRLNQVYKSCNVSNSNGVKHLRCDCES**	toxin-like protein 10 precursor
comp3375_c0_seq1	**TCVLSHPAFCVDDSGVRYKPGDVWYDDEKCEKLRCSGAEASLKIIGAGCGTIHVVGCETVRGSGHYPNCCPRPKC**	toxin-like protein 14 precursor (partial)
comp4212_c0_seq1	**DIVKVVCVDKSGVEHKPGEVWYDDERCQKLSCDRIKWNLEIVGMGCAPAVSAHCNPVRCSGHYPNCCLHC**	toxin-like protein 14 precursor (partial)
comp4212_c0_seq2	MNTYNSRFYIFSLAIALVILEGTEG **YMFRIAQDPGAVVCVDKSGVEHKPGEVWYDDERCQKLSCDRIKWNLEIVGMGCAPAVSAHCNPVRCSGHYPNCCLHC**	toxin-like protein 14 precursor
comp79719_c0_seq1	MNTCNARFYIFSLAIALMILKDAEG **YIYRIPQKQGAVSCVDDSGVKFNPGNVWYDDEKCERMSCDGAVGNLEIV**	toxin-like protein 14 precursor
comp299_c0_seq1	MKVACSLVLLVAFTCTVSA **RVVSKKTCKTHTGVILRHGEEWKDPNHCSIYRCTIYDGEAELDGLMCATYQVPRNCKFVRGGGKLYPSCCPTVVCK**	toxin-like tx11_opicy
comp749_c0_seq1	MKASTLVVIFIVIFITISSFSIHDVQA *SGVEKREQ* **KDCLKKLKLCKENKDCCSKSCKRRGTNIEKRCR**	**calcium-channel toxin Contig20-Uy precursor**
comp10032_c0_seq1	MNFSSKISFLLLVTAVVFA *VTGGEVDRLFEQYKESDIER* **DLPPSDEYGTCVRPRKCKPHLKCSKAQTCVDPKKGW**	calcium-channel txs2b_liowa
comp27527_c0_seq1	**AEPAYAEARCIRRGRMCDHNKYGCCNNGPCRCNLFGTNCRCQRRGLFQG**	calcium-channel u8-agatoxin-ao1a-like isoform 1 (partial)
comp104104_c0_seq1	**LNAEKRSCVRRGGPCDNRPNDCCQNSSCRCNLWGTNCRCQRAGLFQRWTGRK**	calcium-channel u8-agatoxin-ao1a-like isoform 1 (partial)
comp1735_c0_seq1	**MSQGIMKQQISVFIFITFAFVSTNGKNICNSKYRSIHPEHSMCKTRNDSCRFTQRIEENAKELLDVHNQIRNSIHHVIGKDYFEGENMKMMQWDHELYLMALKHVLQCSELPDCSLCHQTDRFHVEQNFAVKTFTSVSQSFNGTVERFKMVIMEWAKEILQYNPDIVNRFHSVGLPTNWTNIFRATTYKVGCASVGYHTQIEESFREVYICNYGPALLMENEVIYKPSYRSCTNCNSSSNCSANDNLCRNHFPWKYGHNNSYTFNELLRRGKRKAGSSGKKWCDSKERRYEQDPCTDEYQNITADHSFCKPPNMECECSRNYERYRKLLVDTHNEIRNSVQAYASWHDSTATNMRVMEWDDELYAIAYRYVSQCLEEPDCYLCHQAKSFPVEQNFVAKVLIDTSSRSGKRFADIVKEWATEFRQFSQQDVEYLPEEAAKDQNNHWINIFRAGSWKVGCASISFKNSTGTSSDNRLVKEIYLCNYGPAKLIPGEKVYEIGKSCSNCEIGFSCDEKYPNLCSSSINHAVVTPAFTKENDITDATPAPATITSTTAPVPIMTTSTTPSPTMKTTTITPAPTMTTTTTTTPVPATTTTPTPTPTTTTTTPAPTTTTTTTLSLSTTTTSTTLTPTTTISTIPVPTTTTTTTPVPTTTTTTRAPTITTTPVPTTHAAETTVSSQQSFTDLISNYSASYTTNTPKLPIDLSTAFPSSNKEKASGRSVIWKCTLSFLDELTCRNEQQCYKAWTLSTDEKQPYMEIDIPEDTRSGLLFLENIYIDKPSCFTFAFRKTGLNTLSFLTSILYGIAVRIENNENVIVETSEDFPDWNSLFVDIPWIKVFIQVGIAVKTNGGIGMQHVEIKDFLVYHGPCSAL**	Putative conserved domains of venom allergen 5
comp4029_c0_seq1	MTSVAVITLALWITAIRCFA **SNDTCDERYSRITTDHTMCKSINQNCNFLKRREKVFEERLLRTHNSIRNSIRKYVGRKYHLATNMKVMQWHDELYAMARLHSLQCAEKPDCDLCHQIGDFPVEQNFAVKTFKKSKSARSGGPFRRFQTTIKEWAAELRLYNRDVVKSFRTTAGLPTDWTNILRATTIFVGCASTSFKTDERGTFKEVYVCNYGPANLTEGEEIYKAGKKSCSECEDGIGCDTEFKHLCFPGDVEKEH**	Putative conserved domains of venom allergen
comp4170_c0_seq1	MTSVAVITLALWITAIRCFA **SNDTCDERYSRITTDHTMCKSINQNCNFLKRREKVFEERLLRTHNSIRNSIRKYVGRKYHLATNMKVMQWHDELYAMARLHSLQCAEKPDCDLCHQIGDFPVEQNFAVKTFKKSKSARSGGPFRRFQTTIKEWAAELRLYNRDVVKSFRTTAGLPTDWTNILRATTIFVGCASTSFKTDERGTFKEVYVCNYGPANLTEGEEIYKAGKKSCSECEDGIGCDTEFKHLCSPTEKKTT**	Putative conserved domains of venom allergen
comp4913_c0_seq1	**MKVFDILALALLSWLRWTSSVAQQYLPIVNIGQGALRGRILKTSNGRDFYAFRGIPYATPPVGVFRFKEPNPHAGWGGVLDAIDYRAKCPQIDLQGVQTGDEDCLFINVFTPTLPTFQPSSQRSSVRLVTYPTMVFIHGRTFDSGSSNLYGAERLLDKGVVVVTFNYRLGALGFLSTGDDKASGNWGLLDQRMALGWIQNNILRFGGDPKTVTLFGQGSGAASILIHIISPLSHNLFHRAILQSGSALCDWTIQHNPLSYAKNMATRLGCQTYSTEAIVKCIREQPASSIVREQANMKVFGDFPTGALPVIDKNSASNFLPEHPENLLEYGNFKAVPIIIGVNKDEGAFFYPLLTRKYKEDIQTIPGYFQNTLLPNFLQATTNLNNNLDVISQELIYRYYGGLDLSNPYNILEPFINMSTDAMYVACTERTLQLYSRLNPTSTYMYTFEYKGTNSLANFQPNLSPQQSQQVDGVSNGDELLYLFNMQIDGLRHPSHLDNMISNRILTLWTDFAKLGKAPQYVNYEYPEWRNYQYDDRSYYRIDRSLSLQHNYRTGVKDLWLRKLRELSSSINPTNSPLTQMQGVEPFYRTLAWAMVAICIALLVLIVVLLAILYNQKKSQSFKANHENQSRMSGSTLY**	Putative conserved domains of venom carboxylesterase-6-like
comp16713_c0_seq1	MGLRLFALVVLIASCHC **WPRKRCSEPCEPVPNNCKAGVTNDYEGCCPICAKSEGEECGGMWNAYGVCGVD**	venom protein insulin-like growth factor binding protein-1 (partial)
comp13767_c0_seq1	MKTSIAIVFLFGFIAAAIA **SHKDPYERNCPIGDKDLGNGDEWADERRCVKYKCQVRGPDAALLITRCPSVGIYPPDKCRELPGKGDFPNCCPKLQCD**	toxin-like venom peptide 1a
comp330_c0_seq1	MLKTVIFCIAVLASVCTG **EENSEEGRTFPLLFSADGRNSLGCWITYSFSYQPTADIDTKIAAQNTLCECMKKGLVPKGGTTTQPPSG**	toxin-like venom protein
comp727_c0_seq1	MGKLCWIAILLLGVSLRAMS **LTCNPCGTYECPSPPTNCRAGQVKDVCNCCIVCGKGLNEECGGPWDIAGKCGRGLKCVKRESSFNARGRCQKF**	toxin-like venom protein 302-like
comp727_c0_seq2	MTGKLCWITILLLGVSLSAMS **LRCRPCGSYECRPPPTNCQAGQVKDICNCCIVCGKGLNEECGGPWDIAGKCGRGLKCVKRESSFNARGRCQKF**	toxin-like venom protein 302-like
comp1980_c0_seq1	MNPRHVLLFLTVIVCTSHA **QSNGFCKPNEEYREAGCEVVCERILENNCFRAEKKPGCYCKAGTMRDERGDCISLKECSKRVCTQKNKRLNLSGCFTVCTGPGTSYSGCPFVPNPKCMCEKGYATQNGFYGECIPVSKCQGNRNGE**	Venom protein (spondin-like) venom protein-9
comp2323_c0_seq1	MNPRHVLLFLTVIVCTSHA **QSNGFCKPNEEYREAGCEVVCERILENNCFRAEKKPGCYCKAGTMRDERGDCISLKECSKRVCTQKNKRLNLSGCFTVCTGPGTSYSGCPFVPIPKCICEKGYATQNGFYGECIPVSKCQGNRNGE**	Venom protein (spondin-like) venom protein-9
comp3435_c0_seq1	MAQIFLLVFLLPCLVLG **SDEPAKFISYRNYAYSPLSEGKCKSSNEKLIEDGDTWYREDFCEKVYCFRTGTMGNMIVRGCAPMTPLNPNCTVVQSPGLYPDCCSGNIVCDQHSEPKSDVEMAEIIRSMLESNRK**	toxin-like venom protein-7
comp4735_c0_seq1	MANRFYFITLLLFGVFMRAMT **LKCRMCDRNDCPPSPENCAVGIVKDVCNCCDVCAKNEHETCGGPWDILGRCGEGLKCVKVSEKDFSAKGTCQKA**	toxin-like venom protein 302-like
comp7830_c0_seq1	**MVYVRGCCCWRNTKDGSIACGYFTLLTRLIGAALIIVGLVNLTSFATYIHGSHSYITSLRLLFISQLIDCLVFIVFSAMLIYGTKTDNTVMMFPWIVWMVIEIGSLIVLLILTFIGVTQGMVTAAVVLAVLISMVFLGIDIYTLLCVTSQYRLLHHGPPSYSVIA**	venom PROTEIN
comp13102_c0_seq1	MGLRLFALVVLIASCHC **WPRKRCSEPCEPVPSNCKAGVTNDYEGCCPICAKSEGEECGGMWNAYGVCGVDLVCQTNGQASSEYDLPIGTCVIARRFSSRNIVKRMLRWF**	venom insulin-like venom protein 302-like
comp13814_c0_seq1	MKLYIFFVLFACAVLPSWC **LIHYHGHLCRYNLIDRFCGLNDRKTPIECLQESEQAARVTEIFKACFTSVKEGVAEFDDQVNEVCKLKHDEYAVFKRCFHNGLVLQQRRDEKSYKAFEECIEKSEKREEKACHYGHYGSVSFRFGWFV**	venom protein-5
comp65882_c0_seq1	**MCKCLWSIFWLVVLIFIAYPIGLFFAEIYVLLSPLQGCCEDCCTGVIEFLLKLVQLPLLCAR**	venom protein-2
comp21903_c0_seq1	**MKAVLITIFLVPLIISQTSAKIRQRRQGFEFPSEAESCTTPGNQPGNCISLSRCESLRRTNDFNLLTNSICGFDNDVPRVCCPDGTANPDVKEITTTSGPKTDNLEPVEITTIPIRPIIITSVAPVTSSPARGKPAILPDECGMSTIPLTRVVGGSPSELAAWPWMAAVYFTRTGLRSGTDCGGSLVTSRHVITAAHCVTDNRGNEVRASTLTVRLGEHILNDDNDGASPIDVPVARLVRHENFQRRVFKNDIAILTLQRDVPFNKFIRPICLPYGVFENADLARMRPWAAGWGTTSFDGEFSPRLSHIQISIETNEDCNRAFRTERVPITQEYLCAGVSDGTKDTCKGDSGGPLMLPVDLKFYLIGIVSFGKRCATVGYPGVYTRVTMYLDWIARNLT**	Putative conserved domains of venom serine protease
comp221_c0_seq1	MKVFCIVLVVVAALALGEA **KSIRSIKSNRLVRSIAPVSARLARSAQSMTDITILTAGATGKRNAKPTESDEELDALIGLSMLEELAKEQKRSVAPKVQHKKRQSGASEEAAEAILGLDLLEELANEAKRSIAKAAKKRDLSRRQSASDEEAVQAVLGLAPLDELAGDKKRKVKKSLFKPLKATKSIKRRATKLFFPFM**	venom PROTEIN
comp221_c0_seq2	MKVFCIVLVVVAALALGEA **KSIRSIRSNRLVRSIAPVSARLARSAQTGATGKRNAKPTESDEELDALIGLSMLEELAKEQKRSVAPKVQHKKRQSGASEEAAEAILGLDLLEELANEAKRSIAKAAKKRDLSRRQSASDEEAVQAVLGLAPLDELAGDKKRKVKKSLFKPLKATKSIKRRATKLFFPFM**	venom PROTEIN
comp233_c0_seq1	MKVFCIVLVVVAALALGEA **KSIRSIKSNRLVRSIAPVSARLARSAQSMTDITILTAGATGKRNAKPTESDEELDALIGLSMLEELAKEQKRSVAPKVQHKKRQSGTSEEAAEAILGLDLLEELANEAKRSIAKAAKKRDLSRRQSASDEEAVQAVLGLAPLDELAGDKKRKVKKSLFKPLKATKSIKRRATKLFFPFM**	venom PROTEIN
comp233_c0_seq2	MKVFCIVLVVVAALALGEA **KSIRSIKSNRLVRSIAPVSARLARSAQTGATGKRNAKPTESDEELDALIGLSMLEELAKEQKRSVAPKVQHKKRQSGTSEEAAEAILGLDLLEELANEAKRSIAKAAKKRDLSRRQSASDEEAVQAVLGLAPLDELAGDKKRKVKKSLFKPLKATKSIKRRATKLFFPFM**	venom PROTEIN
comp34369_c0_seq1	**MTVGNCLWGFFWFLVLLFIGYPVAGFCAGWYVLICPFQACVDGCAPIIDFLLKATQLPLTCAQNMMSGKPFC**	toxin-like peptide-6
comp13137_c0_seq1	MFRLVLLCTFVVSIYS **LSCPCWEVEEDCGPPPTDCALGLTTDVCGCCPVCFKVQGEICGGPWNVNGECGEGLYCRKEHVEEAFDQQEGVCEPKK**	vp302_lycmc precursor
comp71520_c0_seq1	MFRLVLLCTLVAGIYS **LTCPCHYYENRTKDCEPLRKVCPLGVTKDACGCCDVCFKVEGEICGGP**	vp302_lycmc precursor (partial)

In bold: Mature peptide; Underlined: Signal peptide; italics: precursor and amino acid with postranslational modification (amidation); Names of Seq. Description (BLAST), names in bold: sequences reported in [41].

Expression level of transcripts was assessed using Tophat/Cufflinks suite. The most abundant transcripts were those giving hits with venom toxins, hypothetical proteins, antimicrobial peptides, and α-KTxs. Then enzymes, such as: NADH-dehydrogenases, phospholipases, sulfotransferases, elastases and hyalorunidases, which were highly expressed in the venom. The less abundant transcripts were those having hits with sodium-channel specific peptides and cytochrome oxidase I (COI). In S1 Table the FPKM for venom related compounds and housekeeping genes is shown.

### Functional annotation

Unigene annotations provide functional information, including protein sequence similarities and gene ontology (GO) information. Only 31,807 unigenes had hits with the searched databases and therefore only those were annotated. The fact that only 51% of the total number of unigenes had significant hits suggests that many scorpion-specific genes still remains undiscovered. This data agrees with the annotated sequences of other scorpion transcriptomes for example: the *C*. *noxius* transcriptome [16].

**Fig 1:**
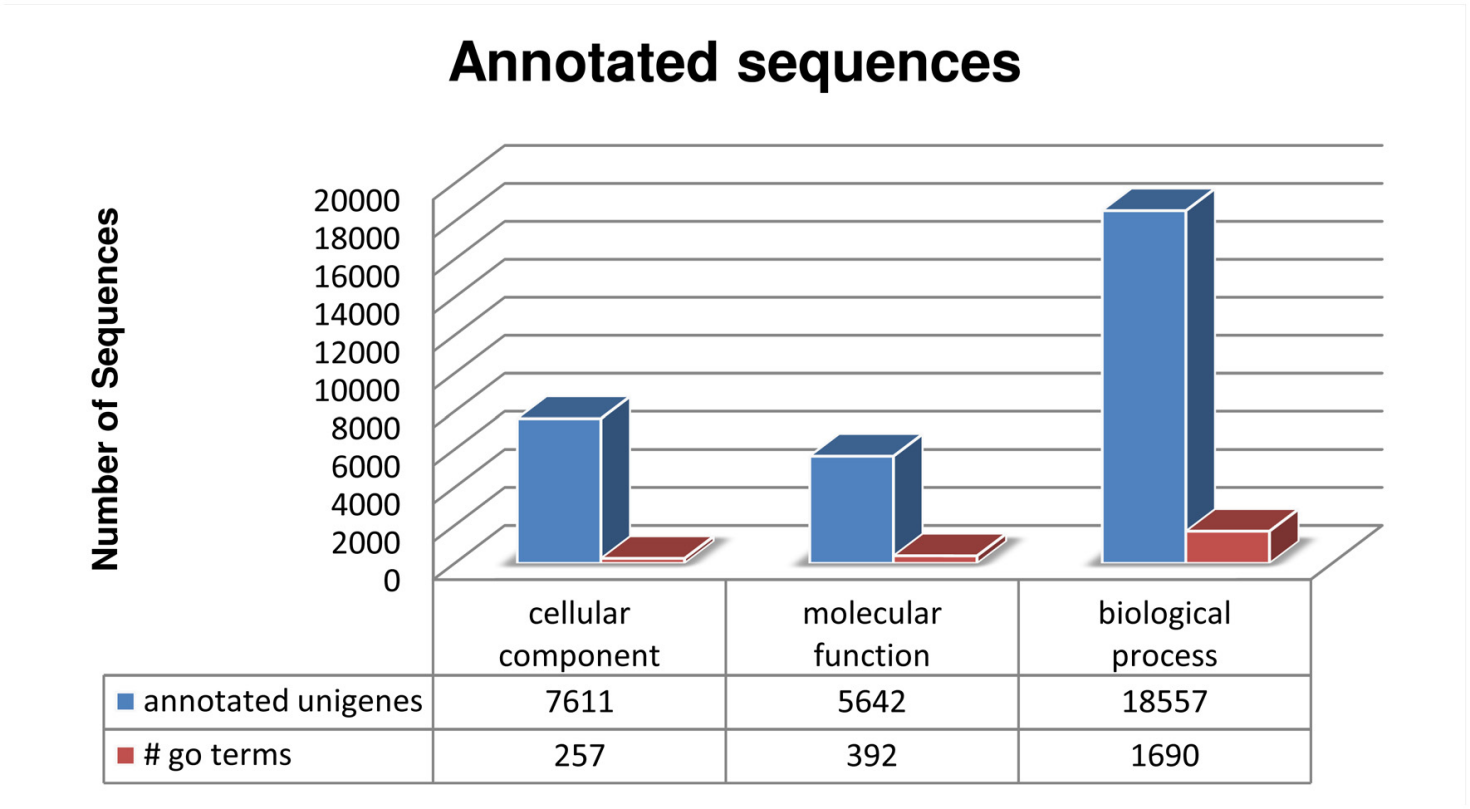
Statistics of Go term annotation of unigenes found in the transcriptome of the venom gland of *Urodacus yaschenkoi* scorpion. The three Go terms domains are plotted with the number of annotated unigenes and also, the variety within each domain is showed (different categories of Go term per domain).

**Fig 2:**
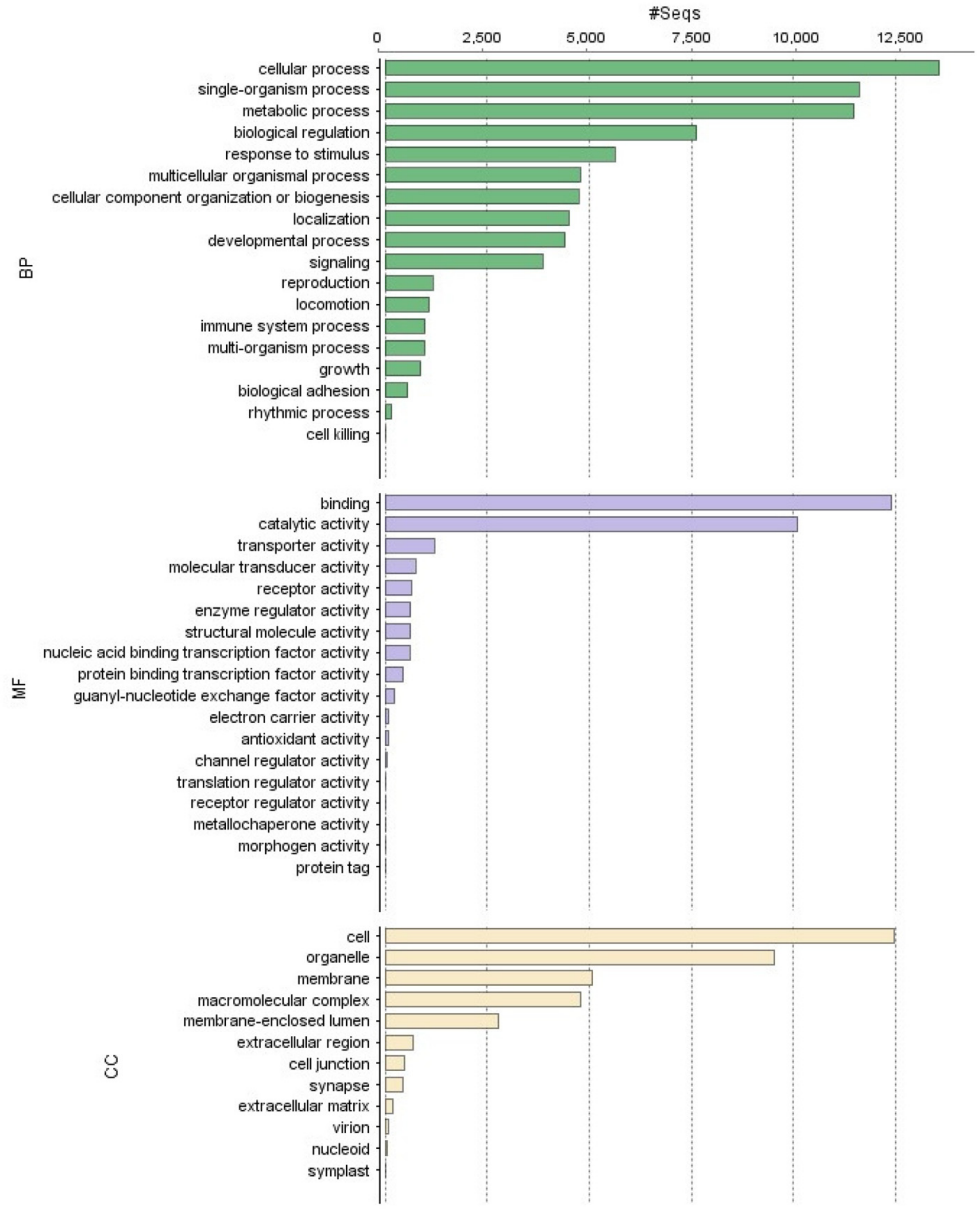
Most abundant categories within every GO-term found in the *Urodacus yaschenkoi* whole transcriptome dataset. The biological process (BP) category was the most abundant, followed by cellular component (CC) and the molecular function (MF) was the least abundant.

The GO database comprised three ontology domains: molecular function (MF), cellular component (CC) and biological processes (BP). For the first Go term, 5,642 unigenes were matched with 392 GO terms. For the cellular component term, 7611 sequences were annotated with 257 Go-terms and for the biological processes component 18,554 sequences were annotated with 1690 Go-terms (Fig 1), making the BP the most abundant and diverse term. In addition, a graph showing the most abundant Go-term categories per domain is shown in Fig 2. Supplemental data show graphs for the sub-dataset containing only toxins and venom related components: most abundant Go-terms, pie charts with the most abundant Go term per domain and enzyme distribution (Fig B, Fig C (A-C) and Fig D in S1 file, respectively). Fig E in S1 file of supplemental data shows the most abundant family of enzymes found in the whole transcriptome.

### Scorpion toxins and venom components identified in the venom gland transcriptome of *U. yaschenkoi*

**Fig 3:**
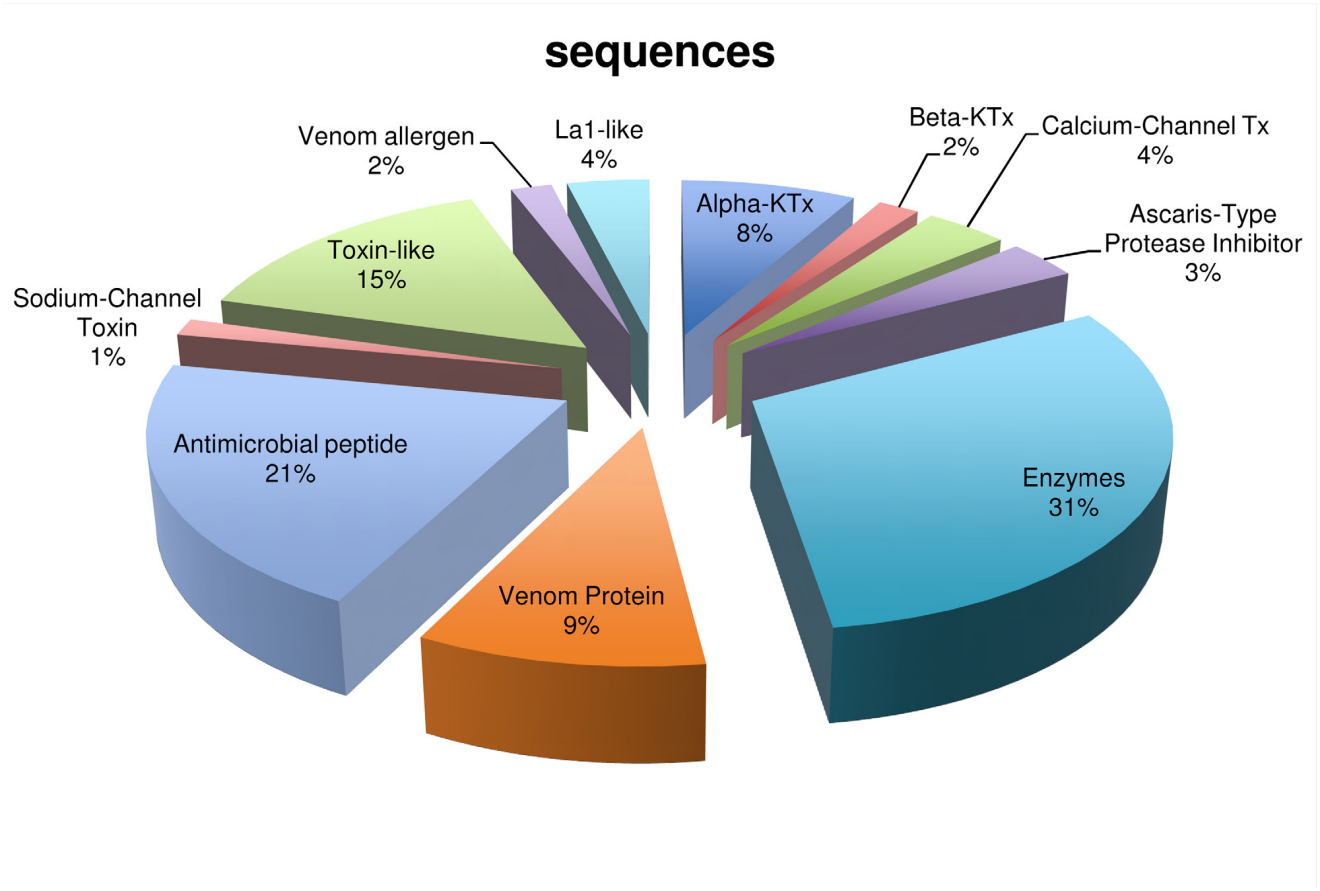
Subfamilies of scorpion toxins and enzymes found in the whole transcriptome of the venom gland of *U*. *yaschenkoi*. A total of 62,505 unigenes were searched against the NCBI-nr database, only 51% had an identity against the databases; from those 3900 were related to venom components and housekeeping genes and 210 sequences codify toxins and enzymes in scorpion venom were identified. The diagram shows the relative proportion expressed as percentages, of each subfamily of scorpion toxins found in the analysis of the transcripts from *U*. *yaschenkoi* venom gland transcriptome.

From the 62,505 unigenes only 51% had significant hits against the searched databases. From those sequences, only 3,900 had sequences similar to toxins, venom related components (such as hyaluronidases, phospholipase, and other enzymes) and housekeeping genes (for example, heat shock protein, β-actin, RNA binding protein). These sequences were further analyzed as described in Material and Methods. Eleven subfamilies of scorpion toxins were identified (Fig 3) and 210 delimited sequences code for 111 unique amino acid sequences are shown in Table 2.

The following lines describe each one of these subfamilies of putative proteins/peptides identified in this transcriptomic analysis, starting with the most abundant components found.

#### Enzymes

It is well known that scorpion venoms consist of a heterogeneous mixture of 100 to 700 different components, among which are: inorganic salts, lipids, nucleotides, free amino acids, mucopolysaccharides, peptides and proteins. Among the proteins, enzymes are the most abundant [67].

From the 210 identified and analyzed nucleotide sequences of *U*. *yaschenkoi* (Fig 3 and Table 2), 31% correspond to enzymes, including hyaluronidases, glucosaminidases, phospholipases, serine proteinases, phosphatases and kinases.

For example, component label comp120806_c0_seq1 in Table 2 corresponds to a partial sequence of a putative hyaluronidase-1 (isoform 1) showing 93.58% identity with hyaluronidase Uro-1 previously identified in the transcriptome of *U*. *manicatus* [49]. In addition, the sequences comp7041_c0_seq2 and comp7071_c0_seq2 code for putative hyaluronidase-3 isoform x3 (Table 2).

The sequence comp374_c0_seq1 (Table 2) from *U*. *yaschenkoi* shows similarity with a chymotrypsin-like protease-1 from *Mesobuthus eupeus*.

However, the most abundant enzymes found correspond to proteins with phopholipase activity (Table 2), which is in agreement with literature data found in venoms of non-Buthidae scorpions, such as phospholipin and a heterodimeric phospholipase A2 isolated from the venom of *Pandinus imperator* [68] and sequences found in another transcriptomic study conducted with the scorpion *P*. *cavimanus* [40], which are assumed to be phospholipases.

#### Antimicrobial peptides

Linear peptides containing no disulfide bridges (NDBPs) have been abundantly found in the venoms of scorpions of the families non-Buthidae, contrary to what is reported for scorpions of the family Buthidae. The latter contains a substantial amount of peptides tightly joined by disulfide bridges. Among the NDBPs found are peptides displaying antimicrobial activity (AMPs). Until now, at least 40 AMPs from scorpions are described in the literature [69]. These were subdivided in three main categories: long chain peptides (over 35 amino acids long), medium length peptides (20–35) and short chain peptides (less than 20 residues) [70]. However, venom from Buthidae and non-Buthidae scorpions shows the presence of peptides with antimicrobial activity that do contain disulfide bridges (DBPs), such as the defensins and scorpines.

**Fig 4:**
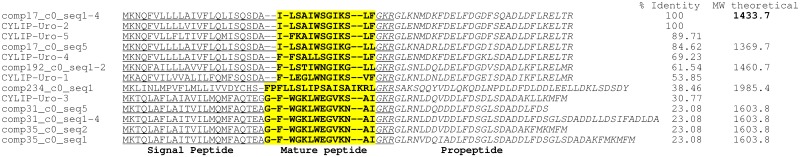
Sequence comparison of putative antimicrobial peptides from *U*. *yaschenkoi* and *U*. *manicatus*. Multiple alignment of sequences obtained from the transcriptome of *U*. *yaschenkoi* that codify antimicrobial peptides from the subfamily NDBP-5. These sequences are compared with CYLIP-Uro-1 (GenBank: GALI01000003.1), CYLIP-Uro-2 (GenBank: GALI01000004.1), CYLIP-Uro-3 (GenBank: GALI01000005.1), CYLIP-Uro-4 (GenBank: GALI01000006.1) and CYLIP-Uro-5 (GenBank: GALI01000007.1) from *U*. *manicatus* [49]. The predicted signal peptide is underlined; the mature peptide is in bold and highlighted in yellow, the conserved proteolytic site GKR is in italics and underlined and the propetide in italics. The hyphen (-) in the name of *U*. *yaschenkoi* sequences indicates that the amino acid sequence was found within different nucleotide sequences (transcripts), for example: comp17_c0_seq1-4 means that four different nucleotide sequences codify the same peptide. The percentage of identity of the mature peptide is indicated at the right (% Identity) with respect to the peptide encoded by comp17_c0_seq1-4; additionally, at the far right, the theoretical molecular weights of the antimicrobial peptides for *U*. *yaschenkoi* are shown. The theoretical molecular weight of the peptide encoded by the sequences comp17_c0_seq1-4 (in bold) has a perfect match with the *U*. *yaschenkoi* proteome previously reported by [41] at the retention time 13.88. Note: several sequences of transcripts found in the *U*. *yaschenkoi* transcriptome codify the same precursors, such as: comp17_c0_seq1-4 and comp18_c0_seq1-2 (not shown); comp17_c0_seq5 and comp18_c0_seq3-4 (not shown); comp192_c0_seq1-2, comp192_c0_seq4-5 (not shown), comp192_c0_seq7-9 (not shown) and comp196_c0_seq1-7 (not shown) codify the same precursor.

In this work sequences that code for antimicrobial-like peptides were the second most abundant group having 43 sequences (21%). These sequences codify different subfamilies of putative antimicrobial peptides, such as short antimicrobial peptides (IsCT-like) from the subfamily of non-disulfide bridges peptides-5 (NDBP-5), defensins and long-chain scorpine-like peptides, being the first ones the most abundant within this category of AMPs. The short chain AMPs of the IsCT type were initially isolated from the venom of the scorpion *Opisthacanthus madagascariensis* [71]. The IscT peptides are derived from large precursors, characterized for having a signal peptide of 23–24 amino acids, a mature amidated peptide with less than 20 amino acids long and a propeptide containing from 31 to 46 amino acid residues. In addition they show a conserved sequence of three amino acids of the type GRR and GKR, which marks the end of the mature peptide and the beginning of the propeptide (see Fig 4). The identified NDBP-5 sequences found in this work were mostly 13 amino acid long peptides, but seven sequences were 14 residues long and only one sequence was 18 residues long. All of them were manually analyzed and the signal peptide, the posttranslational modification motif (GKR) and the propeptide were identified and delimited. Fig 4 shows the alignment of NBDP-5 sequences found in this transcriptomic analysis. Interestingly, peptide GFWGKLWEGVKNAI was codified by 7 different genes showing different propeptides, suggesting a wide array of mechanisms for toxins production. Overall, five different antimicrobial peptides with unique sequence were also found. One of these transcripts is the peptide ILSAIWSGIKSLF which was previously found in a shotgun cDNA library previously reported by our group [41] and its theoretical molecular weight can be found in the venom proteome, at RT 13.88.

Here we compared the sequences of AMPs found in *U*. *yaschenkoi* with those reported (19 sequences) from the transcriptome of *Urodacus manicatus* [49]. Data analysis shows that the subfamily of peptides having the most shared similarities between these two species is the short antimicrobial peptides, NDBP-5 (Fig 4). Sequences CYLIP-Uro-1, CYLIP-Uro-2, CYLIP-Uro-3, CYLIP-Uro-4 and CYLIP-Uro-5 from *U*. *manicatus* have structural similarities with those coding for UyCT1 and UyCT3 antimicrobial peptides previously reported from *U*. *yaschenkoi* [41]. In this work, several new UyCT1-like and UyCT3-like peptides were identified within the *U*. *yaschenkoi* transcritpome (Fig 4). Interestingly, there is a mature peptide sequence having 100% sequence identity within both species of scorpions. The mature peptide sequence is ILSAIWSFIKSLF and can be found in comp17_c0_seq1-4 and comp18_c0_seq1-2 from *U*. *yaschenkoi* and in CYLIP-Uro-2 from *U*. *manicatus*. However, the precursors in those sequences are different (Fig 4). The percentage of identity of the mature antimicrobial peptides (NDBP-5) relative to the ILSAIWSFIKSLF sequence ranged from 23 to 100% identity showing the diversity of gene sequences.

Results of the comparative analysis also showed that the opistoporine-like peptides of both species of scorpions are similar. The sequence comp42_c0_seq1 (Table 2) of *U*. *yaschenkoi* compares well with Csab-Uro4 from *U*. *manicatus* and the sequence of comp336_c0_seq1 (Table 2) from *U*. *yaschenkoi* is equivalent to Csab-Uro3 from *U*. *manicatus*. These sequences show similarities with opiscorpine-3 from *Opistophthalmus carinatus* [72] and peptide SC11 previously reported for *U*. *yaschenkoi* [41].

The fact that all the antimicrobial peptides reported for *U*. *manicatus* had a match with the *U*. *yaschenkoi* transcriptome was expected because these two scorpions belong to the same genus and family of scorpion, both found in Australia.

As previously mentioned, the abundant presence of AMPs peptides in the venoms of non-Buthidae scorpions, is a particular characteristic of these species. In addition, these AMPs are example of leading components with potential application as antibiotics due to their demonstrated antimicrobial activity.

#### Scorpine-like peptides

Scorpine, the first kind of this peptide described in the literature, is structurally a hybrid protein containing amino acid sequence similar to AMPs and K^+^-channel blocking peptides. It was purified from the venom of the scorpion *Pandinus imperator* and shown to be a potent anti-malarial agent against *Plasmodium bergei* [51].

The N-terminal domain of Scorpine is a linear segment capable of forming α-helix with cytolitic and antimicrobial activities, whereas the C-terminal domain is tightly linked by three disulfide bridges and was shown to have a β-KTx activity against potassium channels [73]. The presence of two distinct domains in this type of scorpine-like peptides [74] makes difficult to classify them as either AMPs or K^+^-channel specific peptides. For this reason they were initially called orphan peptides [73].

**Fig 5:**
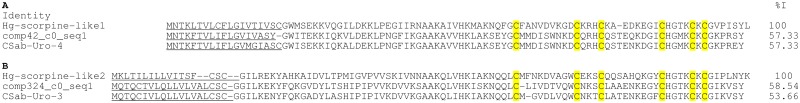
Scorpine-like peptides found in the *Urodacus yaschenkoi* transcriptome. A) The sequence obtained from the *U*. *yaschenkoi* transcriptome that codifies for a scorpine type 1 is shown and it is aligned with the reference scorpine Hg-scorpine-like1 from *Hadrurus gertschi*. Sequence CSab-Uro-4 from *Urodacus manicatus* [49] codifies as well for a scorpine type 1 and is included in the alignment. B) The sequence comp324_c0_seq1 found in the *U*. *yaschenkoi* transcriptome that codes for a scorpine type 2 is shown and aligned with Hg-scorpine-like2 from *H*. *gertschi*. Also, sequence CSab-Uro-3 from *U*. *manicatus* [49] is included. Both alignments show the percentage of identity of each sequence with respect to the reference sequence. The cysteine pattern (6 Cys) is highlighted in yellow. Note: comp42_c0_seq1 and comp47_c0_seq1 (not shown) code for the same precursor, comp324_c0_seq1 and comp336_c0_seq1 (not shown), code for the same precursor.

In this transcriptomic analysis four sequences similar to the precursors of scorpine-like peptides were found (Fig 5). Two different sequences: comp42_c0_seq1 and comp47_c0_seq1 from *U*. *yaschenkoi* encode the same scorpine-like, called type 1, because it shows 57.33% identity with Hg-scorpine-like-1 from *H*. *gertschi*, and also has 94.74% identity with CSab-Uro-4 from *U*. *manicatus* (Fig 5A). The other two sequences from *U*. *yaschenkoi*: comp324_c0_seq1 and comp336_c0_seq1 code for the same scorpine-like type 2; which shows a longer stretch of amino acids. This sequence has 58.54% identity with Hg-scorpine-like2 of *H*. *gertschi* and 84.34% identity with CSab-Uro-3 from *U*. *manicatus*, both defined as scorpine-like toxins (Fig 5B).

We had foreseen the existence of these scorpine-like sequences in the transcriptome of *U*. *yaschenkoi*. In fact, only the non-Buthidae scorpions have been reported, thus far, to contain scorpine-like peptides. However, due to the dual structural characteristics of these peptides, some erroneous classification of peptides from Buthidae families of scorpions were said to be scorpine-like components, when in reality they should have been classified simply as putative K^+^-channel toxins, because they show sequence similarities only with the C-terminal domain of scorpine.

Finally, the bi-functionality of the scorpine-like peptides found in venoms of scorpions is very interesting and promising for the possible development of drugs with antimicrobial and/or anti-malarial activity.

#### Toxin-like components

Another well represented group of sequences corresponds to toxin-like ones. They comprehend 15% of the analyzed transcripts (Table 2). This class of putative toxins has several cysteines that may form disulfide bridges and have been found in several scorpion transcriptomes. For many of them, their function has not been directly evaluated. The majority of toxin-like components were identified in transcriptomes of Buthidae scorpions (12–37) and to a lesser extend those from non-Buthidae scorpions (19, 21, 38–49).

#### Sequences similar to potassium channel specific toxins

Potassium channel toxins (KTxs) are well known scorpion venom components. They are found in Buthidae and non-Buthidae species. A common feature of these peptides is the presence of one segment of α-helix and three β-sheet structures cross-linked and stabilized by disulfide bridges (Cs α/β structure), which forms 3 and/or 4 disulfide bridges, and were classified as α-, β-, γ- and κ-KTxs [75, 76]. The most abundant and best studied are the peptides of the family α-KTx, from which more than 140 different peptides are known and were sub-divided into 30 subfamilies (http://www.uniprot.org/docs/scorpktx and [76]. Usually they are short peptides containing 23 to 42 amino acids, whereas the β-KTxs are longer, with more than 50 amino acid residues in their primary structures. Examples of β-KTxs are peptide TsTx-Kβ of the scorpion *Tityus serrulatus* and BmTXKβ of *Buthus marthensii* Karsch. The first is a blocker of Kv1.1 potassium channels, which shows an IC_50_ of 96 nM [77]. A recombinant format of peptide BmTXKβ [78] was expressed heterologously and shown to be a *bona fide* blocker of potassium channel.

**Fig 6:**
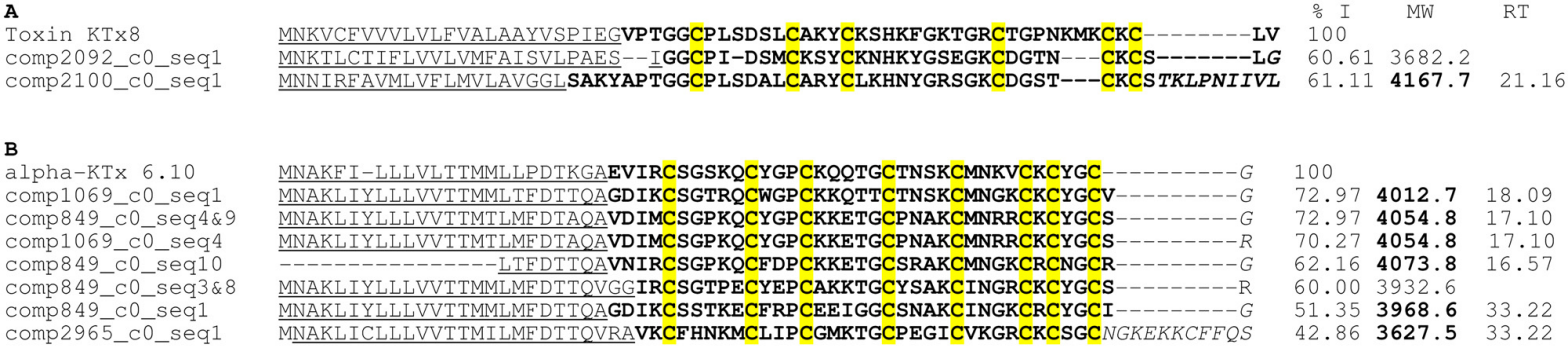
Putative alpha-KTx toxins from *U*. *yaschenkoi*. (A) Alignment of sequences found in the transcriptome of *U*. *yaschenkoi* that code for putative α-KTXs with six cysteines. These sequences are compared against the alpha-toxin KTx8 (UniProtKB/Swiss-Prot: A9QLM3.1) from *Lychas mucronatus*. Note: The precursor encoded by comp2100_c0_seq1 is also encoded by sequences comp1991_c0_seq1 to seq5 (not shown). (B) Alignment of sequences from *U*. *yaschenkoi* that code for alpha-potassium toxins with eight cysteines compared against the α-KTx 6.10 toxin (UniProtKB/Swiss-Prot: Q6XLL5.1) from *Opistophthalamus carinatus*. For all sequences, the percentage of identity of *U*. *yaschenkoi* mature peptides (%I) is shown in relation to the toxin of reference. The theoretical molecular weight (MW) of each *U*. *yaschenkoi* peptide is also shown. The signal peptide is underlined; the mature peptide is in bold; the residues probably involved in amidation and the propeptide are indicated in italics. The conserved cysteines are highlighted in yellow. The symbol “&” indicates that the sequence was encoded by two different nucleotide sequences (transcripts). MW in bold indicates that this molecular weight was found in the venom mass fingerprint previously reported [41] and is indicated the retention time (RT) in which it was found. Note: comp849_c0_seq6 (not shown) codes for the same precursor as sequence comp1069_c0_seq1; comp1069_c0_seq5 (no t shown) codes for the same precursor as sequence comp849_c0_seq3&8; comp1069_c0_seq3 (not shown) and comp849_c0_seq5 (not shown) code for the same precursor as comp849_c0_seq1; comp2981_c0_seq1 (not shown) codes for the same precursor as comp2965_c0_seq1.

The transcriptomic analysis of *U*. *yaschenkoi* allowed the identification of 17 different sequences with structural similarities with other known blockers of voltage-gated potassium channels that belong to the short potassium channel blocker scorpion toxin family. Within these sequences, 13 are similar to α-KTx-6 subfamily that is characterized by having 4 disulfide bridges. In fact, a complete precursor of urotoxin, which was previously reported by our group [79], was among these 13 sequences described here. Additionally, four sequences similar to toxins of the α-KTx family containing 3 disulfide bridges were also found (Fig 6). These sequences showed 60% identity with the α-KTx8 toxin of the scorpion *Lychas mucronatus* [80].

**Fig 7:**
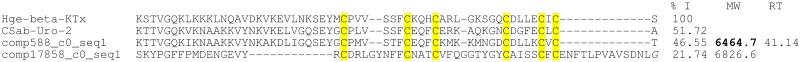
Putative β-KTxs found in *U*. *yaschenkoi* transcriptome. Multiple alignments of *U*. *yaschenkoi* sequences that code for putative β-KTx. These sequences belong to the long chain scorpion toxin family, Class 2 subfamily. *U*. *yaschenkoi* β-KTx sequences are compared against CSab-Uro-2 from *U*. *manicatus* and with the Hge-β-KTx from *Hadrurus gertschi*. All these sequences have 6 cysteines (highlighted in yellow). The percentage of identity (%I) is shown in relation to Hge-β-KTx. The theoretical molecular weight (MW) of *U*. *yaschenkoi* toxins is shown. In bold, the MW found in the mass fingerprint previously reported [41]. The retention time (RT) of this component is also indicated. Note: Sequence comp596_c0_seq1 (not shown) codes for the same mature peptide as the sequence comp588_c0_seq1 from this alignment.

Concerning the identification of β-KTxs Class 2 subfamily of potassium channel blocker toxins, 3 sequences were found that code for two putatives β-toxins, showing similarities with CSab-Uro-2 from the scorpion *U*. *manicatus* and Hge-beta-KTx of *Hadrurus gertschi* (Fig 7). All these sequences show to contain 6 cysteines sharing 21% to 51% identity with that of Hge-beta-KTx.

The number of sequences that are assumed to code for putative KTxs toxins found in this work is greater than those reported for other non-Buthidae scorpions. Only one α-KTx transcript was identified in *O*. *cayaporum* [46], and eight in the scorpion *S*. *jendeki* [45]. Concerning the putatives β-KTx only one was identified in the transcriptomic analysis of *H*. *gertschi* [48] and one in *P*. *cavimanus* [40]. However, this was expected due to the methodology used in the present work compared with the techniques used in the other mentioned species (cDNA library and Sanger sequencing).

#### Venom Proteins

Additional sequences representing 9% of the transcripts of this work correspond to proteins containing more than 70 amino acid residues and show similarities annotated as “venom proteins” of other transcriptomic analysis, such as venom protein-5 and venom protein-2 of the scorpion *Mesobuthus eupeus* (GenBank: ABR21071.1 and ABR21036.1, respectively) and SCO-spondin-like from *Bombyx mori* (NCBI Reference Sequence: XP_004924398.1), as shown in Table 2. This type of sequences has been found both in Buthidae and non-Buthidae scorpions. They might be present in any scorpion family, contrary to what was described previously concerning AMPs or α- and β-KTxs.

#### Calcins

**Fig 8:**
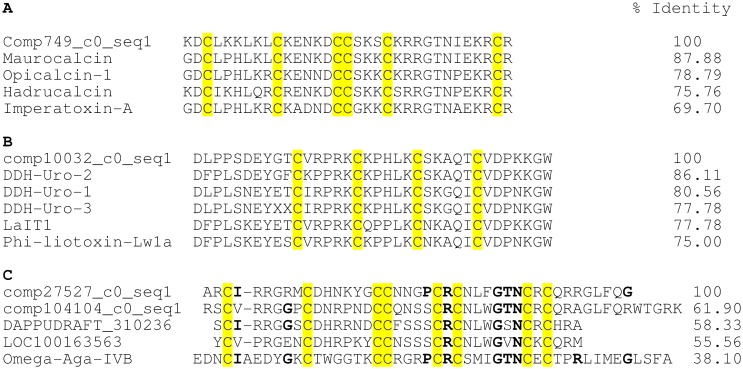
Putative calcium channel specific toxins found in *U*. *yaschenkoi* transcriptome. Three different multiple alignments of sequences are shown that code for: (A) calcins. Comp749_c0_seq1 codes for a calcin of 33 amino acid similar to other scorpion calcins as Hadrucalcin (UniProtKB/Swiss-Prot: B8QG00.1) from *Hadrurus gertschi*, Imperatoxin-A (UniProtKB/Swiss-Prot: P59868.1) from *Pandinus imperator*, Maurocalcin (UniProtKB/Swiss-Prot: P60254.1) from *Scorpio maurus palmatus* and Opicalcin-1 (UniProtKB/Swiss-Prot: P60252.1) from *Opistophthalmus carinatus*. All of them have 6 conserved cysteine; (B) LaIT1-like calcins. Sequence comp10032_c0_seq1 of *U*. *yaschenkoi* is compared with DDH-Uro-1 (GenBank: GALI01000015.1), DDH-Uro-2 (GenBank: GALI01000016.1) and DDH-Uro-3 (GenBank: GALI01000017.1) from *U*. *manicatus*, Insecticidal toxin LaIT1 from *Liocheles australasiae* (UniProtKB/Swiss-Prot: P0C5F2.1) and Phi-liotoxin-Lw1a (UniProtKB/Swiss-Prot: P0DJ08.1) from *Liocheles waigiensis*. This class of calcins has four cysteines. The sequence reported for DDH-Uro-3 contains undefined nucleotides and therefore the XX undefined amino acids. Finally, (C) Omega Agatoxin-like calcins. Putative calcium channel specific toxins encoded by sequences Comp27527_c0_seq1 and comp104104_c0_seq1 from *U*. *yaschenkoi* are shown and compared with DAPPUDRAFT_310236 of *Daphnia pulex*, LOC100163563 of insect *Acyrthosiphon pisum* and Omega Agatoxin IVB (Omega-Aga-IVB; GenBank: P37045) of spider *Agelenopsis aperta*. This class of calcin has eight cysteines. The percentage of identity (% Identity) is shown for all alignments with respect to the first sequence of each alignment. Conserved cysteines and amino acids are highlighted in yellow and bold, respectively.

Different types of proteins that control calcium ion permeability across biological membranes are known, and are defined as calcium channels. Among these are the voltage-gated, voltage-independent and ligand-activated channels. The last ones include the ryanodine sensitive calcium channels (RyRs) of the endoplasmic reticulum of heart and skeletal muscle, which are recognized by some peptides found in the venom of scorpions and are generically named calcins [5]. The first calcins characterized were the imperatoxins IpTxi and IpTxa, isolated from the venom of the African scorpion *Pandinus imperator* [5]. Imperatoxin A (IpTxa) is a 33 amino acid long peptide stabilized by three disulfide bridges, structurally organized in a special folding arrangement known as the “inhibitor cysteine knot”. This three-dimensional folding is commonly found in toxins from spiders and snails that affect voltage-dependent calcium channels [81] [82]. IpTxa affects the RyRs receptors modifying the channel activity [5]. Other calcins with similar structure and function were also isolated and characterized, such as: hemicalcin, opicalcin-1, opicalcin-2, hadrurin and maurocalcin (revised in [83]). The sequence Comp749_c0_seq1 identified in the transcriptome of *U*. *yaschenkoi* (Table 2) codifies for a putative imperatoxin-A-like calcin. It has 33 amino acids, six cysteines and shares 69% of identity with imperatoxin-A and 87% with maurocalcin (Fig 8A). Furthermore, the scorpion *Liocheles australasiae* has a peptide called toxin LaIT1, described to affect the function of the RyRs channels. This peptide has 36 amino acid residues, similar to the known calcins, but is rather toxic to insects than to mammalians [84]. The peptide Phi-liotoxin-Lw1a, isolated from the Australian scorpion *Liocheles waigiensis* [85], also affect the activity of both ryanodine-sensitive calcium channels RyR1 and RyR2 with high potency and has sequence similarities with LaIT1. Its structure shows two-stranded beta-sheets or DDH for disulfide-directed beta-hairpin, stabilized by 2 disulfide bridges [86]. In the present work with *U*. *yaschenkoi*, the sequence comp10032_c0_seq1 is a LaIT1-like peptide. It has 36 amino acid residues with four cysteines and shows sequence identity over 75% with the other LaIT1-like peptide (Fig 8B). The sequence comp10032_c0_seq 1 of *U*. *yaschenkoi* resembles the three DDH-like peptides reported from *U*. *manicatus*: DDH-Uro1, DDH-Uro2 and DDH-Uro3 (Fig 8B). The high degree of similarity of these peptides is certainly due to the fact that they belong to related Australian scorpions.

Finally, four sequences were found in the transcriptome of *U*. *yaschenkoi* code for peptides similar to spider toxins such as Omega-agatoxin-IVB from *Agelenopsis aperta*. This peptide blocks P-type calcium channels of cerebellar Purkinje neurons [87]. The same peptide paralyzes insects by blocking the neuromuscular transmission. The peptides of the transcripts found in the transcriptome of *U*. *yaschenkoi* here described, are thought to share structural similarities to the cystine knots of spider toxins, which are formed by a triple-stranded antiparallel beta-sheet, stabilized by 4 disulfide bridges. The sequences comp27527_c0_seq1 and comp10414_c0_seq1 code for a 42 and 47 amino acid agatoxin-like calcins, respectively (Fig 8C). In these sequences the 8 cysteines are conserved, including the double CC sequence and the triple amino acid GTN of these calcins (Fig 8C).

In conclusion, the data reported here indicate that *U*. *yaschenkoi* is the first scorpion whose transcriptomic analysis of the venom gland shows sequences that code for three distinct types of putative calcins. In addition, it also suggests that non-Buthidae species of scorpion seems to contain more calcin-like peptides than the Buthidae species.

#### La1-like peptides

**Fig 9:**
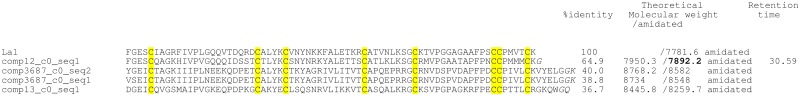
La-1-like peptides found in the *U*. *yaschenkoi* transcriptome. The alignment compares the La-1-like peptides encoded by *U*. *yaschenkoi* transcripts with the model La1 peptide from *Liocheles australasiae*. La1 is amidated with a molecular weight of 7781.6 Da. Only the theoretical molecular weight amidated of comp12_c0_seq1 (in bold) was detected in the proteome of *U*. *yaschenkoi* [41], in retention time 30.59. La1 peptide has eight cysteines that are conserved in all the putative La-1-like peptides found herein (highlighted in yellow). The percentage of identity with respect to La1 is shown. Note: comp12_c0_seq1 and comp15_c0_seq1 (not shown) encode the same peptide; comp3687_c0_seq2 and comp4167_c0_seq2 (not shown) encode the same peptide; comp3687_c0_seq1 and comp4167_c0_seq1 (not shown) code for the same peptide and comp13_c0_seq1 and comp16_c0_seq1 (not shown) code for the same peptide.

The venom of the scorpion *Liocheles australasiae* apart from the insect toxin peptide described in the precedent section also contains a very abundant peptide, simply called La1. It is the most abundant component of the venom. It is composed by 73 amino acid residues with 8 cysteines forming 4 disulfide bridges. This peptide was assayed for possible insecticide activity on crickets and mammalian specific toxicity in mice, without any effect (74). Similar peptides were reported to exist in transcriptomic analysis of the scorpions of the species *O*. *cayaporum*, *P*. *cavimanus*, *S*. *margarisonae*, *H*. *petersii* and *Scorpio maurus palmatus* [19, 39, 40, 43, 46]. In the transcriptomic analysis of *U*. *yaschenkoi*, we have identified 8 sequences that code for precursors similar to La1. They all show to contain 8 cysteines and sequences identities varying from 36 to 64% of that of La1 of *L*. *australasiae* (Fig 9). Unfortunately their abundant presence and possible function still remains unknown.

#### Ascaris-Type Protease inhibitors

Proteins with proteolytic activity are found in all living organisms, from bacteria to arthropods, plants and vertebrate animals, with a large number of activities and specificities, which are exquisitely controlled by inhibitors, in order to avoid unspecific proteolytic digestion of other existing proteins in their living cells. They are found in the body of organisms, but also in their secretions (revised in [88]). Peptides with protease inhibitor activity were described to be present in scorpion venoms, specially the Kunitz-type peptides. Some of which were reported as ion channel blockers, also known as Kunitz-type toxins. The first described is the peptide SdPI of the scorpion *Lychas mucronatus* [89]. Other Kunitz-like peptides were described in transcriptomic analysis of scorpions, such as: LmKTT-1.a, LmKTT-1.b, and LmKTT-1.c from *Lychas mucronatus* [90]; BmKTT-2, BmKTT-3, BmKTT-1 from *Mesobuthus martensii* [37, 90] and Hg1 from *Hadrurus gertschi* [48, 91].

**Fig 10:**
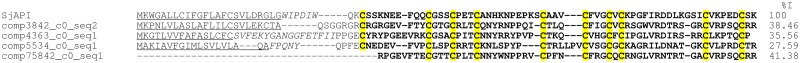
Comparison of Ascaris-type protease inhibitor peptides found in *U*. *yaschenkoi* transcriptome. Multiple alignment of *U*. *yaschenkoi* sequences that code for ascaris-type protease inhibitor compared against the ascaris-type protease inhibitor precursor from *Scorpiops jendeki* scorpion (SjAPI; GenBank: P0DM55). The sequence of comp75842_c0_seq1 is partial. The predicted signal peptide is underlined; putative propeptides is in italics and the common trypsin inhibitor like cysteine rich domain in bold with its ten cysteines highlighted in yellow. The percentage of identity (%I) with respect to SjAPI is shown. Note. Sequence comp4363_c0_seq1 encodes the same precursor as sequence comp4053_c0_seq1 (not shown); comp5534_c0_seq1 encodes the same precursor as comp4356_c0_seq1 (not shown) and comp135491_c0_seq1 (not shown) codes for the same partial sequence as comp75842_c0_seq1.

However, the Kunitz-type protease inhibitor peptides are not the only ones present in the venom gland of scorpions. The peptide SjAPI identified in *Scorpiops jendeki* is capable of inhibiting serine-proteases. It shows a structural folding similar to the “Ascaris-type inhibitor” [88]. In *U*. *yaschenkoi* transcriptome we have found 7 distinct sequences that are assumed to code for putative Ascaris-type protease inhibitors (Fig 10). The seven sequences reported here show similarities to peptide SjAPI with identities from 27 to 41%; all of them having 10 cysteine residues like the Ascaris-type inhibitors (Fig 10).

The venom gland of both Buthidae and non-Buthidae scorpions contain protease inhibitors. It is assumed that their principal function is to protect the other venom components from being degraded and play an important role for survival of the venomous animals [92–94].

#### Allergens

Sequences that are assumed to code for venom allergens were found at the level of 2% of the transcripts. Allergens are known to occur in the venom of: bee, wasp, ant, spider and centipede [95–98]. The first described was the bee venom allergen-5, which was reported to cause allergy in humans [99]. In the transcriptome described here we encountered 4 nucleotide sequences thought to code for different allergens (Table 2). The sequences found in *U*. *yaschenkoi* have close to 30% identity with allergen-5 of *Tityus serrulatus*, which is composed of 212 amino acid residues and contains disulfide bridges (UniProtKB/Swiss-Prot: P85840.1). For two sequences, comp4029_c0_seq1 and comp4170_c0_seq1 (see Table 2); it was possible to identify the signal peptide and the mature peptide of these allergen-like sequences, which have 237 and 236 amino acid residues, respectively. These sequences have similarities with CAP-Uro-1 y CAP-Uro-2 from the scorpion *U*. *manicatus* [49]. From this analysis it is clear that allergens are present in Buthidae and non-Buthidae scorpion venoms.

#### Sodium-channel specific toxins

Scorpion toxins specific for Na^+^-channels (NaTxs) recognize and modulate the function of sodium channels of excitable and non excitable cells and are the most important venom components medically speaking, because are the ones responsible for the intoxication symptoms of humans (7). They contain usually 58–76 amino acid residues tightly cross-linked by 4 disulfide bridges. Two main physiological functions are described for these toxic peptides: alpha-toxins (α-NaScTxs) and beta-toxins (β-NaScTxs). They both are modulators of the gating mechanism of Na^+^-channels. The first one prolong the action potential making the closing mechanism of the channel longer in time; the β-NaScTxs modify the open mechanism by producing an activation of the channel at less negative potentials (reviewed in [73]). At this moment, there is more than 300 known NaTxs, either directly isolated from scorpion venoms or identified based on gene cloning (see UniProt in www.uniprot.org). It is well known that these peptides occurs mainly on Buthidae species and are poorly represented in venoms from non-Buthidae species of scorpions (reviewed in [73]). Only three sequences assumed to code for putative NaTxs were found in *U*. *yaschenkoi* transcriptome (Table 2). This finding is in agreement with proteomic and transcriptomic studies conducted comparatively between Buthidae and non-Buthidae scorpions (revised in [83]).

### Comparison of transcriptome and proteome components found in *U*. *yaschenkoi*

In our previous proteomic work [41], the molecular masses of several components of *U*. *yaschenkoi* venom were obtained. Table 3 compares the values of 16 theoretical molecular weight expected of putative toxins and antimicrobial peptides deducted from the sequences obtained by the high-throughput transcriptome with 16 experimentally (LC-MS/MS) determined molecular weights of components identified by proteome analysis. From these correlations, eight putative potassium channel specific toxins from the subfamily α-KTx-3 were found and matched. We also found that the antimicrobial peptide UyCT3 and the putative antimicrobial peptide encoded by sequence comp1267_c0_seq1 are the same. A putative β-KTx toxin encoded by sequences comp588_c0_seq1 and that of comp596_c0_seq1 were coincident with the proteome found components. Similarly, the putative calcin encoded by sequences comp10032_c0_seq1 and comp11072_c0_seq1 are the same. Finally, peptide La1-like coded by sequence comp12_c0_seq1 matches with a peptide found in the proteome analysis (see Table 3).

**Table 3:** Correlation between theoretical molecular weight of transcriptome sequences and experimental molecular weight obtained from the proteome venom of *Urodacus yaschenkoi*.

RT (min)	Exp. MW (Da)	Theor.MW (Da)	Sequence Name of *U*. *yaschenkoi* transcriptome	Seq. description (BLAST)	Aminoacid sequence (mature peptide in bold)
13.88	1433.54	1433.7	comp17_c0_seq1-4	antimicrobial peptide UyCT3 ndpb precursor	MKNQFVLLLLAIVFLQLISQSDA **ILSAIWSGIKSLF** *GKRGLKNMDKFDELFDGDFSQADLDFLRELTR*
16.57	4073.81	4073.8	comp849_c0_seq10	alpha-KTx potassium channel blocker precursor	LTFDTTQA **VNIRCSGPKQCFDPCKKETGCSRAKCMNGKCRCNGCR*G***
17.10	4054.26	4054.8	comp849_c0_seq4&9; comp1069_c0_seq4	alpha-KTx potassium channel blocker precursor	MNAKLIYLLLVVTTMTLMFDTAQA **VDIMCSGPKQCYGPCKKETGCPNAKCMNRRCKCYGCS*G***
18.09	4012.20	4012.7	comp1069_c0_seq1; comp849_c0_seq6	alpha-KTx potassium channel blocker precursor	MNAKLIYLLLVVTTMMLTFDTTQA **GDIKCSGTRQCWGPCKKQTTCTNSKCMNGKCKCYGCV*G***
21.16	4167.08	4167.7	comp1991_c0_seq1-5; comp2100_c0_seq1	alpha-KTx precursor	MNNIRFAVMLVFLMVLAVGGLSA **KYAPTGGCPLSDALCARYCLKHNYGRSGKCDGSTCKCS** *TKLPNIIVL*
22.37	3968.20	3968.6	comp849_c0_seq1; comp849_c0_seq5	alpha-KTx potassium-channel inhibitor kcug2 precursor	MNAKLIYLLLVVTTMMLMFDTTQA **GDIKCSSTKECFRPCEEIGGCSNAKCINGKCRCYGCI*G***
23.64	4068.00	4068.7	comp10032_c0_seq1; comp11072_c0_seq1	calcium-channel txs2b_liowa	MNFSSKISFLLLVTAVVFA *VTGGEVDRLFEQYKESDIER* **DLPPSDEYGTCVRPRKCKPHLKCSKAQTCVDPKKGW**
28.41	6576.90	6573.4	comp395_c0_seq1	toxin-like toxin tx707	MLKTVIFCIAVLASVCTG **EENSEEGRTFPLLFSADGRNSLGCWITYSFSYQPTADIDTKIAAQNTLCECMKKGLVPK**
30.59	7891.57	7892.2	comp12_c0_seq1	la1-like protein 13 precursor	MERILKPVFLAILIVLSFSSQCMG **FGESCQAGKHIVPVGQQQIDSSTCTLYKCSNYNRKYALETTSCATLKLKSGCRMVPGAATAPFPNCCPMMMCK*G***
31.46	8543.12	8543.9	comp348_c0_seq1; comp299_c0_seq1	toxin-like tx11_opicy	MKVACSLVLLVAFTCTVSA **RVVSKKTCKTHTGVILRHGEEWKDPNHCSIYRCTIYDGEAELDGLMCATYQVPRNCKFVRGGGKLYPSCCPTVVCK**
32.53	7458.64	7458.3	comp330_c0_seq1	alpha-KTx potassium channel blocker precursor	MNAKLICLLLVVTTMILMFDTTQVRA**VKCFHNKMCLIPCGMKTGCPEGICVKGRCKCSGC** *NGKEKKCFFQS*
33.22	3627.00	3627.5	comp2965_c0_seq1; comp2092_c0_seq1	alpha-KTx potassium channel blocker precursor	MNAKLICLLLVVTTMILMFDTTQVRA**VKCFHNKMCLIPCGMKTGCPEGICVKGRCKCSGC** *NGKEKKCFFQS*
33.22	3967.20	3968.6	comp849_c0_seq1; comp849_c0_seq5; comp1069_c0_seq3	alpha-KTx potassium-channel inhibitor kcug2 precursor	MNAKLIYLLLVVTTMMLMFDTTQA **GDIKCSSTKECFRPCEEIGGCSNAKCINGKCRCYGCI*G***
34.80	4967.22	4967.7	comp6164_c0_seq1; comp6514_c0_seq1	toxin-like toxin tx277	MQFKRLLVALTLICIVSC **EEKRDSSGRSCSVTGICMKSCARFLHQPANHKKCLPDGVCCTLIY**
41.14	6464.16	6464.7	comp588_c0_seq1; comp596_c0_seq1	beta-ktx-like peptide	MAKHLLAEFLVIMLISSLADG **KTTVGQKIKNAAKKVYNKAKDLIGQSEYGCPMVSTFCEQFCKMKKMNGDCDLLKCVCT**
45.56	6512.47	6511.5	comp1267_c0_seq1	antimicrobial peptide c22 precursor	MNAKVMLVCLLVTMLVMEPAEA **GIWSWIKKTAKKVWNSDVAKKLKGKALNAAKDFVAEKIGATPAEAGQIPFDEFMNVLYS**

RT means retention time; min means minutes; Exp. MW is the experimental molecular weight; Theor. MW is the theoretical molecular weight; Da means Daltons; Signal peptide is shown underlined, putative precursor is shown in italics; amino acid amidated is shown in bold and italics.

A comparison between results of the cDNA library shotgun approach [41] and the results found with the NGS RNA-seq transcriptome showed that both techniques are reliable for characterization of venom components found in scorpion venom glands. The same type of family components was identified by both methodologies. As it can be seen in Fig 3 of [41] and in Fig 3 of this communication, the subfamilies of toxins and peptides are almost the same and furthermore, they have the same proportions. Both studies reports antimicrobial peptides (UyCT3), calcin-like (Contig 20), scorpine-like peptides, La-1–like peptides, alpha and beta potassium channel specific toxins. The NGS approach reported here allowed the identification of a greater number of mass sequences than the shotgun methodology reported earlier by our group, when the results are compared with the mass values obtained in the proteome analysis.

One might argue that the correlation found is somehow limited. However there are several plausible reasons that explain these findings: a) the crude venom used for the experiments reported previously [41] and the venom glands used for RNAseq studies reported here were not the same, b) the extracted vemom, during handling and isolation procedures might suffer small modifications, which does not allow a perfect match of the molecular masses determined in proteomic analysis; c) an important number of sequences of putative peptides and proteins obtained by transcriptome analysis are not fully characterized, thus it is difficult to predict the exact molecular mass expected; d) a few sequences could be subjected to post-translational modifications, which will again make difficult to predict the exact molecular mass expected.

### Comparison of this transcriptome with that of *Centruroides noxius* scorpion

A comparison of the data between *C*. *noxius* [16] and *U*. *yaschenkoi* transcriptomes resulted in 273 similar sequences. The percent pairwise identity of most sequences is above 75%. Similar sequences are mainly represented by enzymes or components involved in biological processes. For example: kinases, enolases, helicases, phosphatases, actins, zing finger proteins and RNA related proteins. It supports the conclusion that both species of scorpions share the same machinery to produce the venom, although the venom components are certainly different.

Both scorpions, *C*. *noxius* and *U*. *yaschenkoi*, have in their venom hyaluronidase enzymes, venom allergens, venom insulin-like growth factors and of course, venom toxins but for *C*. *noxius* the most represented are the sodium channel toxins while for *U*. *yaschenkoi* few putatives sodium channel toxins were identified.

One of the most abundant components identified from *U*. *yaschenkoi* transcriptome were the antimicrobial peptides (21%) whereas for *C*. *noxius* transcriptome only one isogroup containing 6 reads was similar to porine, an antimicrobial peptide. Once again, this finding was expected because non-Buthidae scorpions are a well known source of antimicrobial peptides [100–103] while Buthidae scorpions lack these compounds.

Despite the fact that studies related to scorpion venom components have been steadily increasing over the past four decades, the first whole transcriptome [16] and the first genome [22] of singular species have only recently been obtained. Both studies were made with Buthidae scorpions (*Centruroides noxius* and *Mesobuthus martensii*) and the results gave a general view of the cellular and molecular processes in the assembly of the scorpion venom components. Additionally, metabolic pathways and the dynamics of expansion of scorpion gene families were elucidated. On the contrary, for scorpions of non-Buthidae species, there is lack of similar information, especially at the transcriptomic level. The results reported here should be considered as an extended analysis of the genes expressed in the venom gland of an Urodacidae scorpion, filling in the missing information. This communication reports the whole transcriptomic analysis, in which hundreds of venom components are fully characterized, contributing to the large-scale discovery of scorpion toxin sequences and should serve as a reference for comparative studies and subjects related to evolution of venoms and venomous animals.

## Conclusion

A total of 210 different nucleotidic sequences that code for 111 unique and specific toxins, peptides and proteins in the *Urodacus yaschenkoi* venom were identified; some of them were previously found in the *U*. *yaschenkoi* cDNA library shotgun approach reported by our group. The correlation between the proteome and this data set permitted the identification of 16 theoretical molecular weights deducted from the whole transcriptome. An extended cross-reference to other scorpion known venom components is included. This work analyzed in detail the whole array of transcripts expressed in the venom gland of a non-Buthidae scorpion of the family Urodacidae. It is expected that the identification of these new toxins and peptides will contribute to the production of new putative bioactive compounds or pharmacological tools. Meanwhile, this dataset will serve as a public information platform to accelerate studies in venomics research and will serve as a reference for non-Buthidae scorpions.

## Supporting Information

S1 file Distribution of contigs and sequences from *Urodacus yashenkoi* venom gland. Size distribution of the *Urodacus yashenkoi* venom gland contigs obtained from the *de novo* assembly of high-quality clean reads (Fig A). Most abundant Go-terms for the sub-dataset containing only toxins and venom related components (Fig B). Pie charts with the most abundant Go term per domain for the sub-dataset containing only toxins and venom related components. Fig C-A: cellular component, Fig C-B: biological process and Fig C-C: molecular function (Fig C). Enzyme distribution for the sub-dataset containing only toxins and venom related components: Oxireductases, transferases, hydrolases, lyases, isomerases and ligases (Fig D). Most abundant families of enzymes found in the whole transcriptome (Fig E).
(PDF)

S1 Table FPKM for venom related compounds and housekeeping genes found in the whole transcriptome of *Urodacus yaschenkoi*. (PDF)
