# Supplementary figures and images for: Whole Transcriptome of the Venom Gland from Urodacus yaschenkoi Scorpion
Source: PLoS One. 2015 May 28;10(5):e0127883. doi: 10.1371/journal.pone.0127883 (PMC4447460; doi:10.1371/journal.pone.0127883)

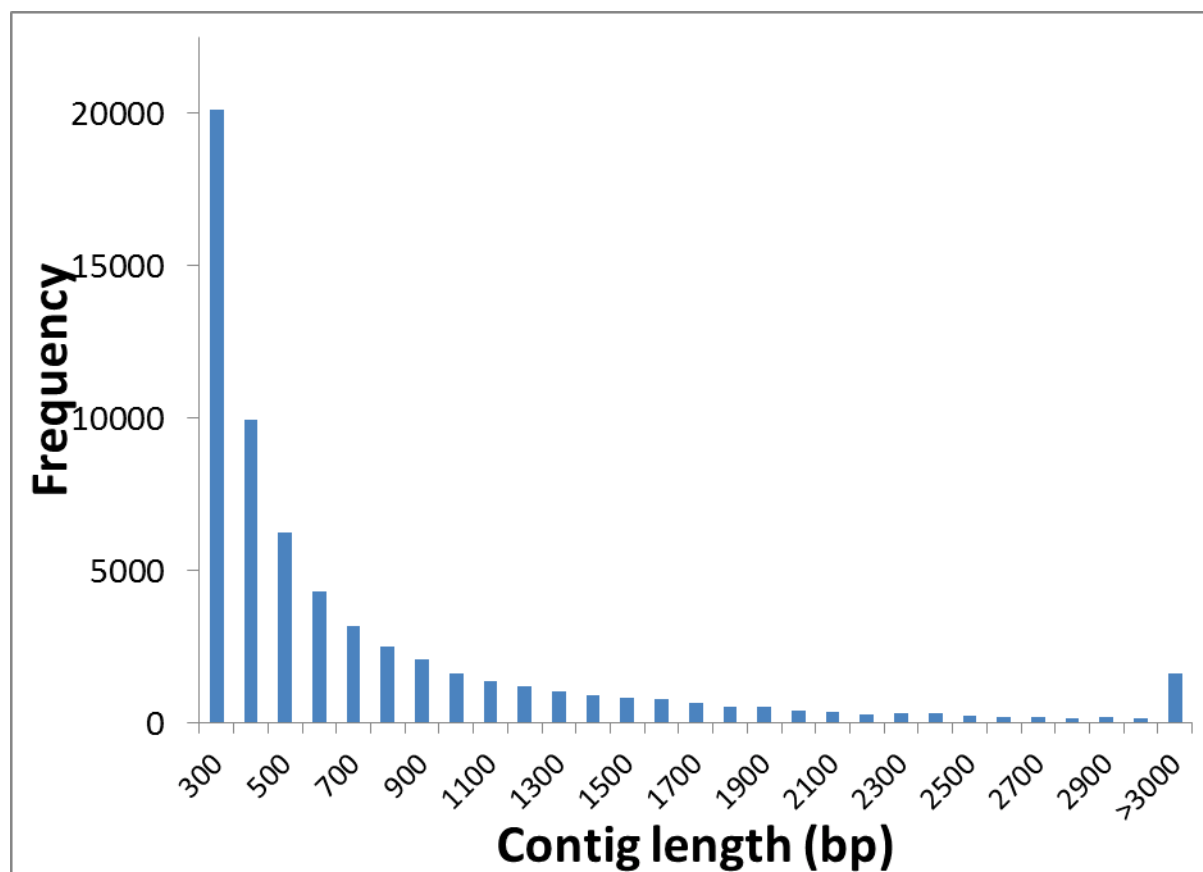

**Figure A**

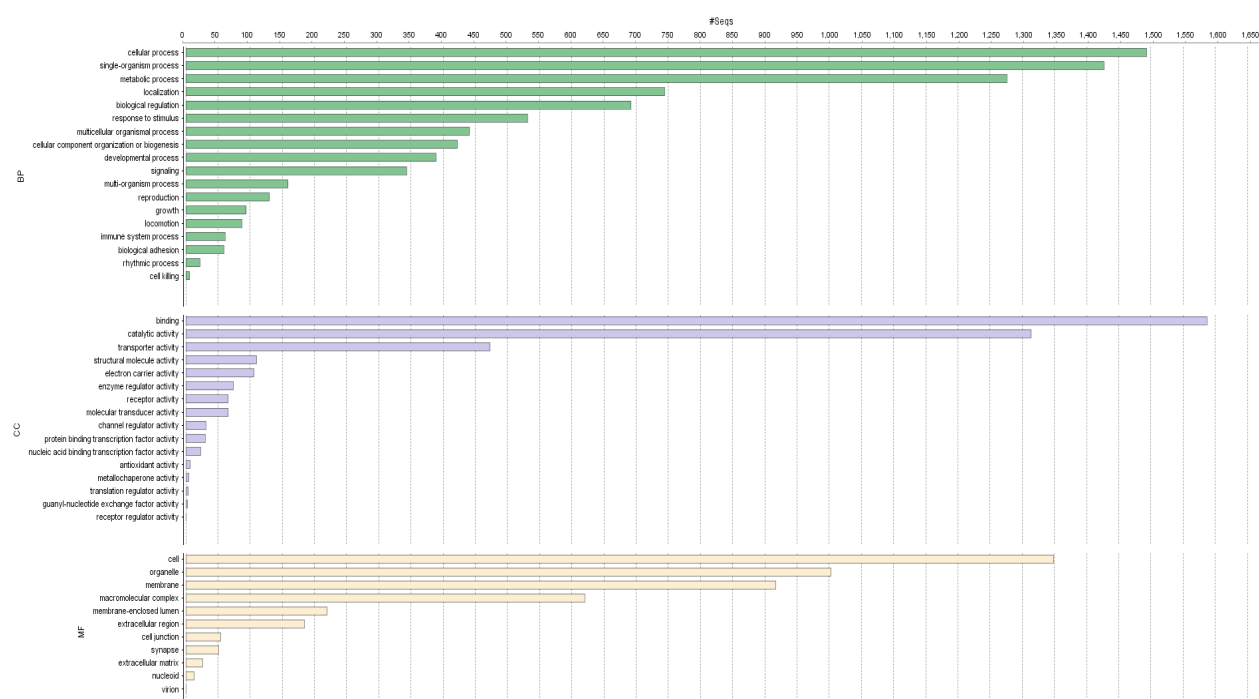

**Figure B**

A

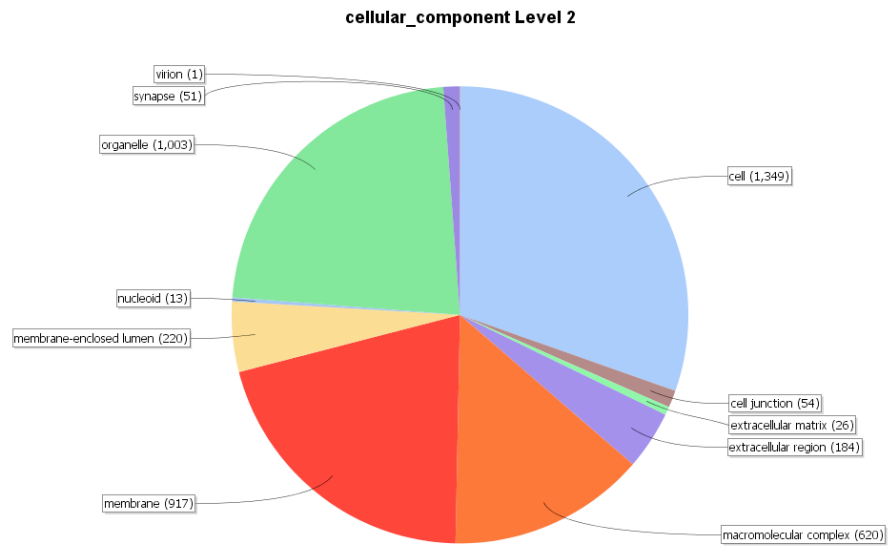

B

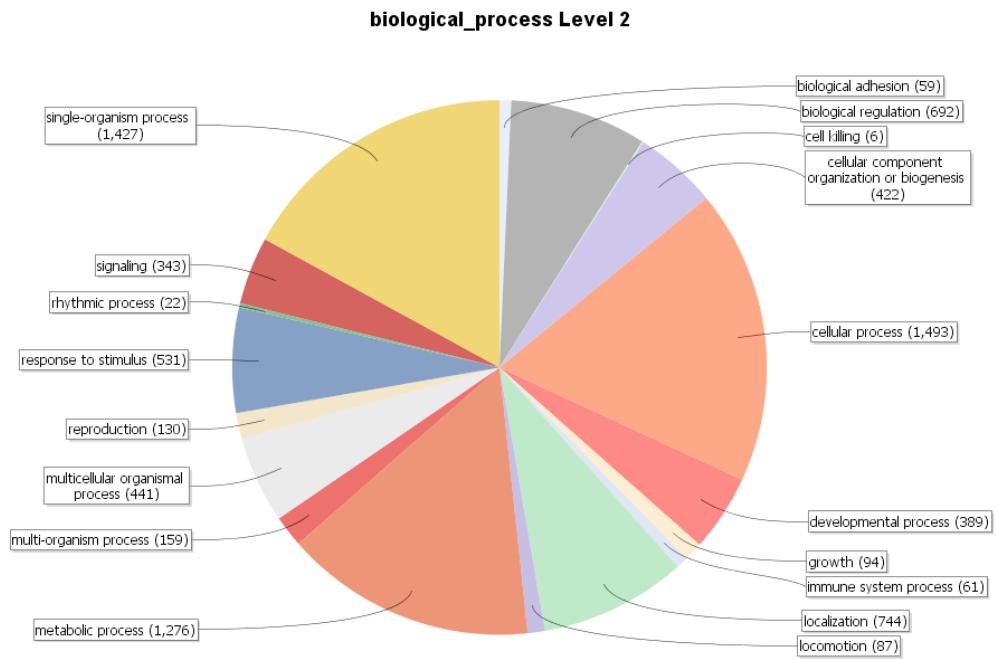

C

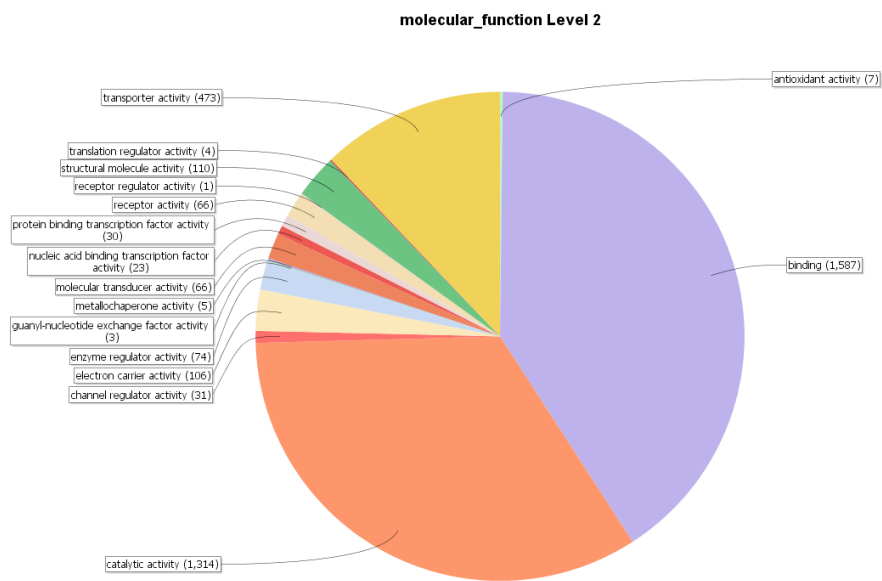

**Figure C**

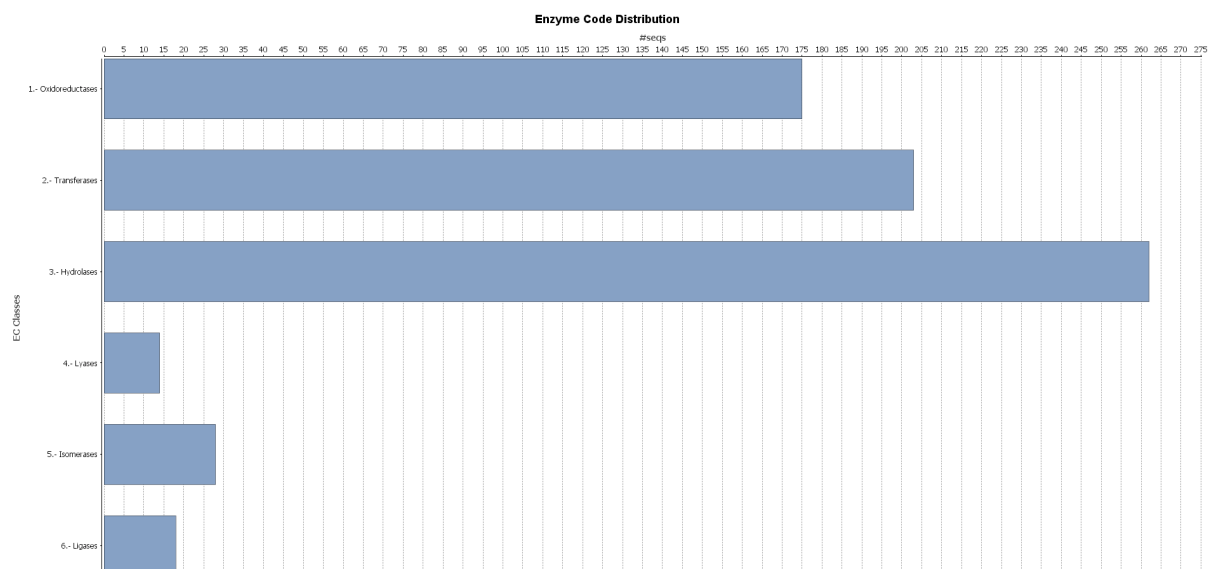

**Figure D**

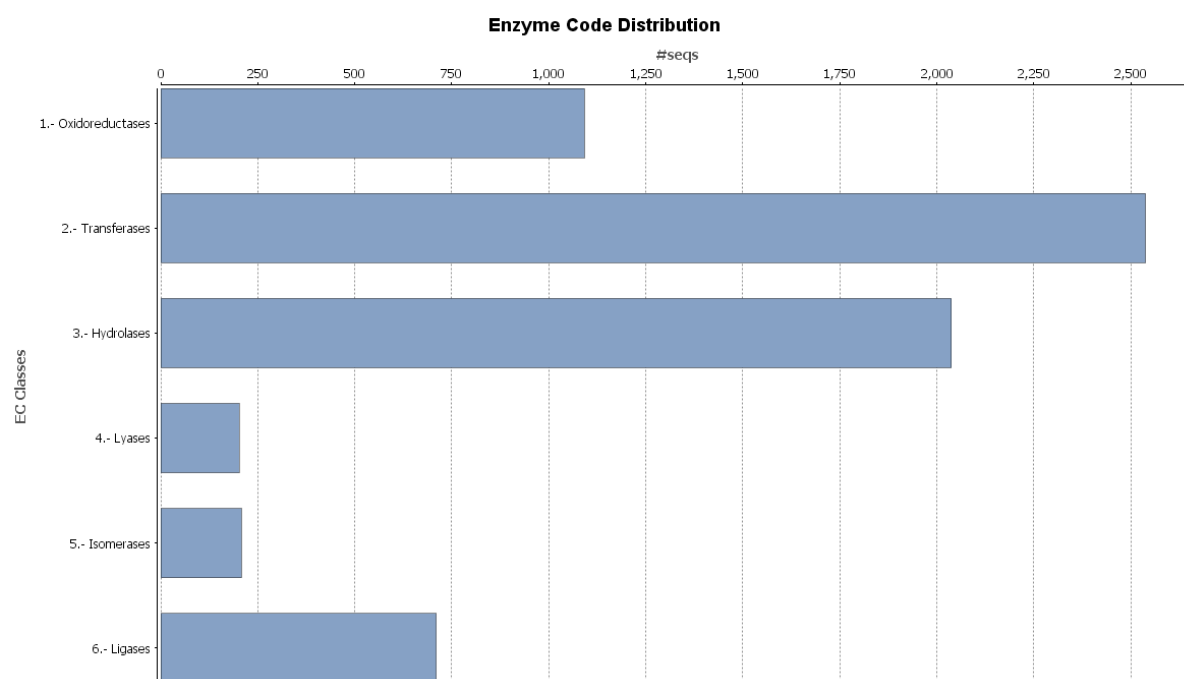

**Figure E**

Supplement: S1 file — Size distribution of the Urodacus yashenkoi venom gland contigs obtained from the de novo assembly of high-quality clean reads (Fig A). Most abundant Go-terms for the sub-dataset containing only toxins and venom related components (Fig B). Pie charts with the most abundant Go term per domain for the sub-dataset containing only toxins and venom related components. Fig C-A: cellular component, Fig C-B: biological process and Fig C-C: molecular function (Fig C). Enzyme distribution for the sub-dataset containing only toxins and venom related components: Oxireductases, transferases, hydrolases, lyases, isomerases and ligases (Fig D). Most abundant families of enzymes found in the whole transcriptome (Fig E). (PDF) [file pone.0127883.s001.pdf]
