# Supplementary material for: Whole Transcriptome of the Venom Gland from Urodacus yaschenkoi Scorpion
Source: PLoS One. 2015 May 28;10(5):e0127883. doi: 10.1371/journal.pone.0127883 (PMC4447460; doi:10.1371/journal.pone.0127883)
Supplement: S1 Table — (PDF) [file pone.0127883.s002.pdf]

Table S1. FPKM for venom related compounds and housekeeping genes found in the whole transcriptome of *Urodacus yaschenkoi*

Query id	Cufflinks id	FPKM	% identity	Subject annotation	E value
comp4_c0_seq1	CUFF.78703	2,743.21	54.55	hypothetical protein [Tityus discrepans]	9.00E-11
comp5_c0_seq1	CUFF.90526	2,806.60	54.55	hypothetical protein [Tityus discrepans]	9.00E-11
comp9_c0_seq1	CUFF.119409	2,470.06	94.09	actin [Opisthacanthus cayaporum]	5.00E-101
comp6_c0_seq1	CUFF.94394	88.98	74.43	cytochrome oxidase subunit I [Mesobuthus martensii]	2.00E-66
comp10_c0_seq1	CUFF.10033	1,148.58	94.09	actin [Opisthacanthus cayaporum]	9.00E-101
comp12_c0_seq1	CUFF.18582	2,685.49	27.96	venom toxin [Opisthacanthus cayaporum]	6.00E-09
comp13_c0_seq1	CUFF.23081	2,690.72	34.43	venom toxin [Opisthacanthus cayaporum]	2.00E-08
comp15_c0_seq1	CUFF.28735	2,892.89	27.96	venom toxin [Opisthacanthus cayaporum]	5.00E-09
comp16_c0_seq1	CUFF.31381	3,295.27	34.43	venom toxin [Opisthacanthus cayaporum]	2.00E-08
comp17_c0_seq1	CUFF.32825	133.13	78.00	antimicrobial peptide NDPB 5.7 precursor [Opisthacanthus cayaporum]	2.00E-18
comp17_c0_seq2	CUFF.32803	172.32	78.00	antimicrobial peptide NDPB 5.7 precursor [Opisthacanthus cayaporum]	2.00E-18
comp17_c0_seq3	CUFF.32716	713.74	78.00	antimicrobial peptide NDPB 5.7 precursor [Opisthacanthus cayaporum]	3.00E-19
comp17_c0_seq4	CUFF.32989	1,890.50	78.00	antimicrobial peptide NDPB 5.7 precursor [Opisthacanthus cayaporum]	3.00E-19
comp17_c0_seq5	CUFF.32724	2,039.06	76.00	antimicrobial peptide NDPB 5.7 precursor [Opisthacanthus cayaporum]	4.00E-13
comp18_c0_seq1	CUFF.35187	149.10	78.00	antimicrobial peptide NDPB 5.7 precursor [Opisthacanthus cayaporum]	2.00E-18
comp18_c0_seq2	CUFF.35076	1,159.45	78.00	antimicrobial peptide NDPB 5.7 precursor [Opisthacanthus cayaporum]	3.00E-19
comp18_c0_seq3	CUFF.34815	426.59	76.00	antimicrobial peptide NDPB 5.7 precursor [Opisthacanthus cayaporum]	3.00E-13
comp18_c0_seq4	CUFF.34813	575.78	75.00	antimicrobial peptide NDPB 5.7 precursor [Opisthacanthus cayaporum]	4.00E-12
comp19_c0_seq1	CUFF.36517	845.79	62.39	ATP synthase F0 subunit 6 [Mesobuthus martensii]	6.00E-57
comp20_c0_seq1	CUFF.38224	380.77	46.88	NADH dehydrogenase subunit 2 [Mesobuthus martensii]	8.00E-49
comp20_c0_seq2	CUFF.38133	283.68	46.88	NADH dehydrogenase subunit 2 [Mesobuthus martensii]	7.00E-49
comp20_c1_seq1	CUFF.39416	1,923.59	78.54	cytochrome oxidase subunit I [Mesobuthus martensii]	5.00E-76
comp21_c0_seq1	CUFF.41479	658.60	62.39	ATP synthase F0 subunit 6 [Mesobuthus martensii]	5.00E-57
comp31_c0_seq1	CUFF.52364	319.67	64.58	antimicrobial peptide NDPB 5.8 precursor [Opisthacanthus cayaporum]	2.00E-14
comp31_c0_seq2	CUFF.52276	229.91	64.58	antimicrobial peptide NDPB 5.8 precursor [Opisthacanthus cayaporum]	1.00E-14
comp31_c0_seq3	CUFF.52296	220.80	64.62	antimicrobial peptide NDPB 5.8 precursor [Opisthacanthus cayaporum]	2.00E-21
comp31_c0_seq4	CUFF.52376	376.89	68.33	antimicrobial peptide NDPB 5.8 precursor [Opisthacanthus cayaporum]	2.00E-20
comp31_c0_seq5	CUFF.52268	247.60	70.00	antimicrobial peptide NDPB 5.8 precursor [Opisthacanthus cayaporum]	2.00E-20

comp35_c0_seq1	CUFF.56995	794.20	66.67	antimicrobial peptide NDPB 5.8 precursor [Opisthacanthus cayaporum]	9.00E-20
comp35_c0_seq2	CUFF.57097	204.56	64.62	antimicrobial peptide NDPB 5.8 precursor [Opisthacanthus cayaporum]	2.00E-21
comp39_c0_seq1	CUFF.62094	1,312.76	64.35	hypothetical protein [Opisthacanthus cayaporum]	1.00E-31
comp42_c0_seq1	CUFF.65601	436.77	40.00	beta-KTx-like peptide [Tityus discrepans]	3.00E-17
comp43_c0_seq1	CUFF.66516	1,329.39	64.35	hypothetical protein [Opisthacanthus cayaporum]	1.00E-31
comp44_c0_seq1	CUFF.68622	1,144.10	69.13	elastase-like protein [Opisthacanthus cayaporum]	7.00E-93
comp45_c0_seq1	CUFF.69694	1,138.22	69.13	elastase-like protein [Opisthacanthus cayaporum]	8.00E-93
comp47_c0_seq1	CUFF.71507	436.82	40.00	beta-KTx-like peptide [Tityus discrepans]	3.00E-17
comp59_c0_seq1	CUFF.83945	871.90	95.70	actin [Opisthacanthus cayaporum]	6.00E-102
comp62_c0_seq1	CUFF.88153	872.33	95.70	actin [Opisthacanthus cayaporum]	7.00E-102
comp83_c0_seq1	CUFF.107390	289.24	49.16	NADH dehydrogenase subunit 2 [Mesobuthus martensii]	5.00E-48
comp90_c0_seq1	CUFF.112754	954.34	58.18	hypothetical protein [Opisthacanthus cayaporum]	5.00E-14
comp92_c0_seq1	CUFF.114315	165.59	59.38	hypothetical protein [Opisthacanthus cayaporum]	4.00E-06
comp96_c0_seq1	CUFF.117587	997.09	58.90	hypothetical protein [Opisthacanthus cayaporum]	9.00E-21
comp104_c0_seq1	CUFF.3490	160.54	59.38	hypothetical protein [Opisthacanthus cayaporum]	4.00E-06
comp104_c0_seq2	CUFF.3488	144.13	59.38	hypothetical protein [Opisthacanthus cayaporum]	3.00E-06
comp111_c0_seq1	CUFF.8015	930.33	58.90	hypothetical protein [Opisthacanthus cayaporum]	9.00E-21
comp119_c0_seq1	CUFF.12237	910.88	58.18	hypothetical protein [Opisthacanthus cayaporum]	5.00E-14
comp144_c0_seq1	CUFF.24037	643.09	53.80	hypothetical protein [Opisthacanthus cayaporum]	4.00E-48
comp153_c0_seq1	CUFF.26759	278.26	68.49	NADH dehydrogenase-like protein [Opisthacanthus cayaporum]	2.00E-16
comp156_c0_seq1	CUFF.27449	640.19	59.09	ferritin-like peptide [Opisthacanthus cayaporum]	3.00E-74
comp157_c0_seq1	CUFF.27723	72.59	62.24	NADH dehydrogenase subunit 1 [Mesobuthus martensii]	1.00E-92
comp160_c0_seq1	CUFF.28670	639.31	53.80	hypothetical protein [Opisthacanthus cayaporum]	4.00E-48
comp167_c0_seq1	CUFF.30055	284.37	68.49	NADH dehydrogenase-like protein [Opisthacanthus cayaporum]	2.00E-16
comp169_c0_seq1	CUFF.30927	635.60	59.09	ferritin-like peptide [Opisthacanthus cayaporum]	3.00E-74
comp177_c1_seq1	CUFF.32502	552.48	67.26	hypothetical protein [Opisthacanthus cayaporum]	9.00E-31
comp182_c0_seq1	CUFF.33592	71.52	62.24	NADH dehydrogenase subunit 1 [Mesobuthus martensii]	1.00E-92
comp192_c0_seq1	CUFF.35458	16.09	62.96	antimicrobial peptide NDPB 5.7 precursor [Opisthacanthus cayaporum]	1.00E-13
comp192_c0_seq2	CUFF.35456	8.37	62.96	antimicrobial peptide NDPB 5.7 precursor [Opisthacanthus cayaporum]	1.00E-13
comp192_c0_seq4	CUFF.35462	16.75	62.96	antimicrobial peptide NDPB 5.7 precursor [Opisthacanthus cayaporum]	1.00E-13
comp192_c0_seq5	CUFF.35469	18.50	62.96	antimicrobial peptide NDPB 5.7 precursor [Opisthacanthus cayaporum]	1.00E-13

comp192_c0_seq7	CUFF.35464	34.89	62.96	antimicrobial peptide NDPB 5.7 precursor [Opisthacanthus cayaporum]	1.00E-13
comp192_c0_seq8	CUFF.35471	9.23	62.96	antimicrobial peptide NDPB 5.7 precursor [Opisthacanthus cayaporum]	1.00E-13
comp192_c0_seq9	CUFF.35477	12.64	62.96	antimicrobial peptide NDPB 5.7 precursor [Opisthacanthus cayaporum]	9.00E-14
comp194_c0_seq1	CUFF.35989	623.26	67.26	hypothetical protein [Opisthacanthus cayaporum]	1.00E-30
comp196_c0_seq1	CUFF.36065	8.48	62.96	antimicrobial peptide NDPB 5.7 precursor [Opisthacanthus cayaporum]	1.00E-13
comp196_c0_seq2	CUFF.36061	8.36	62.96	antimicrobial peptide NDPB 5.7 precursor [Opisthacanthus cayaporum]	1.00E-13
comp196_c0_seq3	CUFF.36063	9.52	62.96	antimicrobial peptide NDPB 5.7 precursor [Opisthacanthus cayaporum]	1.00E-13
comp196_c0_seq4	CUFF.36073	11.60	62.96	antimicrobial peptide NDPB 5.7 precursor [Opisthacanthus cayaporum]	1.00E-13
comp196_c0_seq5	CUFF.36067	33.80	62.96	antimicrobial peptide NDPB 5.7 precursor [Opisthacanthus cayaporum]	1.00E-13
comp196_c0_seq6	CUFF.36069	9.22	62.96	antimicrobial peptide NDPB 5.7 precursor [Opisthacanthus cayaporum]	1.00E-13
comp196_c0_seq7	CUFF.36079	12.51	62.96	antimicrobial peptide NDPB 5.7 precursor [Opisthacanthus cayaporum]	9.00E-14
comp202_c0_seq1	CUFF.37153	537.93	51.25	Full=Bradykinin-potentiating peptide-like; AltName: Short=NDBP-3.7	3.00E-07
comp210_c0_seq1	CUFF.38350	549.25	51.25	Full=Bradykinin-potentiating peptide-like; AltName: Short=NDBP-3.7	3.00E-07
comp221_c0_seq1	CUFF.39758	135.71	49.00	venom toxin [Opisthacanthus cayaporum]	5.00E-34
comp221_c0_seq2	CUFF.39776	201.31	52.36	venom toxin [Opisthacanthus cayaporum]	4.00E-37
comp231_c0_seq1	CUFF.41013	317.69	46.30	anti-microbial peptide-like protein [Mesobuthus martensii]	1.00E-06
comp234_c0_seq1	CUFF.41380	520.39	46.30	anti-microbial peptide-like protein [Mesobuthus martensii]	1.00E-06
comp233_c0_seq1	CUFF.41248	129.59	48.50	venom toxin [Opisthacanthus cayaporum]	2.00E-33
comp233_c0_seq2	CUFF.41260	199.74	51.31	venom toxin [Opisthacanthus cayaporum]	3.00E-36
comp248_c0_seq1	CUFF.43286	198.21	100.00	calmodulin-like protein [Opisthacanthus cayaporum]	9.00E-15
comp249_c0_seq1	CUFF.43391	256.27	72.73	calmodulin-like protein [Opisthacanthus cayaporum]	1.00E-10
comp262_c0_seq1	CUFF.45047	197.52	100.00	calmodulin-like protein [Opisthacanthus cayaporum]	9.00E-15
comp278_c0_seq1	CUFF.47180	257.19	72.73	calmodulin-like protein [Opisthacanthus cayaporum]	1.00E-10
comp299_c0_seq1	CUFF.49672	165.46	60.00	venom toxin [Opisthacanthus cayaporum]	6.00E-29
comp311_c0_seq1	CUFF.51433	142.70	25.23	elastase-like protein [Opisthacanthus cayaporum]	4.00E-17
comp324_c0_seq1	CUFF.52980	347.40	59.30	Full=Hg-scorpine-like 2; Short=HgeScplp2; Short=Hgscplike2;	3.00E-27
comp330_c0_seq1	CUFF.53511	555.36	52.70	putative toxin [Opisthacanthus cayaporum]	2.00E-18
comp336_c0_seq1	CUFF.54303	353.87	59.30	Full=Hg-scorpine-like 2; Short=HgeScplp2; Short=Hgscplike2;	3.00E-27
comp339_c0_seq1	CUFF.54685	287.85	95.00	60s ribosomal protein L10 [Tityus discrepans]	1.00E-127
comp348_c0_seq1	CUFF.55826	169.89	60.00	venom toxin [Opisthacanthus cayaporum]	5.00E-29
comp355_c0_seq1	CUFF.56766	239.63	63.89	ferritin-like peptide [Opisthacanthus cayaporum]	1.00E-54

comp366_c0_seq1	CUFF.58374	205.08	27.38	putative RNA binding protein [Opisthacanthus cayaporum]	1.00E-06
comp372_c0_seq1	CUFF.58728	239.63	63.89	ferritin-like peptide [Opisthacanthus cayaporum]	1.00E-54
comp374_c0_seq1	CUFF.58972	137.71	25.23	elastase-like protein [Opisthacanthus cayaporum]	4.00E-17
comp380_c0_seq1	CUFF.59613	178.61	55.56	venom toxin [Opisthacanthus cayaporum]	3.00E-93
comp395_c0_seq1	CUFF.61350	200.03	52.70	putative toxin [Opisthacanthus cayaporum]	2.00E-18
comp397_c0_seq1	CUFF.61693	178.46	55.56	venom toxin [Opisthacanthus cayaporum]	3.00E-93
comp398_c0_seq1	CUFF.61807	290.63	95.00	60s ribosomal protein L10 [Tityus discrepans]	1.00E-127
comp417_c0_seq1	CUFF.63986	205.10	27.38	putative RNA binding protein [Opisthacanthus cayaporum]	1.00E-06
comp456_c0_seq1	CUFF.68239	28.72	24.42	putative RNA binding protein [Opisthacanthus cayaporum]	3.00E-05
comp456_c0_seq2	CUFF.68355	41.31	24.42	putative RNA binding protein [Opisthacanthus cayaporum]	2.00E-05
comp456_c0_seq3	CUFF.68169	35.69	24.42	putative RNA binding protein [Opisthacanthus cayaporum]	2.00E-05
comp456_c0_seq4	CUFF.68277	52.98	24.42	putative RNA binding protein [Opisthacanthus cayaporum]	2.00E-05
comp492_c0_seq1	CUFF.72495	27.56	24.42	putative RNA binding protein [Opisthacanthus cayaporum]	2.00E-05
comp492_c0_seq2	CUFF.72572	42.70	24.42	putative RNA binding protein [Opisthacanthus cayaporum]	2.00E-05
comp492_c0_seq3	CUFF.72417	35.72	24.42	putative RNA binding protein [Opisthacanthus cayaporum]	2.00E-05
comp492_c0_seq4	CUFF.72483	53.00	24.42	putative RNA binding protein [Opisthacanthus cayaporum]	2.00E-05
comp588_c0_seq1	CUFF.82902	258.69	53.16	beta-KTx-like peptide [Hadrurus gertschi]	4.00E-19
comp596_c0_seq1	CUFF.83730	271.52	53.16	beta-KTx-like peptide [Hadrurus gertschi]	3.00E-19
comp606_c0_seq1	CUFF.84813	59.90	24.56	elastase-like protein [Opisthacanthus cayaporum]	9.00E-10
comp644_c0_seq1	CUFF.88771	113.96	24.56	elastase-like protein [Opisthacanthus cayaporum]	9.00E-10
comp664_c0_seq1	CUFF.91005	183.23	83.85	ATP synthase-like protein [Opisthacanthus cayaporum]	8.00E-43
comp676_c0_seq1	CUFF.92185	182.82	83.85	ATP synthase-like protein [Opisthacanthus cayaporum]	8.00E-43
comp687_c0_seq1	CUFF.93362	50.48	27.78	putative RNA binding protein [Opisthacanthus cayaporum]	4.00E-05
comp687_c0_seq2	CUFF.93394	63.27	27.78	putative RNA binding protein [Opisthacanthus cayaporum]	4.00E-05
comp688_c0_seq1	CUFF.93395	50.53	27.78	putative RNA binding protein [Opisthacanthus cayaporum]	4.00E-05
comp688_c0_seq2	CUFF.93397	63.27	27.78	putative RNA binding protein [Opisthacanthus cayaporum]	4.00E-05
comp709_c0_seq1	CUFF.95437	107.16	48.15	calmodulin-like protein [Opisthacanthus cayaporum]	2.00E-05
comp727_c0_seq1	CUFF.96963	77.67	43.66	hypothetical protein [Tityus discrepans]	4.00E-14
comp727_c0_seq2	CUFF.96973	93.46	43.06	hypothetical protein [Tityus discrepans]	1.00E-14
comp744_c0_seq1	CUFF.98512	77.99	43.66	hypothetical protein [Tityus discrepans]	4.00E-14
comp744_c0_seq2	CUFF.98522	93.95	43.06	hypothetical protein [Tityus discrepans]	1.00E-14

comp753_c0_seq1	CUFF.99467	93.35	32.95	serpin peptidase inhibitor-like protein [Opisthacanthus cayaporum]	5.00E-22
comp762_c0_seq1	CUFF.100279	109.45	48.15	calmodulin-like protein [Opisthacanthus cayaporum]	2.00E-05
comp837_c0_seq1	CUFF.106859	230.32	97.14	60S ribosomal protein L38 [Tityus discrepans]	2.00E-35
comp843_c0_seq1	CUFF.107707	100.50	54.26	venom toxin [Opisthacanthus cayaporum]	4.00E-57
comp847_c0_seq1	CUFF.108121	100.01	54.26	venom toxin [Opisthacanthus cayaporum]	4.00E-57
comp849_c0_seq1	CUFF.107954	72.15	50.00	putative potassium-channel inhibitor Kcug2 precursor [Mesobuthus martensii]	1.00E-09
comp849_c0_seq2	CUFF.107952	8.40	43.33	RecName: Full=Toxin OcyKTx2	1.00E-05
comp849_c0_seq3	CUFF.107956	10.21	58.06	RecName: Full=Toxin OcyKTx2	7.00E-09
comp849_c0_seq4	CUFF.107957	6.72	61.29	RecName: Full=Toxin OcyKTx2	2.00E-10
comp849_c0_seq5	CUFF.107972	46.05	51.52	Chain A, Scorpion Toxin Bmtx1 From Buthus Martensii Karsch, Nmr, 25 Structures	1.00E-09
comp849_c0_seq6	CUFF.107966	25.57	55.56	neurotoxin TX1 precursor [Mesobuthus martensii]	4.00E-11
comp849_c0_seq7	CUFF.107960	8.53	43.33	RecName: Full=Toxin OcyKTx2	9.00E-06
comp849_c0_seq8	CUFF.107964	11.82	58.06	RecName: Full=Toxin OcyKTx2	5.00E-09
comp849_c0_seq9	CUFF.107962	7.86	54.05	neurotoxin TX1 precursor [Mesobuthus martensii]	1.00E-10
comp849_c0_seq10	CUFF.107946	372.63	51.52	RecName: Full=Potassium channel toxin alpha-KTx 4.3; AltName: Full=Toxin TdK1	2.00E-09
comp871_c0_seq1	CUFF.109760	230.07	97.14	60S ribosomal protein L38 [Tityus discrepans]	3.00E-35
comp874_c0_seq1	CUFF.110135	93.35	32.95	serpin peptidase inhibitor-like protein [Opisthacanthus cayaporum]	5.00E-22
comp903_c0_seq1	CUFF.112449	91.18	26.03	putative RNA binding protein [Opisthacanthus cayaporum]	8.00E-05
comp916_c0_seq1	CUFF.113537	91.19	26.03	putative RNA binding protein [Opisthacanthus cayaporum]	6.00E-05
comp928_c0_seq2	CUFF.114314	51.60	26.44	putative RNA binding protein [Opisthacanthus cayaporum]	8.00E-06
comp941_c0_seq3	CUFF.115246	80.49	26.44	putative RNA binding protein [Opisthacanthus cayaporum]	4.00E-06
comp961_c0_seq1	CUFF.116971	86.87	49.64	ferritin-like peptide [Opisthacanthus cayaporum]	1.00E-34
comp986_c0_seq1	CUFF.118789	86.42	49.64	ferritin-like peptide [Opisthacanthus cayaporum]	1.00E-34
comp990_c0_seq1	CUFF.119142	62.29	100.00	hypothetical protein [Tityus discrepans]	5.00E-12
comp1048_c0_seq1	CUFF.3513	62.20	100.00	hypothetical protein [Tityus discrepans]	5.00E-12
comp1069_c0_seq1	CUFF.4661	31.36	55.56	neurotoxin TX1 precursor [Mesobuthus martensii]	7.00E-11
comp1069_c0_seq2	CUFF.4659	4.83	43.33	RecName: Full=Toxin OcyKTx2	2.00E-05
comp1069_c0_seq3	CUFF.4669	72.15	50.00	putative potassium-channel inhibitor Kcug2 precursor [Mesobuthus martensii]	1.00E-09
comp1069_c0_seq4	CUFF.4663	4.16	54.05	neurotoxin TX1 precursor [Mesobuthus martensii]	2.00E-10
comp1069_c0_seq5	CUFF.4665	10.21	58.06	RecName: Full=Toxin OcyKTx2	6.00E-09
comp1136_c0_seq1	CUFF.8810	150.06	50.00	RecName: Full=Tddefensin	3.00E-08

comp1181_c0_seq1	CUFF.11378	151.87	50.00	RecName: Full=Tddefensin	3.00E-08
comp1231_c0_seq1	CUFF.13972	77.73	55.17	calmodulin-like protein [Opisthacanthus cayaporum]	6.00E-05
comp1236_c0_seq1	CUFF.14107	129.54	72.00	hypothetical protein [Opisthacanthus cayaporum]	1.00E-21
comp1239_c0_seq1	CUFF.14349	83.70	77.59	serpin peptidase inhibitor-like protein [Opisthacanthus cayaporum]	2.00E-72
comp1249_c0_seq1	CUFF.14908	119.86	59.68	phospholipase-like protein [Opisthacanthus cayaporum]	5.00E-20
comp1250_c0_seq1	CUFF.15016	85.21	77.59	serpin peptidase inhibitor-like protein [Opisthacanthus cayaporum]	2.00E-72
comp1267_c0_seq1	CUFF.15841	100.75	74.07	putative antimicrobial peptide precursor [Opisthacanthus cayaporum]	1.00E-18
comp1321_c0_seq1	CUFF.18376	80.30	55.17	calmodulin-like protein [Opisthacanthus cayaporum]	5.00E-05
comp1393_c0_seq1	CUFF.21307	91.69	74.07	putative antimicrobial peptide precursor [Opisthacanthus cayaporum]	1.00E-18
comp1409_c0_seq2	CUFF.21814	13.52	72.00	hypothetical protein [Opisthacanthus cayaporum]	1.00E-21
comp1409_c0_seq3	CUFF.21812	27.52	72.00	hypothetical protein [Opisthacanthus cayaporum]	1.00E-21
comp1418_c0_seq1	CUFF.22227	127.27	59.68	phospholipase-like protein [Opisthacanthus cayaporum]	5.00E-20
comp1431_c0_seq1	CUFF.22690	57.34	34.16	elastase-like protein [Opisthacanthus cayaporum]	5.00E-26
comp1490_c0_seq1	CUFF.24848	26.70	74.89	cytochrome oxidase subunit I [Mesobuthus martensii]	5.00E-67
comp1498_c0_seq1	CUFF.25129	117.78	51.19	cytochrome c oxidase subunit I [Mesobuthus martensii]	1.00E-22
comp1513_c0_seq1	CUFF.25577	137.36	96.00	60S ribosomal protein L29 [Tityus discrepans]	9.00E-27
comp1538_c0_seq1	CUFF.26244	35.21	49.22	sulfotransferase-like protein [Opisthacanthus cayaporum]	4.00E-58
comp1538_c0_seq2	CUFF.26269	44.08	49.22	sulfotransferase-like protein [Opisthacanthus cayaporum]	3.00E-58
comp1553_c0_seq1	CUFF.26754	38.18	49.22	sulfotransferase-like protein [Opisthacanthus cayaporum]	4.00E-58
comp1589_c0_seq1	CUFF.27758	77.76	34.00	elastase-like protein [Opisthacanthus cayaporum]	4.00E-25
comp1612_c0_seq1	CUFF.28403	47.31	48.70	sulfotransferase-like protein [Opisthacanthus cayaporum]	2.00E-56
comp1678_c0_seq1	CUFF.29967	76.28	32.47	putative RNA binding protein [Opisthacanthus cayaporum]	4.00E-09
comp1698_c0_seq1	CUFF.30466	46.89	48.70	sulfotransferase-like protein [Opisthacanthus cayaporum]	1.00E-56
comp1699_c0_seq1	CUFF.30464	134.67	96.00	60S ribosomal protein L29 [Tityus discrepans]	9.00E-27
comp1735_c0_seq1	CUFF.31368	31.45	31.61	venom toxin [Opisthacanthus cayaporum]	2.00E-17
comp1747_c0_seq1	CUFF.31640	42.67	34.66	serpin peptidase inhibitor-like protein [Opisthacanthus cayaporum]	2.00E-24
comp1749_c0_seq1	CUFF.31708	23.22	31.43	putative RNA binding protein [Opisthacanthus cayaporum]	8.00E-07
comp1749_c0_seq2	CUFF.31695	31.17	31.43	putative RNA binding protein [Opisthacanthus cayaporum]	5.00E-07
comp1777_c0_seq1	CUFF.32243	23.20	31.43	putative RNA binding protein [Opisthacanthus cayaporum]	8.00E-07
comp1777_c0_seq2	CUFF.32251	31.20	31.43	putative RNA binding protein [Opisthacanthus cayaporum]	5.00E-07
comp1820_c0_seq1	CUFF.33154	5.96	36.92	hypothetical protein [Tityus discrepans]	3.00E-08

comp1820_c0_seq2	CUFF.33158	7.02	36.92	hypothetical protein [Tityus discrepans]	3.00E-08
comp1828_c0_seq1	CUFF.33343	76.25	32.47	putative RNA binding protein [Opisthacanthus cayaporum]	4.00E-09
comp1871_c0_seq1	CUFF.34251	6.20	36.92	hypothetical protein [Tityus discrepans]	3.00E-08
comp1902_c0_seq1	CUFF.34883	31.12	31.61	venom toxin [Opisthacanthus cayaporum]	2.00E-17
comp1909_c0_seq1	CUFF.35003	45.02	27.33	serpin peptidase inhibitor-like protein [Opisthacanthus cayaporum]	5.00E-13
comp1933_c0_seq1	CUFF.35537	72.82	37.18	hypothetical protein [Tityus discrepans]	6.00E-12
comp1935_c0_seq1	CUFF.35565	63.16	37.18	hypothetical protein [Tityus discrepans]	7.00E-12
comp1955_c0_seq1	CUFF.35896	43.32	34.09	serpin peptidase inhibitor-like protein [Opisthacanthus cayaporum]	8.00E-24
comp1959_c0_seq1	CUFF.35914	32.46	92.00	hypothetical protein [Opisthacanthus cayaporum]	1.00E-22
comp1991_c0_seq1	CUFF.36407	99.63	46.51	potassium channel toxin-like peptide [Mesobuthus martensii]	7.00E-09
comp1995_c0_seq1	CUFF.36512	48.02	27.33	serpin peptidase inhibitor-like protein [Opisthacanthus cayaporum]	5.00E-13
comp2036_c0_seq1	CUFF.37104	19.55	42.76	ATP synthase F0 subunit 6 [Mesobuthus martensii]	4.00E-19
comp2036_c0_seq2	CUFF.37094	13.25	58.33	cytochrome c oxidase subunit III [Mesobuthus martensii]	3.00E-64
comp2036_c0_seq3	CUFF.37090	6.40	58.06	cytochrome c oxidase subunit III [Mesobuthus martensii]	1.00E-63
comp2060_c0_seq1	CUFF.37463	31.82	92.00	hypothetical protein [Opisthacanthus cayaporum]	1.00E-22
comp2092_c0_seq1	CUFF.37983	118.76	39.06	potassium channel toxin-like peptide [Mesobuthus martensii]	5.00E-08
comp2100_c0_seq1	CUFF.38119	99.63	46.51	potassium channel toxin-like peptide [Mesobuthus martensii]	7.00E-09
comp2168_c0_seq1	CUFF.39033	119.75	39.06	potassium channel toxin-like peptide [Mesobuthus martensii]	5.00E-08
comp2227_c0_seq1	CUFF.39785	28.45	44.62	RecName: Full=Protease inhibitor Hg1; Flags: Precursor	7.00E-13
comp2280_c0_seq1	CUFF.40536	43.96	28.57	serpin peptidase inhibitor-like protein [Opisthacanthus cayaporum]	7.00E-18
comp2324_c0_seq1	CUFF.41087	19.45	42.76	ATP synthase F0 subunit 6 [Mesobuthus martensii]	4.00E-19
comp2324_c0_seq2	CUFF.41079	13.25	58.33	cytochrome c oxidase subunit III [Mesobuthus martensii]	3.00E-64
comp2324_c0_seq3	CUFF.41075	6.46	57.20	cytochrome c oxidase subunit III [Mesobuthus martensii]	7.00E-59
comp2393_c0_seq1	CUFF.41949	11.86	29.03	venom toxin [Opisthacanthus cayaporum]	2.00E-05
comp2443_c0_seq1	CUFF.42567	28.92	44.62	RecName: Full=Protease inhibitor Hg1; Flags: Precursor	7.00E-13
comp2485_c0_seq1	CUFF.43113	44.43	28.57	serpin peptidase inhibitor-like protein [Opisthacanthus cayaporum]	7.00E-18
comp2519_c0_seq1	CUFF.43524	11.91	29.03	venom toxin [Opisthacanthus cayaporum]	2.00E-05
comp2564_c0_seq1	CUFF.44139	18.56	30.00	putative RNA binding protein [Opisthacanthus cayaporum]	3.00E-06
comp2642_c0_seq1	CUFF.45111	57.49	38.71	putative RNA binding protein [Opisthacanthus cayaporum]	5.00E-65
comp2885_c0_seq1	CUFF.48173	18.56	34.64	serpin peptidase inhibitor-like protein [Opisthacanthus cayaporum]	7.00E-15
comp2885_c0_seq2	CUFF.48171	14.56	34.64	serpin peptidase inhibitor-like protein [Opisthacanthus cayaporum]	7.00E-15

comp2885_c0_seq3	CUFF.48169	12.71	36.14	serpin peptidase inhibitor-like protein [Opisthacanthus cayaporum]	2.00E-19
comp2885_c0_seq4	CUFF.48193	14.91	36.14	serpin peptidase inhibitor-like protein [Opisthacanthus cayaporum]	2.00E-19
comp2960_c0_seq1	CUFF.49176	18.20	32.26	venom peptide BmKAPi precursor [Mesobuthus martensii]	4.00E-06
comp2960_c0_seq2	CUFF.49182	55.34	31.18	venom peptide BmKAPi precursor [Mesobuthus martensii]	6.00E-06
comp2962_c0_seq1	CUFF.49199	4.65	58.82	coiled-coil-helix-coiled-coil-helix domain-like protein [Opisthacanthus cayaporum]	1.00E-11
comp2962_c0_seq2	CUFF.49205	5.69	94.12	coiled-coil-helix-coiled-coil-helix domain-like protein [Opisthacanthus cayaporum]	1.00E-17
comp2962_c0_seq3	CUFF.49207	6.51	69.49	coiled-coil-helix-coiled-coil-helix domain-like protein [Opisthacanthus cayaporum]	1.00E-19
comp2962_c0_seq4	CUFF.49212	50.10	89.13	coiled-coil-helix-coiled-coil-helix domain-like protein [Opisthacanthus cayaporum]	2.00E-22
comp2965_c0_seq1	CUFF.49245	265.26	33.33	putative potassium-channel inhibitor Kcug2 precursor [Mesobuthus martensii]	2.00E-06
comp2971_c0_seq1	CUFF.49322	38.81	37.29	venom peptide 2 precursor [Mesobuthus martensii]	2.00E-06
comp2981_c0_seq1	CUFF.49404	265.26	33.33	putative potassium-channel inhibitor Kcug2 precursor [Mesobuthus martensii]	2.00E-06
comp3005_c0_seq1	CUFF.49726	14.66	45.13	sulfotransferase-like protein [Opisthacanthus cayaporum]	6.00E-53
comp3005_c0_seq2	CUFF.49730	12.58	47.42	sulfotransferase-like protein [Opisthacanthus cayaporum]	3.00E-54
comp3016_c0_seq1	CUFF.49838	54.96	31.18	venom peptide BmKAPi precursor [Mesobuthus martensii]	7.00E-06
comp3016_c0_seq2	CUFF.49844	18.03	32.26	venom peptide BmKAPi precursor [Mesobuthus martensii]	4.00E-06
comp3041_c0_seq1	CUFF.50167	57.49	38.71	putative RNA binding protein [Opisthacanthus cayaporum]	5.00E-65
comp3056_c0_seq1	CUFF.50333	27.46	45.75	ATP synthase F0 subunit 6 [Mesobuthus martensii]	1.00E-21
comp3064_c0_seq1	CUFF.50422	27.18	45.75	ATP synthase F0 subunit 6 [Mesobuthus martensii]	1.00E-21
comp3093_c0_seq1	CUFF.50818	47.66	29.58	TXLP4 [Mesobuthus martensii]	8.00E-08
comp3106_c0_seq1	CUFF.50949	38.61	37.29	venom peptide 2 precursor [Mesobuthus martensii]	2.00E-06
comp3117_c0_seq1	CUFF.51161	18.01	34.64	serpin peptidase inhibitor-like protein [Opisthacanthus cayaporum]	7.00E-15
comp3126_c0_seq1	CUFF.51272	8.98	88.72	phospholipase C-like protein [Opisthacanthus cayaporum]	3.00E-100
comp3187_c0_seq1	CUFF.52105	14.77	36.14	serpin peptidase inhibitor-like protein [Opisthacanthus cayaporum]	2.00E-19
comp3223_c0_seq1	CUFF.52575	7.59	27.91	putative RNA binding protein [Opisthacanthus cayaporum]	5.00E-07
comp3223_c0_seq2	CUFF.52560	6.86	27.91	putative RNA binding protein [Opisthacanthus cayaporum]	5.00E-07
comp3223_c0_seq3	CUFF.52538	12.05	27.91	putative RNA binding protein [Opisthacanthus cayaporum]	2.00E-07
comp3223_c0_seq4	CUFF.52576	9.94	27.91	putative RNA binding protein [Opisthacanthus cayaporum]	2.00E-07
comp3229_c0_seq2	CUFF.52655	18.58	26.44	putative RNA binding protein [Opisthacanthus cayaporum]	8.00E-06
comp3266_c0_seq1	CUFF.53071	18.89	26.44	putative RNA binding protein [Opisthacanthus cayaporum]	8.00E-06
comp3287_c0_seq1	CUFF.53342	9.06	88.72	phospholipase C-like protein [Opisthacanthus cayaporum]	3.00E-100
comp3287_c0_seq2	CUFF.53333	9.37	88.72	phospholipase C-like protein [Opisthacanthus cayaporum]	3.00E-100

comp3361_c0_seq1	CUFF.54115	14.46	45.13	sulfotransferase-like protein [Opisthacanthus cayaporum]	6.00E-53
comp3361_c0_seq2	CUFF.54109	9.74	45.13	sulfotransferase-like protein [Opisthacanthus cayaporum]	4.00E-53
comp3375_c0_seq1	CUFF.54305	53.07	38.81	putative secreted protein [Opisthacanthus cayaporum]	9.00E-11
comp3398_c0_seq1	CUFF.54612	8.42	33.72	putative RNA binding protein [Opisthacanthus cayaporum]	4.00E-07
comp3398_c0_seq2	CUFF.54606	7.91	34.15	putative RNA binding protein [Opisthacanthus cayaporum]	3.00E-08
comp3398_c0_seq3	CUFF.54600	6.48	33.72	putative RNA binding protein [Opisthacanthus cayaporum]	2.00E-07
comp3398_c0_seq4	CUFF.54602	5.43	34.15	putative RNA binding protein [Opisthacanthus cayaporum]	2.00E-08
comp3419_c0_seq1	CUFF.54851	7.30	27.91	putative RNA binding protein [Opisthacanthus cayaporum]	5.00E-07
comp3419_c0_seq2	CUFF.54849	6.57	27.91	putative RNA binding protein [Opisthacanthus cayaporum]	5.00E-07
comp3419_c0_seq3	CUFF.54855	12.07	27.91	putative RNA binding protein [Opisthacanthus cayaporum]	2.00E-07
comp3419_c0_seq4	CUFF.54857	9.98	27.91	putative RNA binding protein [Opisthacanthus cayaporum]	2.00E-07
comp3435_c0_seq1	CUFF.55046	46.19	29.58	TXLP4 [Mesobuthus martensii]	8.00E-08
comp3521_c0_seq1	CUFF.56169	9.40	33.72	putative RNA binding protein [Opisthacanthus cayaporum]	4.00E-07
comp3521_c0_seq2	CUFF.56167	8.85	34.15	putative RNA binding protein [Opisthacanthus cayaporum]	4.00E-08
comp3521_c0_seq3	CUFF.56135	6.22	33.72	putative RNA binding protein [Opisthacanthus cayaporum]	2.00E-07
comp3521_c0_seq4	CUFF.56151	5.23	34.15	putative RNA binding protein [Opisthacanthus cayaporum]	2.00E-08
comp3616_c0_seq1	CUFF.57204	4.34	58.82	coiled-coil-helix-coiled-coil-helix domain-like protein [Opisthacanthus cayaporum]	2.00E-11
comp3616_c0_seq2	CUFF.57208	6.39	94.12	coiled-coil-helix-coiled-coil-helix domain-like protein [Opisthacanthus cayaporum]	1.00E-17
comp3616_c0_seq3	CUFF.57214	7.38	69.49	coiled-coil-helix-coiled-coil-helix domain-like protein [Opisthacanthus cayaporum]	1.00E-19
comp3616_c0_seq4	CUFF.57224	46.20	89.13	coiled-coil-helix-coiled-coil-helix domain-like protein [Opisthacanthus cayaporum]	2.00E-22
comp3687_c0_seq1	CUFF.58078	7.82	39.68	venom toxin [Opisthacanthus cayaporum]	8.00E-09
comp3687_c0_seq2	CUFF.58084	13.29	39.68	venom toxin [Opisthacanthus cayaporum]	7.00E-09
comp3700_c0_seq1	CUFF.58196	19.29	32.76	potassium channel toxin-like peptide [Mesobuthus martensii]	2.00E-05
comp3707_c0_seq1	CUFF.58287	18.22	42.93	NADH dehydrogenase subunit 4 [Mesobuthus martensii]	6.00E-28
comp3747_c0_seq1	CUFF.58762	18.67	30.00	putative RNA binding protein [Opisthacanthus cayaporum]	3.00E-06
comp3813_c0_seq1	CUFF.59529	50.33	60.66	putative antimicrobial peptide [Opisthacanthus cayaporum]	7.00E-09
comp3842_c0_seq1	CUFF.59921	4.80	41.94	venom peptide BmKAPi precursor [Mesobuthus martensii]	5.00E-06
comp3842_c0_seq3	CUFF.59927	23.98	36.78	venom peptide BmKAPi precursor [Mesobuthus martensii]	6.00E-07
comp3859_c0_seq1	CUFF.60247	17.94	46.28	NADH dehydrogenase subunit 1 [Mesobuthus martensii]	4.00E-60
comp3859_c0_seq2	CUFF.60235	14.90	46.56	NADH dehydrogenase subunit 1 [Mesobuthus martensii]	5.00E-58
comp3935_c0_seq1	CUFF.61074	50.00	60.66	putative antimicrobial peptide [Opisthacanthus cayaporum]	7.00E-09

comp4004_c0_seq1	CUFF.61832	29.73	75.00	putative transport protein [Opisthacanthus cayaporum]	2.00E-25
comp4015_c0_seq1	CUFF.61936	8.30	100.00	cytochrome oxidase subunit I [Mesobuthus martensii]	3.00E-24
comp4029_c0_seq1	CUFF.62151	27.50	27.81	venom toxin [Opisthacanthus cayaporum]	5.00E-16
comp4048_c0_seq1	CUFF.62342	25.98	39.29	venom peptide BmKAPi precursor [Mesobuthus martensii]	5.00E-08
comp4050_c0_seq1	CUFF.62356	19.42	32.76	potassium channel toxin-like peptide [Mesobuthus martensii]	2.00E-05
comp4101_c0_seq1	CUFF.62975	33.66	50.00	cytochrome c oxidase subunit II [Mesobuthus martensii]	1.00E-38
comp4102_c0_seq1	CUFF.62980	52.20	38.81	putative secreted protein [Opisthacanthus cayaporum]	9.00E-11
comp4124_c0_seq1	CUFF.63189	10.98	39.69	NADH dehydrogenase subunit 4 [Mesobuthus martensii]	4.00E-39
comp4162_c0_seq1	CUFF.63657	13.47	27.18	putative RNA binding protein [Opisthacanthus cayaporum]	2.00E-06
comp4167_c0_seq1	CUFF.63696	5.39	39.68	venom toxin [Opisthacanthus cayaporum]	8.00E-09
comp4167_c0_seq2	CUFF.63698	27.82	39.68	venom toxin [Opisthacanthus cayaporum]	7.00E-09
comp4170_c0_seq1	CUFF.63719	27.50	27.81	venom toxin [Opisthacanthus cayaporum]	5.00E-16
comp4212_c0_seq1	CUFF.64175	16.11	35.48	putative secreted protein [Opisthacanthus cayaporum]	5.00E-08
comp4212_c0_seq2	CUFF.64165	10.25	31.25	putative secreted protein [Opisthacanthus cayaporum]	3.00E-08
comp4236_c0_seq1	CUFF.64441	13.38	27.18	putative RNA binding protein [Opisthacanthus cayaporum]	2.00E-06
comp4259_c0_seq1	CUFF.64661	22.44	39.29	venom peptide BmKAPi precursor [Mesobuthus martensii]	6.00E-08
comp4317_c0_seq1	CUFF.65328	20.00	54.37	cytochrome b [Mesobuthus martensii]	2.00E-66
comp4334_c0_seq1	CUFF.65516	21.82	50.23	cytochrome c oxidase subunit II [Mesobuthus martensii]	4.00E-40
comp4338_c0_seq1	CUFF.65558	30.31	75.00	putative transport protein [Opisthacanthus cayaporum]	2.00E-25
comp4356_c0_seq1	CUFF.65795	25.50	45.24	putative anticoagulant peptide AP1 [Mesobuthus martensii]	4.00E-16
comp4361_c0_seq1	CUFF.65849	17.42	56.49	cytochrome b [Mesobuthus martensii]	6.00E-83
comp4369_c0_seq1	CUFF.65958	26.27	68.51	cytochome oxidase subunit I [Tityus discrepans]	1.00E-71
comp4393_c0_seq1	CUFF.66245	30.10	47.37	NADH dehydrogenase subunit 1 [Mesobuthus martensii]	9.00E-43
comp4410_c0_seq1	CUFF.66393	21.91	51.72	hypothetical protein [Opisthacanthus cayaporum]	2.00E-24
comp4509_c0_seq1	CUFF.67488	1.10	26.19	putative RNA binding protein [Opisthacanthus cayaporum]	3.00E-05
comp4509_c0_seq2	CUFF.67496	1.07	26.19	putative RNA binding protein [Opisthacanthus cayaporum]	3.00E-05
comp4509_c0_seq5	CUFF.67502	1.16	26.19	putative RNA binding protein [Opisthacanthus cayaporum]	3.00E-05
comp4509_c0_seq6	CUFF.67504	1.12	26.19	putative RNA binding protein [Opisthacanthus cayaporum]	3.00E-05
comp4536_c0_seq1	CUFF.67856	8.73	24.75	elastase-like protein [Opisthacanthus cayaporum]	8.00E-12
comp4549_c0_seq1	CUFF.67972	9.66	32.88	putative RNA binding protein [Opisthacanthus cayaporum]	3.00E-08
comp4605_c0_seq1	CUFF.68591	13.21	35.48	putative secreted protein [Opisthacanthus cayaporum]	5.00E-08

comp4605_c0_seq2	CUFF.68579	9.21	31.25	putative secreted protein [Opisthacanthus cayaporum]	3.00E-08
comp4658_c0_seq1	CUFF.69129	33.56	50.96	cytochrome c oxidase subunit II [Mesobuthus martensii]	3.00E-42
comp4670_c0_seq1	CUFF.69297	21.82	50.23	cytochrome c oxidase subunit II [Mesobuthus martensii]	4.00E-40
comp4725_c0_seq2			100.00	venom peptide T-U [Mesobuthus martensii]	9.00E-05
comp4725_c0_seq3			100.00	venom peptide T-U [Mesobuthus martensii]	9.00E-05
comp4735_c0_seq1	CUFF.70035	26.11	41.89	hypothetical protein [Tityus discrepans]	3.00E-15
comp4762_c0_seq1	CUFF.70393	21.82	51.72	hypothetical protein [Opisthacanthus cayaporum]	2.00E-24
comp4762_c0_seq2	CUFF.70385	11.62	53.85	hypothetical protein [Opisthacanthus cayaporum]	9.00E-24
comp4793_c0_seq1	CUFF.70742	18.61	56.00	putative secreted protein [Opisthacanthus cayaporum]	2.00E-35
comp4799_c0_seq1	CUFF.70790	8.79	24.75	elastase-like protein [Opisthacanthus cayaporum]	7.00E-12
comp4799_c0_seq2	CUFF.70792	8.97	24.75	elastase-like protein [Opisthacanthus cayaporum]	7.00E-12
comp4891_c0_seq1	CUFF.72021	8.80	32.88	putative RNA binding protein [Opisthacanthus cayaporum]	3.00E-08
comp4984_c0_seq1	CUFF.72990	29.28	41.89	hypothetical protein [Tityus discrepans]	3.00E-15
comp4994_c0_seq3			100.00	venom peptide T-U [Mesobuthus martensii]	9.00E-05
comp4994_c0_seq4			100.00	venom peptide T-U [Mesobuthus martensii]	9.00E-05
comp5045_c0_seq1	CUFF.73752	20.45	32.61	Full=Phospholipase A2; Short=HgPLA2	3.00E-23
comp5105_c0_seq1	CUFF.74356	11.51	31.17	putative RNA binding protein [Opisthacanthus cayaporum]	1.00E-06
comp5108_c0_seq1	CUFF.74364	22.37	31.67	putative RNA binding protein [Opisthacanthus cayaporum]	2.00E-06
comp5183_c0_seq1	CUFF.75192	16.53	28.32	putative RNA binding protein [Opisthacanthus cayaporum]	6.00E-06
comp5223_c0_seq1	CUFF.75630	16.51	28.32	putative RNA binding protein [Opisthacanthus cayaporum]	6.00E-06
comp5235_c0_seq1	CUFF.75733	1.15	26.19	putative RNA binding protein [Opisthacanthus cayaporum]	4.00E-05
comp5235_c0_seq2	CUFF.75735	1.11	26.19	putative RNA binding protein [Opisthacanthus cayaporum]	4.00E-05
comp5235_c0_seq3	CUFF.75737	1.20	26.19	putative RNA binding protein [Opisthacanthus cayaporum]	4.00E-05
comp5235_c0_seq4	CUFF.75739	1.16	26.19	putative RNA binding protein [Opisthacanthus cayaporum]	3.00E-05
comp5235_c0_seq5	CUFF.75741	1.27	26.73	putative RNA binding protein [Opisthacanthus cayaporum]	1.00E-05
comp5235_c0_seq6	CUFF.75743	1.34	26.73	putative RNA binding protein [Opisthacanthus cayaporum]	1.00E-05
comp5238_c0_seq1	CUFF.75775	11.48	31.17	putative RNA binding protein [Opisthacanthus cayaporum]	1.00E-06
comp5361_c0_seq1	CUFF.77256	20.15	32.61	Full=Phospholipase A2; Short=HgPLA2	3.00E-23
comp5516_c0_seq1	CUFF.78890	12.20	29.85	actin [Opisthacanthus cayaporum]	7.00E-21
comp5534_c0_seq1	CUFF.79010	25.11	45.24	putative anticoagulant peptide AP1 [Mesobuthus martensii]	4.00E-16
comp5600_c0_seq1	CUFF.79787	6.51	25.30	putative RNA binding protein [Opisthacanthus cayaporum]	2.00E-05

comp5600_c0_seq2	CUFF.79797	8.13	25.30	putative RNA binding protein [Opisthacanthus cayaporum]	2.00E-05
comp5618_c0_seq1	CUFF.79976	14.04	32.94	venom toxin [Opisthacanthus cayaporum]	3.00E-20
comp5618_c0_seq2	CUFF.79962	6.55	40.68	venom toxin [Opisthacanthus cayaporum]	2.00E-08
comp5638_c0_seq1	CUFF.80144	29.87	29.21	venom peptide BmKAPi precursor [Mesobuthus martensii]	4.00E-05
comp5641_c0_seq1	CUFF.80185	22.41	31.67	putative RNA binding protein [Opisthacanthus cayaporum]	2.00E-06
comp5710_c0_seq1	CUFF.80968	11.47	30.18	NADH dehydrogenase subunit 2 [Mesobuthus martensii]	2.00E-10
comp5764_c0_seq1	CUFF.81481	19.73	56.00	putative secreted protein [Opisthacanthus cayaporum]	2.00E-35
comp5921_c0_seq1	CUFF.83216	6.52	25.30	putative RNA binding protein [Opisthacanthus cayaporum]	2.00E-05
comp5921_c0_seq2	CUFF.83226	8.12	25.30	putative RNA binding protein [Opisthacanthus cayaporum]	2.00E-05
comp5975_c0_seq1	CUFF.83762	11.47	30.18	NADH dehydrogenase subunit 2 [Mesobuthus martensii]	2.00E-10
comp6005_c0_seq1	CUFF.84037	20.04	78.10	hypothetical protein [Opisthacanthus cayaporum]	4.00E-47
comp6112_c0_seq1	CUFF.85195	4.75	67.40	cytochome oxidase subunit I [Tityus discrepans]	2.00E-71
comp6279_c0_seq1	CUFF.87031	29.87	29.21	venom peptide BmKAPi precursor [Mesobuthus martensii]	4.00E-05
comp6353_c0_seq1	CUFF.87802	13.13	45.45	hypothetical protein [Opisthacanthus cayaporum]	3.00E-06
comp6358_c0_seq1	CUFF.87857	15.36	33.33	putative RNA binding protein [Opisthacanthus cayaporum]	4.00E-11
comp6438_c0_seq2	CUFF.88570	9.23	21.48	putative RNA binding protein [Opisthacanthus cayaporum]	5.00E-05
comp6452_c0_seq1	CUFF.88706	9.37	80.36	hypothetical protein [Opisthacanthus cayaporum]	3.00E-56
comp6606_c0_seq1	CUFF.90309	12.81	45.45	hypothetical protein [Opisthacanthus cayaporum]	3.00E-06
comp6688_c0_seq1	CUFF.91245	15.33	33.33	putative RNA binding protein [Opisthacanthus cayaporum]	4.00E-11
comp6766_c0_seq1	CUFF.92029	11.39	29.85	actin [Opisthacanthus cayaporum]	7.00E-21
comp6772_c0_seq1	CUFF.92111	10.69	44.92	actin [Opisthacanthus cayaporum]	3.00E-41
comp7022_c0_seq1	CUFF.94603	13.44	32.94	venom toxin [Opisthacanthus cayaporum]	3.00E-20
comp7041_c0_seq1	CUFF.94767	5.17	52.16	venom hyaluronidase [Mesobuthus martensii]	3.00E-77
comp7041_c0_seq2	CUFF.94765	6.21	49.06	venom hyaluronidase [Mesobuthus martensii]	8.00E-31
comp7041_c0_seq3	CUFF.94769	2.92	56.10	venom hyaluronidase [Mesobuthus martensii]	4.00E-43
comp7071_c0_seq1	CUFF.95048	4.79	51.18	venom hyaluronidase [Mesobuthus martensii]	3.00E-110
comp7071_c0_seq2	CUFF.95046	6.24	49.06	venom hyaluronidase [Mesobuthus martensii]	9.00E-31
comp7075_c0_seq1	CUFF.95106	10.69	44.92	actin [Opisthacanthus cayaporum]	3.00E-41
comp7276_c0_seq1	CUFF.96934	12.33	30.56	putative RNA binding protein [Opisthacanthus cayaporum]	7.00E-07
comp7390_c0_seq1	CUFF.98003	7.52	30.59	putative RNA binding protein [Opisthacanthus cayaporum]	3.00E-07
comp7448_c0_seq1	CUFF.98465	10.94	53.09	sulfotransferase-like protein [Opisthacanthus cayaporum]	1.00E-64

comp7591_c0_seq1	CUFF.99765	20.27	78.10	hypothetical protein [Opisthacanthus cayaporum]	4.00E-47
comp7821_c0_seq1	CUFF.101779	17.91	33.66	sulfotransferase-like protein [Opisthacanthus cayaporum]	4.00E-35
comp7858_c0_seq1	CUFF.102070	7.48	30.59	putative RNA binding protein [Opisthacanthus cayaporum]	3.00E-07
comp7869_c0_seq1	CUFF.102181	7.52	26.21	elastase-like protein [Opisthacanthus cayaporum]	3.00E-14
comp7935_c0_seq1	CUFF.102839	18.09	33.66	sulfotransferase-like protein [Opisthacanthus cayaporum]	4.00E-35
comp8174_c0_seq2	CUFF.104985	8.81	21.48	putative RNA binding protein [Opisthacanthus cayaporum]	5.00E-05
comp8495_c0_seq1	CUFF.107927	12.08	59.77	cytochrome b [Mesobuthus martensii]	1.00E-103
comp8553_c0_seq1	CUFF.108324	583.12	52.38	cytochrome c oxidase subunit I [Mesobuthus martensii]	6.00E-24
comp8760_c0_seq1	CUFF.110042	12.71	30.56	putative RNA binding protein [Opisthacanthus cayaporum]	7.00E-07
comp8834_c0_seq1	CUFF.110536	12.49	58.82	putative thump domain-containing protein [Opisthacanthus cayaporum]	4.00E-31
comp8908_c0_seq1	CUFF.111237	6.79	58.54	RecName: Full=Protease inhibitor BmKTT-2	9.00E-13
comp8913_c0_seq1	CUFF.111280	7.70	45.10	RecName: Full=Protease inhibitor BmKTT-2	8.00E-12
comp8981_c0_seq1	CUFF.111812	4.48	40.28	putative RNA binding protein [Opisthacanthus cayaporum]	2.00E-09
comp8981_c0_seq2	CUFF.111834	4.73	40.28	putative RNA binding protein [Opisthacanthus cayaporum]	1.00E-09
comp9215_c0_seq1	CUFF.113702	11.15	80.36	hypothetical protein [Opisthacanthus cayaporum]	2.00E-56
comp9335_c0_seq1	CUFF.114567	11.32	53.09	sulfotransferase-like protein [Opisthacanthus cayaporum]	1.00E-64
comp9366_c0_seq1	CUFF.114745	9.76	37.40	Full=Phospholipase A2; Short=HgPLA2;	7.00E-20
comp9382_c0_seq1	CUFF.114864	9.34	92.68	prefoldin-like protein [Opisthacanthus cayaporum]	2.00E-37
comp9452_c0_seq1	CUFF.115515	8.19	35.47	NADH dehydrogenase subunit 5 [Mesobuthus martensii]	3.00E-51
comp9784_c0_seq1	CUFF.117971	10.97	27.54	putative RNA binding protein [Opisthacanthus cayaporum]	3.00E-06
comp9787_c0_seq1	CUFF.118019	6.81	27.40	putative RNA binding protein [Opisthacanthus cayaporum]	2.00E-05
comp9787_c0_seq2	CUFF.117989	3.82	27.40	putative RNA binding protein [Opisthacanthus cayaporum]	7.00E-06
comp9819_c0_seq1	CUFF.118255	4.56	40.28	putative RNA binding protein [Opisthacanthus cayaporum]	1.00E-09
comp9819_c0_seq2	CUFF.118253	4.69	40.28	putative RNA binding protein [Opisthacanthus cayaporum]	1.00E-09
comp9883_c0_seq1	CUFF.118750	29.02	31.25	RecName: Full=Potassium channel toxin alpha-KTx; Flags: Precursor	7.00E-05
comp10032_c0_seq1	CUFF.262	10.39	54.05	inseticidal toxin [Opisthacanthus cayaporum]	4.00E-14
comp10325_c0_seq1	CUFF.2214	12.10	34.18	NADH dehydrogenase subunit 5 [Mesobuthus martensii]	3.00E-17
comp10377_c0_seq1	CUFF.2602	6.59	24.87	beta-actin [Mesobuthus martensii]	9.00E-26
comp10413_c0_seq1	CUFF.2896	8.83	92.68	prefoldin-like protein [Opisthacanthus cayaporum]	2.00E-37
comp10462_c0_seq1	CUFF.3243	9.53	37.40	Full=Phospholipase A2; Short=HgPLA2	7.00E-20
comp10562_c0_seq1	CUFF.3909	13.57	95.24	hypothetical protein [Opisthacanthus cayaporum]	7.00E-22

comp10818_c0_seq1	CUFF.5448	12.14	32.89	NADH dehydrogenase subunit 5 [Mesobuthus martensii]	9.00E-15
comp10946_c0_seq1	CUFF.6244	11.35	27.54	putative RNA binding protein [Opisthacanthus cayaporum]	3.00E-06
comp10966_c0_seq1	CUFF.6354	12.01	58.82	putative thump domain-containing protein [Opisthacanthus cayaporum]	5.00E-31
comp11072_c0_seq1	CUFF.6952	7.54	54.05	inseticidal toxin [Opisthacanthus cayaporum]	4.00E-14
comp11159_c0_seq1	CUFF.7484	26.27	31.25	RecName: Full=Potassium channel toxin alpha-KTx; Flags: Precursor	7.00E-05
comp11257_c0_seq1	CUFF.8153	4.88	29.09	venom toxin [Opisthacanthus cayaporum]	2.00E-13
comp11257_c0_seq2	CUFF.8163	3.70	29.09	venom toxin [Opisthacanthus cayaporum]	2.00E-13
comp11327_c0_seq1	CUFF.8535	17.43	48.36	NADH dehydrogenase subunit 1 [Mesobuthus martensii]	2.00E-24
comp11329_c0_seq1	CUFF.8543	8.53	31.87	putative RNA binding protein [Opisthacanthus cayaporum]	1.00E-10
comp11619_c0_seq1	CUFF.10181	13.46	34.33	putative anticoagulant peptide AP1 [Mesobuthus martensii]	1.00E-05
comp11714_c0_seq1	CUFF.10766	3.79	28.57	putative RNA binding protein [Opisthacanthus cayaporum]	9.00E-08
comp11714_c0_seq2	CUFF.10770	4.30	28.57	putative RNA binding protein [Opisthacanthus cayaporum]	9.00E-08
comp11879_c0_seq1	CUFF.11589	4.74	29.09	venom toxin [Opisthacanthus cayaporum]	2.00E-13
comp11879_c0_seq2	CUFF.11586	3.62	29.09	venom toxin [Opisthacanthus cayaporum]	2.00E-13
comp11977_c0_seq1	CUFF.12109	18.30	44.23	RecName: Full=Protease inhibitor BmKTT-2	4.00E-13
comp12033_c0_seq1	CUFF.12451	16.82	34.07	hypothetical protein [Tityus discrepans]	5.00E-12
comp12154_c0_seq1	CUFF.13116	6.34	25.30	putative RNA binding protein [Opisthacanthus cayaporum]	2.00E-06
comp12154_c0_seq2	CUFF.13118	5.38	25.30	putative RNA binding protein [Opisthacanthus cayaporum]	2.00E-06
comp12411_c0_seq1	CUFF.14307	7.26	28.33	voltage-gated sodium channel protein [Mesobuthus martensii]	2.00E-19
comp12442_c0_seq1	CUFF.14509	9.87	35.03	serpin peptidase inhibitor-like protein [Opisthacanthus cayaporum]	2.00E-26
comp12457_c0_seq1	CUFF.14582	6.10	27.86	voltage-gated sodium channel protein [Mesobuthus martensii]	1.00E-24
comp12474_c0_seq1	CUFF.14697	13.39	66.67	hypothetical protein [Opisthacanthus cayaporum]	7.00E-16
comp12498_c0_seq1	CUFF.14835	8.02	35.29	putative RNA binding protein [Opisthacanthus cayaporum]	1.00E-08
comp12554_c0_seq1	CUFF.15115	18.87	44.23	RecName: Full=Protease inhibitor BmKTT-2	4.00E-13
comp12622_c0_seq1	CUFF.15520	6.38	25.30	putative RNA binding protein [Opisthacanthus cayaporum]	2.00E-06
comp12622_c0_seq2	CUFF.15523	5.04	25.30	putative RNA binding protein [Opisthacanthus cayaporum]	2.00E-06
comp12643_c0_seq1	CUFF.15643	8.83	31.87	putative RNA binding protein [Opisthacanthus cayaporum]	1.00E-10
comp12935_c0_seq1	CUFF.16896	3.78	28.57	putative RNA binding protein [Opisthacanthus cayaporum]	9.00E-08
comp12935_c0_seq2	CUFF.16903	4.29	28.57	putative RNA binding protein [Opisthacanthus cayaporum]	9.00E-08
comp13033_c0_seq1	CUFF.17377	6.38	78.64	sulfotransferase-like protein [Opisthacanthus cayaporum]	3.00E-101
comp13102_c0_seq1	CUFF.17687	17.47	36.36	hypothetical protein [Tityus discrepans]	4.00E-13

comp13137_c0_seq1	CUFF.17891	16.82	34.07	hypothetical protein [Tityus discrepans]	5.00E-12
comp13167_c0_seq1	CUFF.18018	4.63	25.29	putative RNA binding protein [Opisthacanthus cayaporum]	4.00E-06
comp13167_c0_seq2	CUFF.18020	6.34	25.29	putative RNA binding protein [Opisthacanthus cayaporum]	3.00E-06
comp13222_c0_seq1	CUFF.18325	5.55	27.14	voltage-gated sodium channel protein [Mesobuthus martensii]	5.00E-05
comp13229_c0_seq1	CUFF.18330	6.40	28.89	serpin peptidase inhibitor-like protein [Opisthacanthus cayaporum]	2.00E-17
comp13392_c0_seq1	CUFF.18996	8.18	49.19	actin [Opisthacanthus cayaporum]	3.00E-47
comp13736_c0_seq1	CUFF.20461	9.09	38.89	putative RNA binding protein [Opisthacanthus cayaporum]	3.00E-08
comp13767_c0_seq1	CUFF.20619	15.26	38.71	TXLP4 [Mesobuthus martensii]	4.00E-10
comp14155_c0_seq1	CUFF.22030	10.18	49.08	NADH dehydrogenase subunit 1 [Mesobuthus martensii]	1.00E-30
comp14245_c0_seq1	CUFF.22373	13.31	34.33	putative anticoagulant peptide AP1 [Mesobuthus martensii]	1.00E-05
comp14245_c0_seq2	CUFF.22380	10.42	34.92	venom peptide BmKAPi precursor [Mesobuthus martensii]	2.00E-05
comp14247_c0_seq1	CUFF.22389	7.33	26.21	elastase-like protein [Opisthacanthus cayaporum]	3.00E-14
comp14545_c0_seq1	CUFF.23450	8.15	49.19	actin [Opisthacanthus cayaporum]	3.00E-47
comp14775_c0_seq1	CUFF.24292	9.62	43.57	NADH dehydrogenase subunit 4 [Mesobuthus martensii]	8.00E-40
comp14810_c0_seq1	CUFF.24442	5.20	27.40	putative RNA binding protein [Opisthacanthus cayaporum]	1.00E-05
comp14810_c0_seq2	CUFF.24435	3.97	27.40	putative RNA binding protein [Opisthacanthus cayaporum]	6.00E-06
comp14810_c0_seq3	CUFF.24438	5.24	27.40	putative RNA binding protein [Opisthacanthus cayaporum]	5.00E-06
comp14862_c0_seq1	CUFF.24653	9.06	39.62	PinX1 [Mesobuthus martensii]	3.00E-05
comp14979_c0_seq1	CUFF.25068	7.83	46.03	putative neurotoxin Na8 precursor [Tityus discrepans]	4.00E-12
comp15037_c0_seq1	CUFF.25280	5.80	27.14	voltage-gated sodium channel protein [Mesobuthus martensii]	7.00E-05
comp15134_c0_seq1	CUFF.25549	5.81	24.53	elastase-like protein [Opisthacanthus cayaporum]	5.00E-07
comp15190_c0_seq1	CUFF.25716	4.35	25.29	putative RNA binding protein [Opisthacanthus cayaporum]	3.00E-06
comp15190_c0_seq2	CUFF.25720	6.21	25.29	putative RNA binding protein [Opisthacanthus cayaporum]	3.00E-06
comp15306_c0_seq1	CUFF.26001	9.51	35.40	NADH dehydrogenase subunit 2 [Mesobuthus martensii]	2.00E-20
comp15339_c0_seq1	CUFF.26100	5.15	81.82	hypothetical protein [Tityus discrepans]	5.00E-05
comp15486_c0_seq1	CUFF.26526	14.42	38.71	TXLP4 [Mesobuthus martensii]	4.00E-10
comp15562_c0_seq1	CUFF.26765	6.53	29.38	serpin peptidase inhibitor-like protein [Opisthacanthus cayaporum]	9.00E-17
comp15627_c0_seq1	CUFF.26931	5.76	34.66	actin [Opisthacanthus cayaporum]	1.00E-29
comp15723_c0_seq1	CUFF.27183	5.37	24.53	elastase-like protein [Opisthacanthus cayaporum]	6.00E-07
comp15749_c0_seq1	CUFF.27319	6.39	98.06	putative Ca2+-dependent activator protein [Opisthacanthus cayaporum]	2.00E-53
comp15861_c0_seq1	CUFF.27658	7.70	32.35	shaker cognate b [Mesobuthus martensii]	2.00E-05

comp15924_c0_seq1	CUFF.27803	11.11	45.41	NADH dehydrogenase subunit 4 [Mesobuthus martensii]	2.00E-27
comp15988_c0_seq1	CUFF.27970	4.25	48.72	toll receptor, partial [Mesobuthus eupeus]	2.00E-05
comp15988_c0_seq2	CUFF.27971	1.39	48.72	toll receptor, partial [Mesobuthus eupeus]	2.00E-05
comp16042_c0_seq1	CUFF.28096	10.02	35.03	serpin peptidase inhibitor-like protein [Opisthacanthus cayaporum]	2.00E-26
comp16178_c0_seq1	CUFF.28515	5.96	98.06	putative Ca2+-dependent activator protein [Opisthacanthus cayaporum]	2.00E-53
comp16329_c0_seq1	CUFF.28906	6.16	34.94	actin [Opisthacanthus cayaporum]	2.00E-28
comp16539_c0_seq1	CUFF.29438	7.70	32.35	shaker cognate b [Mesobuthus martensii]	2.00E-05
comp16708_c0_seq1	CUFF.29767	8.89	39.62	PinX1 [Mesobuthus martensii]	3.00E-05
comp16711_c0_seq1	CUFF.29777	5.31	75.00	toll receptor, partial [Mesobuthus eupeus]	5.00E-05
comp16713_c0_seq1	CUFF.29779	31.88	39.39	hypothetical protein [Tityus discrepans]	3.00E-11
comp16785_c0_seq1	CUFF.29936	6.30	24.87	beta-actin [Mesobuthus martensii]	9.00E-26
comp16913_c0_seq1	CUFF.30253	9.13	38.89	putative RNA binding protein [Opisthacanthus cayaporum]	3.00E-08
comp17117_c0_seq1	CUFF.30749	7.91	31.03	PinX1 [Mesobuthus martensii]	9.00E-10
comp17133_c0_seq1	CUFF.30782	2.19	48.72	toll receptor, partial [Mesobuthus eupeus]	2.00E-05
comp17133_c0_seq2	CUFF.30784	1.96	48.72	toll receptor, partial [Mesobuthus eupeus]	2.00E-05
comp17177_c0_seq1	CUFF.30908	4.89	36.99	putative RNA binding protein [Opisthacanthus cayaporum]	2.00E-09
comp17487_c0_seq1	CUFF.31612	6.66	35.77	NADH dehydrogenase subunit 5 [Mesobuthus martensii]	9.00E-44
comp17675_c0_seq1	CUFF.31944	5.49	46.03	putative neurotoxin Na8 precursor [Tityus discrepans]	4.00E-12
comp17675_c0_seq2	CUFF.31947	6.80	47.17	putative neurotoxin Na8 precursor [Tityus discrepans]	1.00E-10
comp17858_c0_seq1	CUFF.32372	10.48	39.06	insect beta-neurotoxin [Mesobuthus martensii]	5.00E-10
comp18425_c0_seq1	CUFF.33723	10.32	39.06	insect beta-neurotoxin [Mesobuthus martensii]	5.00E-10
comp18498_c0_seq1	CUFF.33843	3.92	36.84	Full=Phospholipase A2; Short=HgPLA2	8.00E-11
comp18498_c0_seq2	CUFF.33849	4.75	36.84	Full=Phospholipase A2; Short=HgPLA2; subunit	8.00E-11
comp18538_c0_seq1	CUFF.33910	3.75	27.85	elastase-like protein [Opisthacanthus cayaporum]	3.00E-14
comp18666_c0_seq1	CUFF.34142	4.77	36.84	Full=Phospholipase A2; Short=HgPLA2	8.00E-11
comp18720_c0_seq1	CUFF.34258	9.02	35.40	NADH dehydrogenase subunit 2 [Mesobuthus martensii]	2.00E-20
comp18932_c0_seq1	CUFF.34640	11.36	51.61	NADH dehydrogenase subunit 1 [Mesobuthus martensii]	3.00E-14
comp19155_c0_seq1	CUFF.35153	6.25	30.48	elastase-like protein [Opisthacanthus cayaporum]	2.00E-08
comp19620_c0_seq1	CUFF.35936	9.44	49.56	sulfotransferase-like protein [Opisthacanthus cayaporum]	2.00E-31
comp19620_c0_seq2	CUFF.35938	46.23	49.12	sulfotransferase-like protein [Opisthacanthus cayaporum]	1.00E-32
comp19891_c0_seq1	CUFF.36354	9.35	41.89	NADH dehydrogenase subunit 4 [Mesobuthus martensii]	3.00E-10

comp19937_c0_seq1	CUFF.36436	6.12	30.48	elastase-like protein [Opisthacanthus cayaporum]	2.00E-08
comp20028_c0_seq1	CUFF.36566	4.68	34.04	voltage-gated sodium channel protein [Mesobuthus martensii]	1.00E-19
comp20594_c0_seq1	CUFF.37438	8.44	31.03	PinX1 [Mesobuthus martensii]	9.00E-10
comp21176_c0_seq1	CUFF.38352	6.04	43.59	actin [Opisthacanthus cayaporum]	1.00E-42
comp21711_c0_seq1	CUFF.39053	7.98	36.84	putative RNA binding protein [Opisthacanthus cayaporum]	2.00E-07
comp21903_c0_seq1	CUFF.39295	3.82	28.50	elastase-like protein [Opisthacanthus cayaporum]	2.00E-12
comp21903_c0_seq2	CUFF.39298	3.96	28.50	elastase-like protein [Opisthacanthus cayaporum]	2.00E-12
comp22378_c0_seq1	CUFF.39901	10.47	35.71	JCH2 precursor [Mesobuthus martensii]	2.00E-06
comp22567_c0_seq1	CUFF.40149	6.16	27.93	hypothetical protein [Opisthacanthus cayaporum]	7.00E-05
comp22581_c0_seq1	CUFF.40175	8.94	28.38	putative RNA binding protein [Opisthacanthus cayaporum]	6.00E-08
comp22789_c0_seq1	CUFF.40476	6.69	26.92	putative RNA binding protein [Opisthacanthus cayaporum]	1.00E-05
comp23080_c0_seq1	CUFF.40856	7.75	45.45	sulfotransferase-like protein [Opisthacanthus cayaporum]	2.00E-10
comp23080_c0_seq2	CUFF.40858	7.76	48.89	sulfotransferase-like protein [Opisthacanthus cayaporum]	7.00E-12
comp23221_c0_seq1	CUFF.41030	4.73	32.00	putative RNA binding protein [Opisthacanthus cayaporum]	1.00E-06
comp23488_c0_seq1	CUFF.41324	6.05	27.93	hypothetical protein [Opisthacanthus cayaporum]	8.00E-05
comp23604_c0_seq1	CUFF.41496	5.69	27.63	putative RNA binding protein [Opisthacanthus cayaporum]	2.00E-05
comp23962_c0_seq1	CUFF.41942	4.11	28.50	elastase-like protein [Opisthacanthus cayaporum]	2.00E-12
comp24193_c0_seq1	CUFF.42214	4.65	92.47	actin [Opisthacanthus cayaporum]	1.00E-98
comp24198_c0_seq1	CUFF.42223	6.41	26.92	putative RNA binding protein [Opisthacanthus cayaporum]	1.00E-05
comp24209_c0_seq1	CUFF.42234	8.54	31.17	JCH2 precursor [Mesobuthus martensii]	4.00E-06
comp24335_c0_seq1	CUFF.42400	6.00	75.00	toll receptor, partial [Mesobuthus eupeus]	3.00E-05
comp24367_c0_seq1	CUFF.42441	10.23	62.96	NADH dehydrogenase subunit 3 [Mesobuthus martensii]	3.00E-07
comp24525_c0_seq1	CUFF.42617	5.43	27.63	putative RNA binding protein [Opisthacanthus cayaporum]	2.00E-05
comp24827_c0_seq1	CUFF.43041	4.97	50.00	RecName: Full=Protease inhibitor BmKTT-3	6.00E-14
comp25140_c0_seq1	CUFF.43441	7.37	28.38	putative RNA binding protein [Opisthacanthus cayaporum]	1.00E-07
comp25479_c0_seq1	CUFF.43858	5.71	27.27	hypothetical protein [Opisthacanthus cayaporum]	2.00E-06
comp25574_c0_seq1	CUFF.43986	6.72	29.77	beta-actin [Mesobuthus martensii]	2.00E-14
comp25959_c0_seq1	CUFF.44471	6.62	48.08	sulfotransferase-like protein [Opisthacanthus cayaporum]	2.00E-13
comp25959_c0_seq2	CUFF.44472	6.04	49.06	sulfotransferase-like protein [Opisthacanthus cayaporum]	2.00E-14
comp26056_c0_seq1	CUFF.44575	3.60	30.53	beta-actin [Mesobuthus martensii]	5.00E-17
comp26130_c0_seq1	CUFF.44688	4.69	34.04	voltage-gated sodium channel protein [Mesobuthus martensii]	1.00E-19

comp26164_c0_seq1	CUFF.44723	14.62	86.67	voltage-gated sodium channel protein [Mesobuthus martensii]	4.00E-32
comp26191_c0_seq1	CUFF.44786	5.98	43.59	actin [Opisthacanthus cayaporum]	1.00E-42
comp26723_c0_seq1	CUFF.45453	4.63	28.05	putative RNA binding protein [Opisthacanthus cayaporum]	2.00E-06
comp27239_c0_seq1	CUFF.46202	4.55	27.68	putative RNA binding protein [Opisthacanthus cayaporum]	7.00E-11
comp27656_c0_seq1	CUFF.46706	6.54	27.27	hypothetical protein [Opisthacanthus cayaporum]	2.00E-06
comp27687_c0_seq1	CUFF.46736	4.48	31.32	venom toxin [Opisthacanthus cayaporum]	2.00E-17
comp28324_c0_seq1	CUFF.47529	6.33	50.00	sulfotransferase-like protein [Opisthacanthus cayaporum]	6.00E-15
comp28683_c0_seq1	CUFF.47942	11.44	40.00	NADH dehydrogenase subunit 3 [Mesobuthus martensii]	2.00E-07
comp28970_c0_seq1	CUFF.48326	3.46	30.53	beta-actin [Mesobuthus martensii]	5.00E-17
comp29594_c0_seq1	CUFF.49146	4.36	49.02	RecName: Full=Protease inhibitor BmKTT-2	3.00E-14
comp29819_c0_seq1	CUFF.49401	4.48	87.67	voltage-gated sodium channel protein [Mesobuthus martensii]	1.00E-104
comp30328_c0_seq1	CUFF.50011	5.83	47.11	beta-actin [Mesobuthus martensii]	6.00E-28
comp30406_c0_seq1	CUFF.50120	4.72	31.32	venom toxin [Opisthacanthus cayaporum]	4.00E-17
comp30489_c0_seq1	CUFF.50233	4.53	28.05	putative RNA binding protein [Opisthacanthus cayaporum]	2.00E-06
comp31512_c0_seq1	CUFF.51548	3.25	27.71	putative RNA binding protein [Opisthacanthus cayaporum]	4.00E-07
comp31672_c0_seq1	CUFF.51877	5.73	83.46	sulfotransferase-like protein [Opisthacanthus cayaporum]	7.00E-65
comp31767_c0_seq1	CUFF.51975	4.46	27.68	putative RNA binding protein [Opisthacanthus cayaporum]	7.00E-11
comp31957_c0_seq1	CUFF.52191	4.59	39.85	PinX1 [Mesobuthus martensii]	1.00E-17
comp32397_c0_seq2	CUFF.52728	4.36	36.84	PinX1 [Mesobuthus martensii]	7.00E-05
comp32730_c0_seq1	CUFF.53095	3.22	32.73	PinX1 [Mesobuthus martensii]	4.00E-05
comp32979_c0_seq1	CUFF.53379	5.10	50.00	RecName: Full=Protease inhibitor BmKTT-3	5.00E-14
comp33864_c0_seq1	CUFF.54445	4.91	30.34	actin [Opisthacanthus cayaporum]	1.00E-19
comp33867_c0_seq1	CUFF.54450	5.16	26.67	putative RNA binding protein [Opisthacanthus cayaporum]	8.00E-06
comp33983_c0_seq1	CUFF.54590	4.33	92.47	actin [Opisthacanthus cayaporum]	8.00E-99
comp34529_c0_seq1	CUFF.55231	3.26	30.38	putative RNA binding protein [Opisthacanthus cayaporum]	4.00E-05
comp34529_c0_seq2	CUFF.55234	3.87	30.38	putative RNA binding protein [Opisthacanthus cayaporum]	3.00E-05
comp34837_c0_seq1	CUFF.55659	3.79	33.33	makatoxin II precursor [Mesobuthus martensii]	7.00E-05
comp35237_c0_seq1	CUFF.56157	4.47	49.02	RecName: Full=Protease inhibitor BmKTT-2	3.00E-14
comp36111_c0_seq1	CUFF.57126	4.61	31.98	serpin peptidase inhibitor-like protein [Opisthacanthus cayaporum]	3.00E-16
comp36385_c0_seq1	CUFF.57525	5.13	44.12	putative RNA binding protein [Opisthacanthus cayaporum]	3.00E-05
comp36392_c0_seq1	CUFF.57544	13.97	74.67	sulfotransferase-like protein [Opisthacanthus cayaporum]	7.00E-33

comp37154_c0_seq1	CUFF.58358	3.38	27.54	elastase-like protein [Opisthacanthus cayaporum]	2.00E-09
comp38082_c0_seq1	CUFF.59456	4.85	26.67	putative RNA binding protein [Opisthacanthus cayaporum]	7.00E-06
comp38197_c0_seq1	CUFF.59582	5.25	47.93	sulfotransferase-like protein [Opisthacanthus cayaporum]	1.00E-34
comp38299_c0_seq1	CUFF.59704	3.49	27.71	putative RNA binding protein [Opisthacanthus cayaporum]	4.00E-07
comp38507_c0_seq1	CUFF.60132	3.44	36.36	sulfotransferase-like protein [Opisthacanthus cayaporum]	5.00E-19
comp39496_c0_seq1	CUFF.61244	5.15	71.88	calmodulin-like protein [Opisthacanthus cayaporum]	1.00E-10
comp39695_c0_seq1	CUFF.61456	3.32	31.98	serpin peptidase inhibitor-like protein [Opisthacanthus cayaporum]	4.00E-17
comp40253_c0_seq1	CUFF.62062	5.22	30.20	beta-actin [Mesobuthus martensii]	8.00E-16
comp40497_c0_seq1	CUFF.62341	3.57	88.89	actin [Opisthacanthus cayaporum]	7.00E-60
comp41538_c0_seq1	CUFF.63544	4.24	26.87	beta-actin [Mesobuthus martensii]	3.00E-28
comp41624_c0_seq1	CUFF.63634	5.27	50.55	putative secreted protein [Opisthacanthus cayaporum]	7.00E-29
comp41691_c0_seq1	CUFF.63707	8.16	73.08	ferritin-like peptide [Opisthacanthus cayaporum]	1.00E-08
comp41894_c0_seq1	CUFF.63896	19.98	86.21	voltage-gated sodium channel protein [Mesobuthus martensii]	1.00E-30
comp42213_c0_seq1	CUFF.64242	4.64	44.44	putative RNA binding protein [Opisthacanthus cayaporum]	4.00E-06
comp43244_c0_seq1	CUFF.65391	4.44	27.17	beta-actin [Mesobuthus martensii]	4.00E-25
comp43806_c0_seq1	CUFF.66075	6.38	49.15	sulfotransferase-like protein [Opisthacanthus cayaporum]	4.00E-16
comp45009_c0_seq1	CUFF.67359	4.30	33.33	makatoxin II precursor [Mesobuthus martensii]	9.00E-05
comp45315_c0_seq1	CUFF.67776	2.75	35.24	venom toxin [Opisthacanthus cayaporum]	2.00E-10
comp45315_c0_seq2	CUFF.67778	2.72	34.78	venom toxin [Opisthacanthus cayaporum]	3.00E-11
comp45929_c0_seq1	CUFF.68402	4.16	38.89	PinX1 [Mesobuthus martensii]	2.00E-16
comp45940_c0_seq1	CUFF.68418	3.17	30.38	putative RNA binding protein [Opisthacanthus cayaporum]	4.00E-05
comp45940_c0_seq2	CUFF.68423	3.29	30.38	putative RNA binding protein [Opisthacanthus cayaporum]	4.00E-05
comp46389_c0_seq1	CUFF.68948	4.64	31.91	sulfotransferase-like protein [Opisthacanthus cayaporum]	1.00E-05
comp46499_c0_seq1	CUFF.69045	4.08	34.67	putative RNA binding protein [Opisthacanthus cayaporum]	2.00E-09
comp46671_c0_seq1	CUFF.69222	3.85	85.29	voltage-gated sodium channel protein [Mesobuthus martensii]	7.00E-50
comp46671_c0_seq2	CUFF.69224	5.98	85.37	voltage-gated sodium channel protein [Mesobuthus martensii]	5.00E-40
comp46707_c0_seq1	CUFF.69277	3.90	34.67	putative RNA binding protein [Opisthacanthus cayaporum]	2.00E-09
comp46752_c0_seq1	CUFF.69341	3.75	27.69	putative RNA binding protein [Opisthacanthus cayaporum]	2.00E-07
comp47320_c0_seq1	CUFF.69996	6.33	54.22	putative secreted protein [Opisthacanthus cayaporum]	2.00E-28
comp49302_c0_seq1	CUFF.72374	5.39	38.46	serpin peptidase inhibitor-like protein [Opisthacanthus cayaporum]	4.00E-08
comp49548_c0_seq1	CUFF.72661	3.21	32.26	putative RNA binding protein [Opisthacanthus cayaporum]	2.00E-10

comp49740_c0_seq1	CUFF.72876	3.01	24.54	serpin peptidase inhibitor-like protein [Opisthacanthus cayaporum]	3.00E-07
comp49962_c0_seq1	CUFF.73082	5.01	71.88	calmodulin-like protein [Opisthacanthus cayaporum]	1.00E-10
comp50532_c0_seq1	CUFF.73798	6.68	38.89	NADH dehydrogenase subunit 3 [Mesobuthus martensii]	1.00E-07
comp50552_c0_seq1	CUFF.73835	3.74	87.95	voltage-gated sodium channel protein [Mesobuthus martensii]	9.00E-148
comp51530_c0_seq1	CUFF.74852	3.26	24.05	serpin peptidase inhibitor-like protein [Opisthacanthus cayaporum]	3.00E-06
comp54062_c0_seq1	CUFF.77683	5.81	25.00	serpin peptidase inhibitor-like protein [Opisthacanthus cayaporum]	4.00E-07
comp54130_c0_seq1	CUFF.77747	5.17	33.94	NADH dehydrogenase subunit 5 [Mesobuthus martensii]	5.00E-11
comp54217_c0_seq1	CUFF.77810	3.91	91.40	actin [Opisthacanthus cayaporum]	2.00E-98
comp54236_c0_seq1	CUFF.77828	4.40	26.67	putative RNA binding protein [Opisthacanthus cayaporum]	4.00E-05
comp55750_c0_seq1	CUFF.79522	4.75	32.97	serpin peptidase inhibitor-like protein [Opisthacanthus cayaporum]	1.00E-10
comp56344_c0_seq1	CUFF.80093	2.97	27.54	voltage-gated sodium channel protein [Mesobuthus martensii]	7.00E-13
comp56609_c0_seq1	CUFF.80387	3.19	31.25	serpin peptidase inhibitor-like protein [Opisthacanthus cayaporum]	1.00E-07
comp56664_c0_seq1	CUFF.80449	4.08	85.29	voltage-gated sodium channel protein [Mesobuthus martensii]	7.00E-50
comp57516_c0_seq1	CUFF.81354	4.62	33.33	serpin peptidase inhibitor-like protein [Opisthacanthus cayaporum]	7.00E-14
comp59398_c0_seq1	CUFF.83378	6.48	53.85	toll receptor, partial [Mesobuthus eupeus]	2.00E-06
comp59715_c0_seq1	CUFF.83688	5.47	47.93	sulfotransferase-like protein [Opisthacanthus cayaporum]	1.00E-34
comp59760_c0_seq1	CUFF.83745	4.54	36.17	elastase-like protein [Opisthacanthus cayaporum]	3.00E-07
comp60059_c0_seq1	CUFF.84000	4.35	30.77	serpin peptidase inhibitor-like protein [Opisthacanthus cayaporum]	4.00E-06
comp60433_c0_seq1	CUFF.84416	5.02	39.13	serpin peptidase inhibitor-like protein [Opisthacanthus cayaporum]	2.00E-06
comp62513_c0_seq1	CUFF.86744	2.98	36.36	sulfotransferase-like protein [Opisthacanthus cayaporum]	5.00E-19
comp62833_c0_seq1	CUFF.87052	7.89	75.00	ferritin-like peptide [Opisthacanthus cayaporum]	5.00E-08
comp64247_c0_seq1	CUFF.88453	5.98	47.11	beta-actin [Mesobuthus martensii]	6.00E-28
comp64806_c0_seq1	CUFF.89000	10.51	79.55	voltage-gated sodium channel protein [Mesobuthus martensii]	2.00E-18
comp65231_c0_seq1	CUFF.89473	3.57	35.14	AS neurotoxin precursor [Mesobuthus martensii]	9.00E-05
comp65438_c0_seq1	CUFF.89702	3.34	42.50	shaker cognate b [Mesobuthus martensii]	4.00E-07
comp65583_c0_seq1	CUFF.89830	5.71	63.41	voltage-gated sodium channel protein [Mesobuthus martensii]	1.00E-26
comp66223_c0_seq1	CUFF.90447	3.45	32.26	putative RNA binding protein [Opisthacanthus cayaporum]	3.00E-10
comp66254_c0_seq1	CUFF.90490	30.98	46.00	NADH dehydrogenase subunit 1 [Mesobuthus martensii]	2.00E-08
comp66338_c0_seq1	CUFF.90622	4.12	36.17	RecName: Full=Serine proteinase-like BMK-CBP	3.00E-06
comp66519_c0_seq1	CUFF.90835	3.82	26.67	putative RNA binding protein [Opisthacanthus cayaporum]	4.00E-05
comp70052_c0_seq1	CUFF.94445	7.21	80.77	voltage-gated sodium channel protein [Mesobuthus martensii]	2.00E-22

comp70495_c0_seq1	CUFF.94827	4.16	31.82	NADH dehydrogenase subunit 5 [Mesobuthus martensii]	3.00E-13
comp71326_c0_seq1	CUFF.95596	3.00	28.71	serpin peptidase inhibitor-like protein [Opisthacanthus cayaporum]	4.00E-10
comp71520_c0_seq1	CUFF.95795	12.24	34.38	hypothetical protein [Tityus discrepans]	4.00E-05
comp71780_c0_seq1	CUFF.96085	4.57	33.33	serpin peptidase inhibitor-like protein [Opisthacanthus cayaporum]	7.00E-14
comp72450_c0_seq1	CUFF.96673	3.44	31.15	PinX1 [Mesobuthus martensii]	1.00E-04
comp73481_c0_seq1	CUFF.97600	5.24	29.67	voltage-gated sodium channel protein [Mesobuthus martensii]	4.00E-18
comp73501_c0_seq1	CUFF.97619	3.97	34.29	serpin peptidase inhibitor-like protein [Opisthacanthus cayaporum]	1.00E-19
comp73647_c0_seq1	CUFF.97755	9.80	31.08	elastase-like protein [Opisthacanthus cayaporum]	4.00E-09
comp75842_c0_seq1	CUFF.99710	6.52	33.90	venom peptide BmKAPi precursor [Mesobuthus martensii]	9.00E-05
comp76298_c0_seq1	CUFF.100056	4.75	34.09	serpin peptidase inhibitor-like protein [Opisthacanthus cayaporum]	1.00E-11
comp76392_c0_seq1	CUFF.100123	2.84	33.33	serpin peptidase inhibitor-like protein [Opisthacanthus cayaporum]	4.00E-07
comp77935_c0_seq1	CUFF.101528	6.07	35.42	putative RNA binding protein [Opisthacanthus cayaporum]	5.00E-06
comp79281_c0_seq1	CUFF.102729	4.04	30.67	venom toxin [Opisthacanthus cayaporum]	7.00E-13
comp79654_c0_seq1	CUFF.103069	4.68	47.86	sulfotransferase-like protein [Opisthacanthus cayaporum]	7.00E-34
comp79660_c0_seq1	CUFF.103074	8.42	63.73	voltage-gated sodium channel protein [Mesobuthus martensii]	3.00E-36
comp79719_c0_seq1	CUFF.103134	9.14	32.84	putative secreted protein [Opisthacanthus cayaporum]	7.00E-05
comp79740_c0_seq1	CUFF.103152	7.73	73.21	cytochrome oxidase subunit I [Mesobuthus martensii]	2.00E-18
comp80952_c0_seq1	CUFF.104154	5.14	58.06	RecName: Full=Potassium channel toxin alpha-KTx 6.14; AltName: Full=HgeTx1	2.00E-08
comp81045_c0_seq1	CUFF.104239	4.27	38.18	hypothetical protein [Tityus discrepans]	4.00E-05
comp81212_c0_seq1	CUFF.104397	4.35	36.99	putative RNA binding protein [Opisthacanthus cayaporum]	3.00E-10
comp81356_c0_seq1	CUFF.104565	7.44	38.89	NADH dehydrogenase subunit 3 [Mesobuthus martensii]	1.00E-07
comp85204_c0_seq1	CUFF.108084	7.44	58.57	PinX1 [Mesobuthus martensii]	2.00E-22
comp87378_c0_seq1	CUFF.109844	5.07	28.26	beta-actin [Mesobuthus martensii]	5.00E-08
comp87498_c0_seq1	CUFF.109937	5.27	53.85	toll receptor, partial [Mesobuthus eupeus]	1.00E-06
comp90632_c0_seq1	CUFF.112375	7.50	37.29	putative anticoagulant peptide AP1 [Mesobuthus martensii]	1.00E-05
comp92523_c0_seq1	CUFF.113941	4.68	36.17	elastase-like protein [Opisthacanthus cayaporum]	9.00E-08
comp93409_c0_seq1	CUFF.114587	3.02	27.84	beta-actin [Mesobuthus martensii]	6.00E-09
comp94032_c0_seq1	CUFF.115031	4.03	38.18	hypothetical protein [Tityus discrepans]	3.00E-05
comp94101_c0_seq1	CUFF.115095	11.69	41.30	insect beta-neurotoxin [Mesobuthus martensii]	1.00E-07
comp94467_c0_seq1	CUFF.115459	4.18	32.43	beta-actin [Mesobuthus martensii]	6.00E-06
comp95090_c0_seq1	CUFF.115976	4.99	31.25	hypothetical protein [Opisthacanthus cayaporum]	5.00E-11

comp95308_c0_seq1	CUFF.116176	5.93	51.28	RecName: Full=Protease inhibitor Hg1; Flags: Precursor	3.00E-10
comp95418_c0_seq1	CUFF.116277	4.38	33.33	hypothetical protein [Opisthacanthus cayaporum]	2.00E-13
comp96020_c0_seq1	CUFF.116639	3.71	58.97	shaker cognate b [Mesobuthus martensii]	6.00E-26
comp96025_c0_seq1			97.18	beta-actin [Mesobuthus martensii]	5.00E-40
comp98494_c0_seq1			86.57	voltage-gated sodium channel protein [Mesobuthus martensii]	5.00E-32
comp98713_c0_seq1	CUFF.118624	2.84	21.57	phospholipase C-like protein [Opisthacanthus cayaporum]	8.00E-07
comp98777_c0_seq1	CUFF.118683	2.98	55.07	cytochrome c oxidase subunit I [Mesobuthus martensii]	6.00E-19
comp103035_c0_seq1	CUFF.2090	5.64	70.18	voltage-gated sodium channel protein [Mesobuthus martensii]	6.00E-44
comp103450_c0_seq1			32.84	putative secreted protein [Opisthacanthus cayaporum]	7.00E-05
comp103619_c0_seq1	CUFF.2453	2.94	23.66	hypothetical protein [Opisthacanthus cayaporum]	8.00E-05
comp103716_c0_seq1	CUFF.2524	8.13	44.44	voltage-gated sodium channel protein [Mesobuthus martensii]	3.00E-06
comp103743_c0_seq1	CUFF.2572	4.09	27.91	elastase-like protein [Opisthacanthus cayaporum]	7.00E-07
comp103752_c0_seq1	CUFF.2573	15.32	45.90	sulfotransferase-like protein [Opisthacanthus cayaporum]	2.00E-13
comp104748_c0_seq1	CUFF.3295	11.61	48.15	putative potassium ion channel blocker TXKbeta2' [Mesobuthus martensii]	9.00E-05
comp107516_c0_seq1	CUFF.5022	6.95	54.55	hypothetical protein [Opisthacanthus cayaporum]	1.00E-13
comp109211_c0_seq1	CUFF.6097	6.49	52.38	RecName: Full=Protease inhibitor Hg1; Flags: Precursor	7.00E-12
comp109436_c0_seq1	CUFF.6221	3.33	29.31	serpin peptidase inhibitor-like protein [Opisthacanthus cayaporum]	3.00E-11
comp109662_c0_seq1	CUFF.6334	3.74	32.43	beta-actin [Mesobuthus martensii]	6.00E-06
comp111278_c0_seq1	CUFF.7270	7.15	44.44	voltage-gated sodium channel protein [Mesobuthus martensii]	3.00E-06
comp111980_c0_seq1	CUFF.7740	2.98	32.04	serpin peptidase inhibitor-like protein [Opisthacanthus cayaporum]	4.00E-11
comp112034_c0_seq1	CUFF.7789	3.93	37.29	putative anticoagulant peptide AP1 [Mesobuthus martensii]	1.00E-05
comp113289_c0_seq1	CUFF.8537	3.87	48.44	NADH dehydrogenase subunit 4 [Mesobuthus martensii]	6.00E-11
comp113393_c0_seq1			42.50	shaker cognate b [Mesobuthus martensii]	1.00E-07
comp116677_c0_seq1	CUFF.10459	5.59	54.55	hypothetical protein [Opisthacanthus cayaporum]	1.00E-13
comp118599_c0_seq1	CUFF.11503	3.32	30.77	beta-actin [Mesobuthus martensii]	3.00E-12
comp119493_c0_seq1	CUFF.11903	3.87	32.14	voltage-gated sodium channel protein [Mesobuthus martensii]	4.00E-06
comp120806_c0_seq1	CUFF.12715	4.03	49.55	venom hyaluronidase [Mesobuthus martensii]	1.00E-31
comp121358_c0_seq1	CUFF.13000	7.58	75.95	cytochrome c oxidase subunit I [Mesobuthus martensii]	6.00E-16
comp121776_c0_seq1	CUFF.13232	4.10	25.96	serpin peptidase inhibitor-like protein [Opisthacanthus cayaporum]	6.00E-07
comp124936_c0_seq1	CUFF.14800	3.12	94.59	actin [Opisthacanthus cayaporum]	5.00E-37
comp127074_c0_seq1	CUFF.15940	5.26	40.74	voltage-gated sodium channel protein [Mesobuthus martensii]	2.00E-09

comp127919_c0_seq1	CUFF.16287	6.29	39.74	voltage-gated sodium channel protein [Mesobuthus martensii]	2.00E-11
comp128464_c0_seq1			33.82	voltage-gated sodium channel protein [Mesobuthus martensii]	6.00E-06
comp129747_c0_seq1	CUFF.17068	2.90	55.07	cytochrome c oxidase subunit I [Mesobuthus martensii]	6.00E-19
comp130609_c0_seq1	CUFF.17502	5.02	33.98	voltage-gated sodium channel protein [Mesobuthus martensii]	6.00E-11
comp131256_c0_seq1	CUFF.17810	4.34	59.68	shaker cognate b [Mesobuthus martensii]	7.00E-35
comp131628_c0_seq1			81.58	voltage-gated sodium channel protein [Mesobuthus martensii]	7.00E-34
comp131856_c0_seq1	CUFF.18132	4.59	30.77	serpin peptidase inhibitor-like protein [Opisthacanthus cayaporum]	4.00E-06
comp132883_c0_seq1			49.33	sulfotransferase-like protein [Opisthacanthus cayaporum]	6.00E-22
comp135491_c0_seq1	CUFF.19648	6.51	33.90	venom peptide BmKAPi precursor [Mesobuthus martensii]	9.00E-05
comp137078_c0_seq1	CUFF.20355	9.74	40.00	RecName: Full=Protease inhibitor Hg1; Flags: Precursor	2.00E-10
comp144434_c0_seq1	CUFF.23092	5.06	27.59	putative RNA binding protein [Opisthacanthus cayaporum]	9.00E-06
comp152834_c0_seq1	CUFF.25954	3.01	35.78	voltage-gated sodium channel protein [Mesobuthus martensii]	2.00E-14
comp153278_c0_seq1	CUFF.26041	3.18	32.64	voltage-gated sodium channel protein [Mesobuthus martensii]	6.00E-25
comp153765_c0_seq1	CUFF.26182	6.63	40.66	voltage-gated sodium channel protein [Mesobuthus martensii]	6.00E-16
comp158721_c0_seq1			97.01	beta-actin [Mesobuthus martensii]	1.00E-37
comp161271_c0_seq1	CUFF.28345	7.73	46.55	voltage-gated sodium channel protein [Mesobuthus martensii]	2.00E-07
comp166608_c0_seq1	CUFF.29670	2.59	42.00	NADH dehydrogenase subunit 5 [Mesobuthus martensii]	2.00E-20
comp175038_c0_seq1			38.57	voltage-gated sodium channel protein [Mesobuthus martensii]	3.00E-11
comp187571_c0_seq1	CUFF.34331	7.14	45.35	sulfotransferase-like protein [Opisthacanthus cayaporum]	7.00E-20
comp189517_c0_seq1	CUFF.34681	3.78	30.38	beta-actin [Mesobuthus martensii]	2.00E-12
